# Bridging Horizontal to Vertical Radiative Cooling

**DOI:** 10.1002/advs.77392

**Published:** 2026-08-24

**Authors:** Xiangyu Meng, Gan Huang

**Affiliations:** ^1^ Institute of Microstructure Technology Karlsruhe Institute of Technology Eggenstein‐Leopoldshafen Germany; ^2^ School of Chemistry and Chemical Engineering Southeast University Nanjing Jiangsu China

**Keywords:** building energy savings, daylighting, directional reflectors, radiative cooling, vertical facades, window retrofits

## Abstract

Radiative cooling materials enable sub‐ambient passive cooling but are typically limited to horizontal surfaces such as rooftops, restricting their use in dense urban environments where vertical facades dominate. Here, we develop a scalable BSDAR bridging system that enables existing radiative cooling materials to function effectively on vertical surfaces. The system redirects thermal radiation toward the sky while suppressing heat gain from surrounding buildings and the ground. On vertical windows coated with polydimethylsiloxane, daytime reductions of up to 4.1°C below ambient are reached with a wind shield and thermal insulation, while a window on a real building in an urban canyon, operating without either, remains 1.2°C–1.4°C below ambient. The BSDAR bridging system preserves daylight transmission, exterior visibility, and seasonal tunability. This approach extends radiative cooling from rooftops to glazed elements of vertical facades at all orientations, supporting more sustainable cooling strategies for urban buildings.

## Graphical abstract

Radiative cooling materials are mature but only work facing the sky, confining them to rooftops. Instead of developing new ones, this system bridges existing horizontal coolers to vertical windows using blades outside the glass. On an occupied urban building, windows stay up to 1.4°C below air temperature while preserving daylight, outward views, and seasonal adjustment.

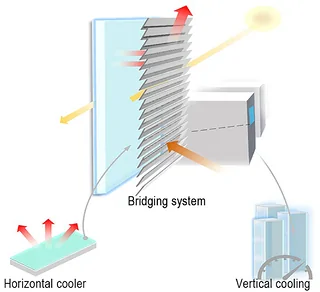

## 1 Introduction

Global cooling demand continues to rise as temperatures, populations, and incomes increase [1, 2, 3]. The urban heat island effect further intensifies local temperatures and amplifies the cooling load of buildings. Radiative cooling is emerging as a sustainable passive cooling approach for buildings [4, 5, 6]. It relies on emitting thermal radiation into the cold universe through the atmospheric transparency window [7, 8, 9, 10]. Over the past decade, radiative cooling materials have advanced rapidly and now achieve high performance, cost effectiveness, and large‐area scalability, with either highly reflective or highly transparent designs [11, 12, 13, 14, 15, 16, 17]. Most existing radiative cooling materials operate effectively on horizontal surfaces such as rooftops [18, 19, 20, 21], where they directly view the sky and can provide sub‐ambient cooling. However, these materials perform poorly on vertical surfaces such as building facades, which represent a much larger fraction of the building envelope [22, 23].

Recent studies have addressed this challenge by breaking the symmetry of thermal emission using angular–spectral co‐engineering micro‐structured geometries [1, 23, 24, 25, 26, 27, 28]. Xie et al. achieved a landmark 2.5°C sub‐ambient daytime cooling on a vertical surface by co‐engineering angular and spectral selectivity into a sawtooth grating structure [1]. Subsequent work by Mandal and colleagues [23, 24, 25], Zhao [26], Cheng et al. [27], and Kim et al. [28] extended directional emission principles to wall‐integrated and window‐integrated systems, including transparent epsilon‐near‐zero multilayers and dual‐channel spectral management, while microprism arrays provided passive seasonal solar modulation. These elegant designs demonstrate that angular–spectral co‐engineering is physically achievable on vertical surfaces, and are ideally suited to new construction where purpose‐built surface structures can be incorporated at the fabrication stage.

Inspired by these advances, we seek to complement them by addressing the existing global building stock, where new surface fabrication is not available as a design tool, and where a retrofit‐compatible solution could dramatically expand the reach of vertical radiative cooling. This motivates a complementary and distinct strategy: rather than embedding angular selectivity within the radiative cooling surface itself, as in all previously reported approaches, we decouple angular selectivity and spectral emission as independent functions, so that directional cooling becomes a retrofit capability compatible with any existing wall or window, without requiring modifications to the underlying surface. We refer to this decoupled, add‐on approach as a ‘bridging system’.

While engineering new materials for vertical radiative cooling is an important direction, this ‘bridging system’ can convert existing horizontal radiative cooling materials into effective vertical radiators. A wide range of high‐performance horizontal radiative cooling materials already exists, including multilayer photonic emitters, scalable polymer films embedded with silica spheres, porous polymers, and more recently porous ceramics [29, 30, 31, 32, 33, 34, 35, 36]. Being able to directly apply these established materials onto vertical facades would leverage previous advances and accelerate the deployment of radiative cooling in urban environments. Designing such a bridging system for real building applications introduces several requirements and challenges. The system must exhibit directional asymmetry; it must effectively channel thermal radiation from the vertical surface toward the sky while limiting radiative exchange with the surroundings. It must be scalable and easy to install. In addition, modern buildings often contain large window areas, so the system should preserve the essential functions of windows, including daylighting, and exterior visibility. Meeting these combined optical, thermal, and mechanical requirements necessitates an integrated design approach.

In this study, we develop a bridging system that converts existing horizontal radiative cooling materials into effective vertical cooling materials for building facades, and we demonstrate its application on windows. The system uses a parallel‐blind optical structure to redirect mid infrared radiation from the vertical surface toward the sky while suppressing radiation from the surroundings. It maintains natural daylight transmission and unobstructed views rather than blocking incident sunlight, providing additional design flexibility for dense urban environments. The bridging system operates for south‐, north‐, west‐, and east‐facing facades. By extending radiative cooling from rooftops to the much larger facade areas, this strategy increases the cooling capacity of individual buildings and expands the collective cooling potential across cities, contributing to mitigation of the urban heat island effect.

## 2 Results

### 2.1 Concept and Design of the Broad‐Spectral Directional Array‐Reflector (BSDAR) Bridging System

In high‐density urban areas, summertime overheating of buildings has become increasingly critical for human comfort and health. Growing populations have driven the development of dense street canyons (Figures S1 and S2), while facade areas expand rapidly in the vertical direction. As a result, overheating on large vertical facades becomes a major contributor to the cooling energy demand of modern buildings. Yet most existing radiative cooling materials are designed for horizontal surfaces and cannot operate effectively when directly applied to vertical facades. This limitation is especially pronounced in narrow urban canyons, where vertical walls have small sky view factors and predominantly view warm neighboring buildings and ground surfaces rather than the cold sky. These facades therefore receive substantial thermal radiation from surrounding buildings, the ground, and even vehicles, which severely reduces their radiative cooling potential.

**FIGURE 1:**
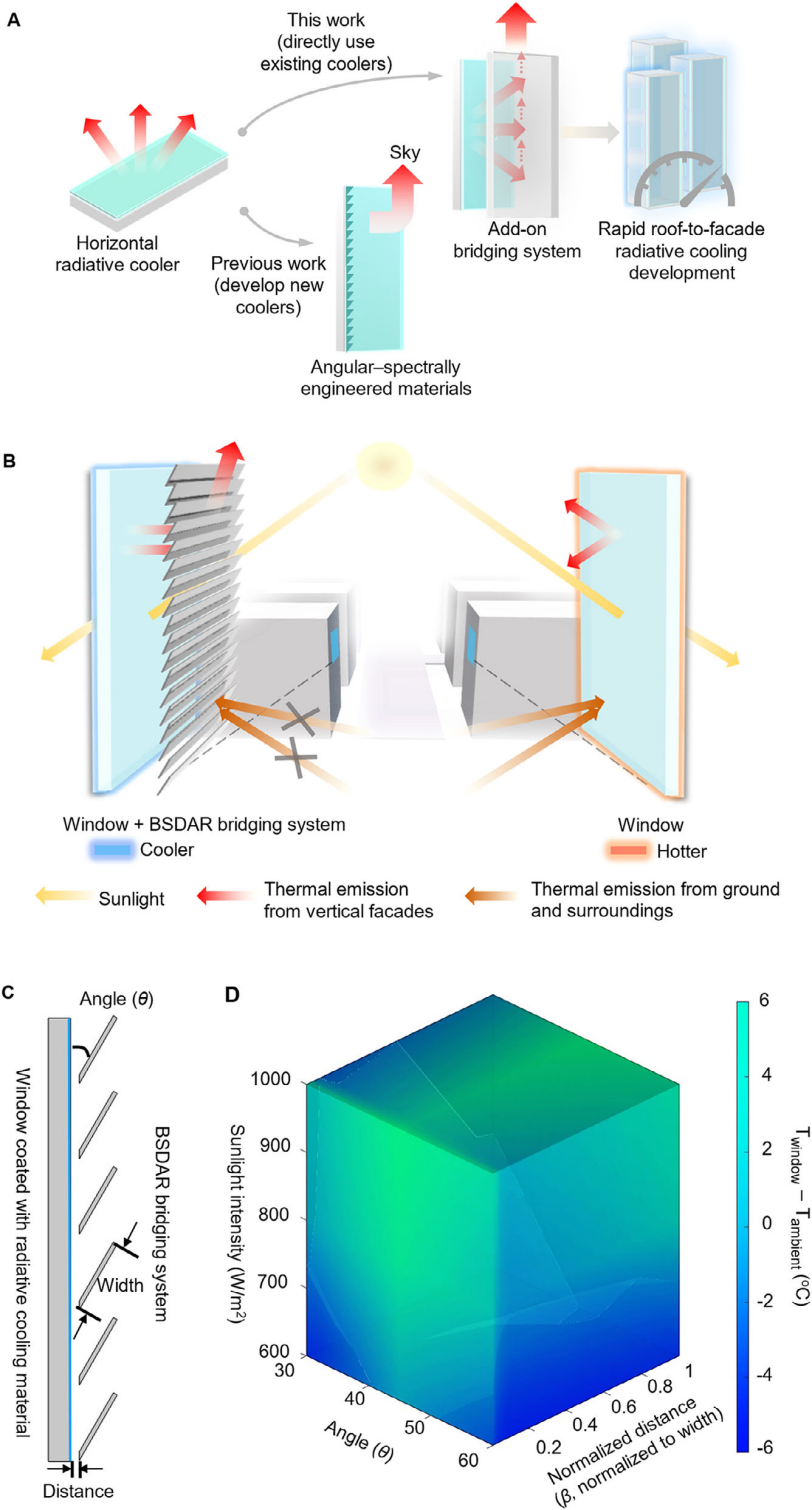
Design concept of the broad‐spectral directional array reflector (BSDAR) bridging system for radiative cooling on vertical glazing surfaces. (A) Comparison of vertical radiative cooling strategies: Previous works develop new angular‐spectrally engineered materials that emit directly toward the sky from vertical facades, whereas the proposed bridging system (this work) enables a retrofit solution that directly uses existing horizontal radiative coolers to existing building facades, thereby facilitating rapid roof‐to‐facade radiative cooling development by leveraging the vast accumulated knowledge and advances in developed horizontal radiative cooling materials. (B) Detailed working principle of the BSDAR bridging system for enabling radiative cooling on vertical facades. A vertical window coated with a common radiative cooling material, such as polydimethylsiloxane, absorbs thermal radiation from the ground and surrounding buildings while emitting limited radiation to the cold sky, resulting in ineffective cooling. After installation of the BSDAR bridging system, thermal radiation input from the ground and surroundings is suppressed, while thermal emission from the vertical surface is redirected toward the cold sky, enabling sub‐ambient cooling. The BSDAR bridging system simultaneously allows sunlight to pass through the window, preserving natural daylighting. (C) Side view of the BSDAR bridging system mounted on a window coated with the polydimethylsiloxane radiative cooling material. The key geometric parameters are the blade tilt angle *θ* relative to the vertical surface and the normalized distance *β* between the BSDAR bridging system and the window, defined as the ratio of separation distance to blade width. The vertical pitch between neighboring blades is fixed as a constant fraction of the blade width. (D) Simulated cooling performance of a vertical window equipped with the BSDAR bridging system as a function of tilt angle *θ* and normalized distance *β*. The interior shows the zero‐temperature‐difference iso‐surface. Under idealized boundary conditions, with clear‐sky radiative exchange and idealized convective coupling, the finite‐element model simulations predict sub‐ambient temperature reductions of approximately 4°C–6°C for optimized configurations with *θ* around 30° and *β* around 0.1, under solar irradiance below 750 W m^−2^. These values represent the predicted intrinsic cooling potential and are used only to compare geometries under identical assumptions in order to select the optimum parameters.

Within the facade system, windows, glazed doors, and curtain walls face an even greater challenge because they must preserve essential optical functions such as daylight transmission and exterior visibility [37, 38, 39, 40]. The combination of low sky view factors, complex multi‐source radiative environments, and optical functional requirements underscores the need for new strategies tailored specifically for vertical radiative cooling in real cities. Ideally, the solution would enable direct use of the large library of high‐performance horizontal radiative cooling materials on vertical surfaces, thereby rapidly bridging the gap between material development and practical vertical building cooling needs. We therefore propose a roof‐to‐facade blueprint (Figure 1A): an external sky‐directing add‐on system enables direct transfer of high‐performance radiative‐cooling materials designed for horizontal rooftops to vertical facades, rapidly unlocking scalable facades’ cooling across dense cities. This work therefore focuses on these glazed elements. They are also the part of the envelope where a surface treatment acts most directly: a wall is thick and carries both structural and insulating functions, so a lowered outer surface temperature reaches the interior only weakly through a large thermal resistance, whereas glazing presents a far lower resistance and in much modern construction dominates the facade area.

In this study, we realize this blueprint by developing a broad‐spectral directional array reflector (BSDAR) bridging system (Figure 1B). In the concept, a common radiative cooling material such as a polydimethylsiloxane (PDMS) coating is first applied directly to the glazed elements of vertical facade. The glazing facade is then covered externally by the BSDAR bridging system, aiming at achieving sub‐ambient radiative cooling under vertical orientation. The BSDAR bridging system consists of parallel‐blind array reflectors with ultra‐high reflectance across a broad spectral range from the solar band (0.3–2.5 µm) to the mid infrared (8–13 µm). The parallel‐blind array reflectors are installed at an adjustable tilt angle.

With this system, we aim to enable radiative cooling materials originally designed for horizontal surfaces to function effectively on vertical facades through three key optical actions: (i) directing mid infrared thermal emission from the vertical surface toward the high‐angle cold sky, while rejecting mid infrared radiation from low‐angle buildings and ground surfaces; (ii) guiding sunlight and external views into the interior to preserve the fundamental window functions; (iii) maintaining angular selectivity even as facade area increases.

In real city environments, two potential challenges must be addressed. First, the BSDAR bridging system may trap part of the incident sunlight, adding unwanted solar heating; second, thermal radiation from an opposing facade at high angles may also be trapped. To resolve these issues, we develop a radiative transfer model that includes the ground, the opposite facade, the sky dome, and solar irradiation (Text S1 and Figure S3). Using this model, we optimize two key parameters of the BSDAR bridging system, shown in Figure 1C, the reflector tilt angle *θ* and the normalized distance *β* (the reflector–window distance normalized by the reflector unit's width).

Figure 1C illustrates the model structure of a window mounted with the BSDAR bridging system for a representative configuration. We perform a full parameter sweep across *θ* and *β* under different solar irradiance levels and plot the resulting cooling performance as the temperature difference between the vertical surface and the ambient air in a 3D map (Figure 1D). Regions below the zero‐temperature‐difference iso‐surface correspond to sub‐ambient cooling. The simulations predict a minimum window temperature reduction of up to approximately 6°C below ambient.

The BSDAR bridging system performs best when *θ* is 30° and *β* is 0.1. Under these conditions, it consistently enables sub‐ambient cooling across solar irradiance levels from 600 to 1000 W m^−2^, which represent typical clear‐sky conditions. The poorest performance occurs near *θ* of 45°, where radiative heat from the opposite facade is strongly trapped. When the BSDAR bridging system is positioned too close to the vertical window, corresponding to a small *β*, radiative and conductive heat exchange between the BSDAR bridging system and the window increases, which degrades the cooling performance. When the BSDAR bridging system is positioned too far from the window, corresponding to a large *β*, a fraction of the thermal radiation emitted by the window escapes through the gap without being redirected toward the sky. Optimal cooling performance is achieved at *β* of 0.1. The same behavior is obtained with two other reported emitters, P(VdF‐HFP)HP and BaSO_4_‐acrylic paint [11, 41], under identical *θ* and *β*, indicating that the bridging function is set by the array geometry rather than by the choice of emitter (Figure S4).

### 2.2 Construction of BSDAR Bridging Systems and Testing Cooling on Building Windows

To translate the above concept into a practical solution for building facades, particularly for window systems, we fabricate BSDAR bridging systems and test them on vertical window glass panels coated with a common radiative cooling material.

**FIGURE 2:**
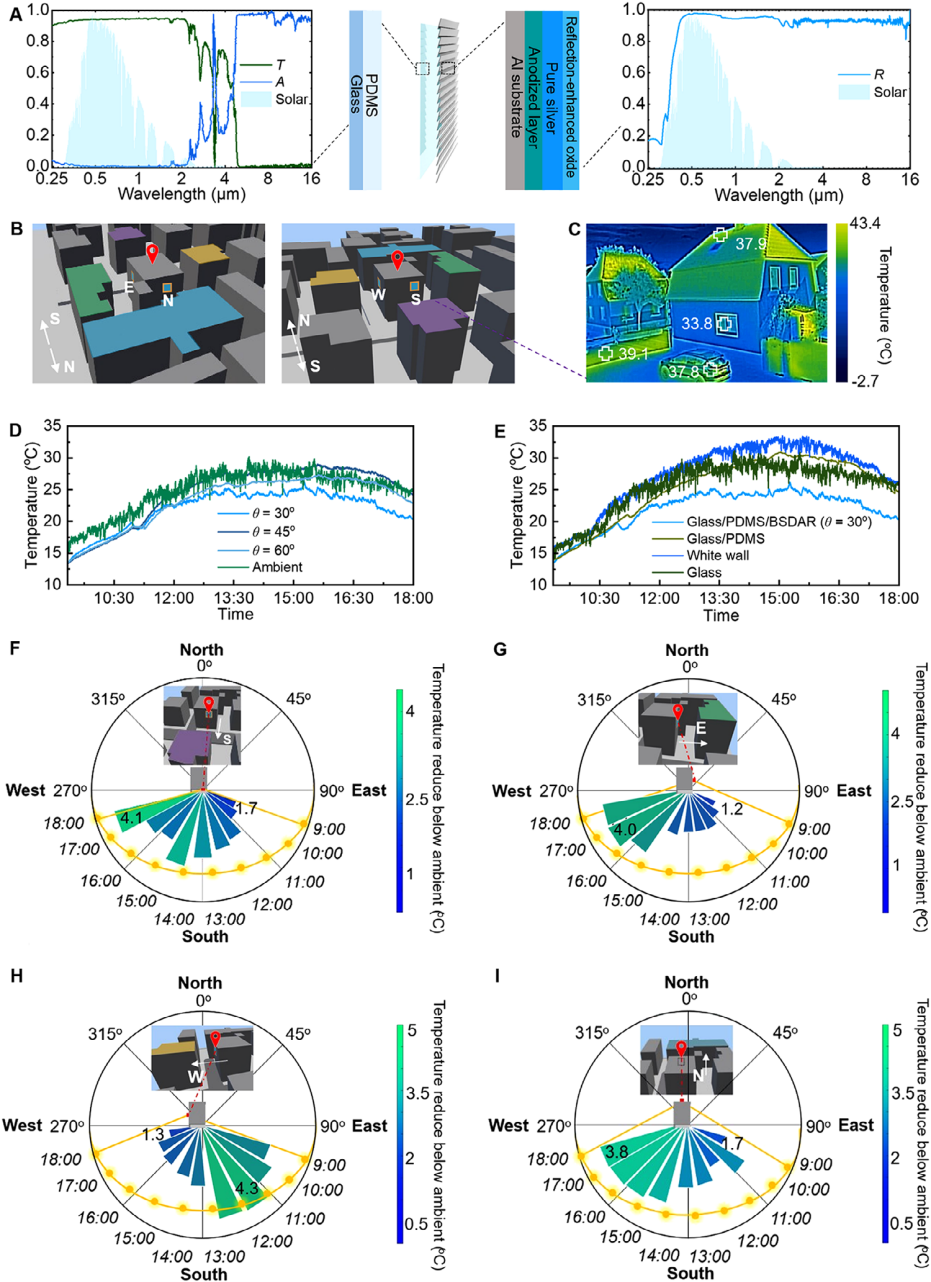
Performance of the BSDAR bridging system for radiative cooling of vertical windows. (A) Construction of the BSDAR bridging system on a vertical window and the optical properties. The window is coated with a 75‐µm‐thick polydimethylsiloxane layer, a common radiative cooling material for horizontal surfaces, which maintains high solar transmittance (0.3–2.5 µm) and high mid infrared emissivity within the atmospheric window (8–13 µm). The BSDAR bridging system consists of parallel multi‐layer reflection‐enhanced reflectors (MLRE) that provide high reflectance across both the solar and mid infrared spectral ranges. (B) Building layout and window locations used for outdoor testing. Experiments are performed on four windows of the central building, facing east, south, west, and north. The surrounding urban environment is modeled to match the real setting, with different colors indicating neighboring buildings of different orientations. The central marker identifies the tested building. (C) Infrared thermal image of the environment surrounding the south‐facing facade during testing, illustrating the complex radiative heat sources typical of dense urban canyons. (D) Cooling performance of the BSDAR bridging system on the south‐facing window under sunny conditions for tilt angles *θ* of 30°, 45°, and 60°. The configuration with *θ* of 30° provides the strongest cooling, achieving a maximum sub‐ambient temperature reduction of 4.1°C and a daytime average reduction of 2.9°C below ambient. (E) Temperature comparison between a window equipped with the BSDAR bridging system, a conventional window, and a white‐painted wall, showing that the BSDAR‐equipped window is 8.2°C cooler than the white wall and 4.7°C cooler than the conventional window. (F–I) Cooling performance of the BSDAR bridging system on windows facing south, east, west, and north, respectively, demonstrating consistent sub‐ambient cooling across all orientations under real outdoor conditions.

We coat PDMS, a widely used radiative cooling material [4, 9, 21, 34, 35, 40], onto vertical window glass. The left images in Figure 2A show the optical properties and schematic structure illustration of the coated glass. The PDMS layer has a thickness of approximately 75 µm, preserves the broadband visible transparency of the underlying glass, and significantly enhances mid infrared emission within the 8–13 µm atmospheric window, with emissivity above 94%. We use multi‐layer reflection‐enhanced (MLRE) reflector materials to construct the BSDAR bridging systems, enabling high reflectance across the visible and mid infrared ranges. The right images in Figure 2A show the MLRE coating structure and its optical properties. From bottom to top within MLRE, the structure consists of a 300 µm aluminum substrate, a 5 µm anodized bonding layer, a 56 µm silver coating as the primary reflector, and a 0.78 µm TiO_2_ coating that enhances reflectance and provides oxidation protection of silver. The full structure is provided in Figure S4. The high‐purity silver layer ensures excellent reflectivity across the visible and mid infrared bands, while the TiO_2_ overlayer (high refractive index) enhances reflectance through thin‐film interference effects [42, 43]. The MLRE reflector exhibits intrinsic reflectance above 95% across the broad spectral range relevant to both solar radiation and mid infrared thermal emission. With this high reflectivity, the BSDAR bridging system can simultaneously manage the solar band for daylighting and the mid infrared band for directional thermal control.

We test the cooling performance of the vertical window equipped with the BSDAR bridging system under calorimetric conditions on a building located along an urban canyon street. Figure 2B shows the testing environment, where experiments are performed on four windows facing east, south, west, and north, as indicated by the red marker. Specific window positions and building photographs are provided in Figure S5. The average spacing between the test windows and surrounding facades is approximately 5.2 m. Details of the experimental setup are provided in the Materials and Methods section.

Figure 2C shows infrared imaging of the surroundings near the south‐facing window, illustrating the complex radiative environment dominated by nearby building facades, street pavement, vehicles, pedestrians, and vegetation, with peak temperatures reaching up to 43°C.

Cooling the south‐facing window proves most challenging due to strong environmental heat and direct solar radiation. We first evaluate BSDAR bridging systems with tilt angles *θ* of 30°, 45°, and 60° on the south‐facing window under sunny conditions. The temperature curves in Figure 2D show that the BSDAR bridging system with a tilt angle of 30° achieves the best performance, consistent with simulation predictions, reaching a maximum sub‐ambient cooling of 4.1°C during 17:00 to 18:00 and an average sub‐ambient cooling of 2.9°C during the daytime (10:00 to 18:00). This performance is among the strongest reported for vertical radiative cooling [1, 2, 24, 25, 26]. As shown in Figure 2E, the PDMS‐coated window with the optimal BSDAR bridging system is 8.2°C cooler than a traditional white wall and 4.7°C cooler than bare window glass. The temperature difference between the coated window with and without the BSDAR bridging system reaches 4.1°C, confirming that the BSDAR bridging system effectively converts a common horizontal radiative cooling material into a vertical radiative cooling system for real building facades.

The solar position strongly influences the cooling performance of vertical windows installed with the BSDAR bridging system. Sunlight provides direct heating to the target window and also heats the surroundings, which affects radiative exchange. Figure 2F shows the hourly sub‐ambient cooling performance of the south‐facing window on a sunny day and includes the solar trajectory for reference. The top portion of Figure 2F shows the window location and surrounding environment, while the bottom portion shows the evolution of solar position. Fan‐shaped bars represent hourly average sub‐ambient cooling. The BSDAR bridging system achieves maximum sub‐ambient cooling of 4.1°C from 17:00 to 18:00 and average cooling of 2.9°C from 09:00 to 18:00, even though the south‐facing window receives solar exposure throughout the day. The improved afternoon performance is associated with enhanced skyward radiative cooling as relative humidity decreases. The transient dip around 15:00–16:00 likely reflects peak parasitic thermal infrared loading from the warmed opposing facade and asphalt/vehicles, while cooling recovers at 17:00–18:00 as solar and urban heat inputs subside. Under partially cloudy conditions, the system maintains effective cooling, reaching a maximum sub‐ambient temperature reduction of 3.2°C during 09:00 to 18:00 (Figure S6). During nighttime, the system achieves a high average 3.3°C sub‐ambient cooling (Figure S7). These results demonstrate effective BSDAR's operation under sunny and cloudy conditions and during both daytime and nighttime.

We then test the BSDAR bridging system on east‐, west‐, and north‐facing windows under sunny conditions (Figure 2F–I). The east‐facing window achieves a maximum sub‐ambient cooling of 4.0°C from 17:00 to 18:00 and an average of 2.6°C from 09:00 to 18:00. The cooling performance is stronger in the afternoon due to lower humidity and the absence of direct solar heating. The west‐facing window achieves a maximum cooling of 4.3°C from 11:00 to 12:00 and averages 2.9°C from 09:00 to 18:00. The west‐facing performance is significantly better in the morning since the window receives stronger solar heating in the afternoon. The north‐facing window achieves a maximum cooling of 3.8°C from 17:00 to 18:00 and an average of 3.0°C from 09:00 to 18:00. Although the north‐facing facade does not receive direct sunlight, solar heating of the opposite facade increases its thermal emission toward the window. Detailed temperature curves and infrared images of the surroundings are provided in Figures S8–S13. Weather conditions including solar irradiance, humidity, and wind speed during the test periods are shown in Figure S14. Figure S15 shows infrared images of the window with the BSDAR bridging system, clearly demonstrating sub‐ambient cooling. All these results further validate that the BSDAR bridging system can convert horizontal radiative cooling materials into effective vertical radiative cooling solutions for building facades, bridging advanced material technologies with real‐world urban applications.

### 2.3 Upscaling of the BSDAR Bridging System and Testing Cooling, Daylighting on a City Building

**FIGURE 3:**
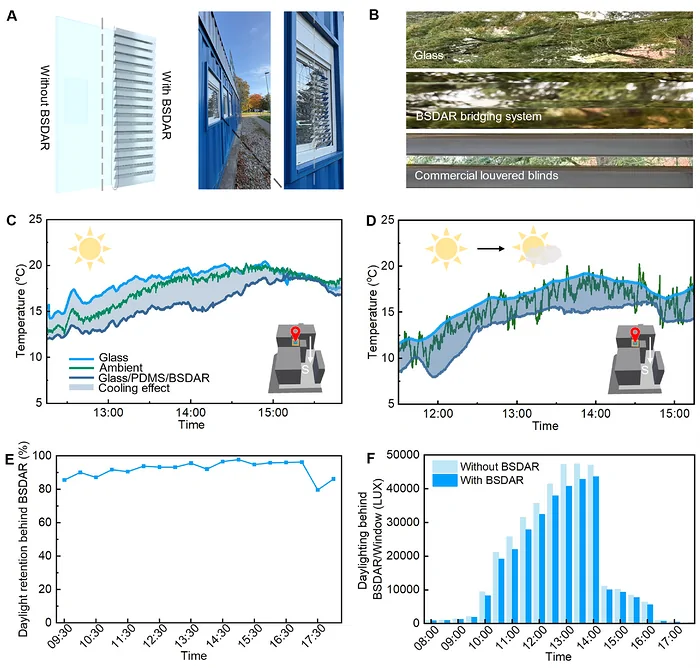
Demonstration of a size‐upscaled BSDAR bridging system for cooling and daylighting. (A) Structural schematic (left) and photographs of the installed system (middle, building‐scale view; right, enlarged window view). Dashed outlines indicate the bare window and the window equipped with the BSDAR bridging system. (B) Outdoor views through a bare glass window, a window equipped with the BSDAR bridging system, and a window fitted with a commercial louver blind. The BSDAR bridging system preserves a continuous exterior view, enabled by a highly reflective, periscope‐like optical pathway formed by the MLRE reflectors. (C) Cooling performance of a south‐facing window equipped with the BSDAR bridging system under sunny conditions, compared with a bare window reference. The large‐scale BSDAR bridging system achieves a maximum sub‐ambient temperature reduction of 3.7°C and an average daytime reduction of 2.5°C relative to the bare window. (D) Cooling performance of the south‐facing window equipped with the BSDAR bridging system under sunny‐to‐cloudy conditions, compared with the bare window reference. (E) Light transmission performance of the BSDAR bridging system, expressed as the ratio of transmitted light intensity with and without the BSDAR bridging system on a west‐facing window. The system shows minimal impact on daylight admission, with an average light retention of 92%. This position characterizes the light immediately behind the glazing rather than its distribution across the room. (F) Comparison of indoor light flux with and without the BSDAR bridging system on a south‐facing window, showing negligible degradation of indoor daylighting after installation.

Scaling the BSDAR bridging system from a prototype to a large‐format installation is essential for practical deployment on real buildings. Upscaling introduces additional complexity, such as increased absorption of environmental heat from nearby buildings and ground surfaces, and therefore requires further investigation [3, 5, 22]. For large‐scale implementation, the BSDAR bridging system is designed as a louvered blind‐like structure that can be mounted externally onto vertical windows. This design allows the tilt angle of the MLRE reflector units within the BSDAR bridging system to be adjusted without affecting the fundamental functions of windows, including daylighting and exterior viewing. Figure 3A shows the scaled‐up BSDAR bridging system mounted on a window.

Figure 3B compares the visual experience from inside the building when using the BSDAR bridging system, a bare window, and a commercial louvered blind system. The BSDAR bridging system preserves a continuous exterior view and maintains daylighting, whereas the commercial louvered blind system partially obstructs the outdoor view. This difference arises from the highly reflective double‐sided MLRE reflector units in the BSDAR bridging system, which create a periscope‐like optical effect that reflects light into the room while retaining detailed visibility of the surroundings [44, 45]. Figure S16A further illustrates the building environment used for these comparison tests and shows how the tilt angle of the BSDAR bridging system can be conveniently adjusted through a woven cord. This mechanism enables personalized daylighting control and thermal management based on weather conditions and allows the system to achieve full transparency when desired.

To evaluate the cooling performance of the large‐scale BSDAR bridging system, we conduct side‐by‐side tests on building windows located along an urban canyon residential street, under the same calorimetric conditions and mounted on a real building window via a woven cord mechanism (Figure S16B; Materials and Methods). Figure 3C,D present the cooling performance under sunny and sunny‐to‐cloudy conditions, respectively. In both weather scenarios, the BSDAR bridging system cools the vertical window below ambient temperature. Under clear skies (Figure 3C), the large‐scale BSDAR bridging system achieves a maximum sub‐ambient reduction of 3.7°C and an average reduction of 2.5°C during the daytime compared to the bare window without the BSDAR bridging system. Under sunny‐to‐cloudy conditions (Figure 3D), the system achieves a maximum temperature decrease of 4.0°C below ambient and an average daytime reduction of 2.1°C relative to the bare window without the BSDAR bridging system. Even under fully cloudy conditions (Figure S17), the large‐scale BSDAR bridging system maintains effective cooling. Weather conditions, including humidity, solar irradiance, and wind speed, are provided in Figure S18. These results show that the BSDAR bridging system performs effectively in complex urban environments.

We further evaluate the sunlight transmission performance of the BSDAR bridging system. The overall light transmission ratio, defined as the ratio of light intensity measured with the BSDAR bridging system to that without the system, reaches 92% (Figure 3E), characterizing the transmission of the array itself and indicating minimal impact on window daylighting. To further assess indoor lighting conditions, we compare the indoor light flux with and without the BSDAR bridging system on a south‐facing window (Figure 3F). The results show negligible degradation of indoor daylighting after installation. This performance is attributed to the high reflectivity of the double‐sided MLRE blades (schematic illustration in the inset) and the carefully optimized array tilt angle and normalized spacing of the BSDAR bridging system. Thermal imaging of a room fitted with a large‐format BSDAR array shows no localized hot spots on the desk, floor or wall surfaces (Figure S19). Radiance simulations for the same location show that BSDAR gives the lowest Daylight Glare Probability (DGP) among a bare window, a commercial exterior venetian blind of identical slat geometry and BSDAR in all conditions examined, falling from 0.44 for the bare window to 0.38 at solar noon, where the bare case approaches the intolerable threshold (Figures S20 and S21). BSDAR's specular redirection does not concentrate light indoors: the near‐window peak illuminance at solar noon is 13,947 lux with BSDAR against 13,729 lux for the commercial exterior venetian blind, both about 35% below the bare window, while mean work‐plane illuminance is comparable between the two devices, the commercial blinds being the more uniform with depth (Figure S22). Two‐probe illuminance data in field‐test at 0.9 and 2.35 m from the south‐facing window in a real building reproduce this ordering near the window (Figure S23). BSDAR therefore delivers a comparable amount of daylight with less glare.

To bridge the gap between calorimetric performance limits and real deployment conditions, we removed all convective shielding and test frames and measured surface temperatures on real building windows fitted externally with the large‐format BSDAR, under both clear‐sky and cloudy conditions, comparing south‐ and north‐facing facades with and without PDMS coating. Despite the absence of any wind barrier or thermal isolation, sub‐ambient surface temperatures were consistently achieved across all tested configurations and sky conditions. Under clear skies, north‐facing windows attained a daytime‐average sub‐ambient reduction of 1.0°C and a peak of 1.4°C (Figure S24); south‐facing windows reached an average of 0.5°C and a peak of 1.2°C (Figures S25 and S26), with sub‐ambient margins maintained under cloudy conditions on both orientations (Figures S27–S29). The larger margin on north‐facing facades reflects reduced solar heat load in the absence of direct solar gain. The smaller absolute reductions relative to calorimetric measurements reflect dominant convective exchange at unshielded surfaces; nevertheless, sustaining positive sub‐ambient margins under fully uncontrolled urban conditions confirms that the BSDAR delivers meaningful cooling benefit directly applicable to the existing building stock. To confirm that sub‐ambient operation is retained under the hot weather that drives cooling demand, the enlarged BSDAR was also measured on a south‐facing building window at ambient temperatures above 30°C, without any wind shield or thermal insulation. The window surface remained on average 0.9°C below ambient over the daytime period (Figure S30), a larger depression than under cooler conditions, since the drier air provides a greater sky depression (Figure S31).

### 2.4 Energy Savings and Season Adjustability Enabled by BSDAR Bridging Systems for Buildings

**FIGURE 4:**
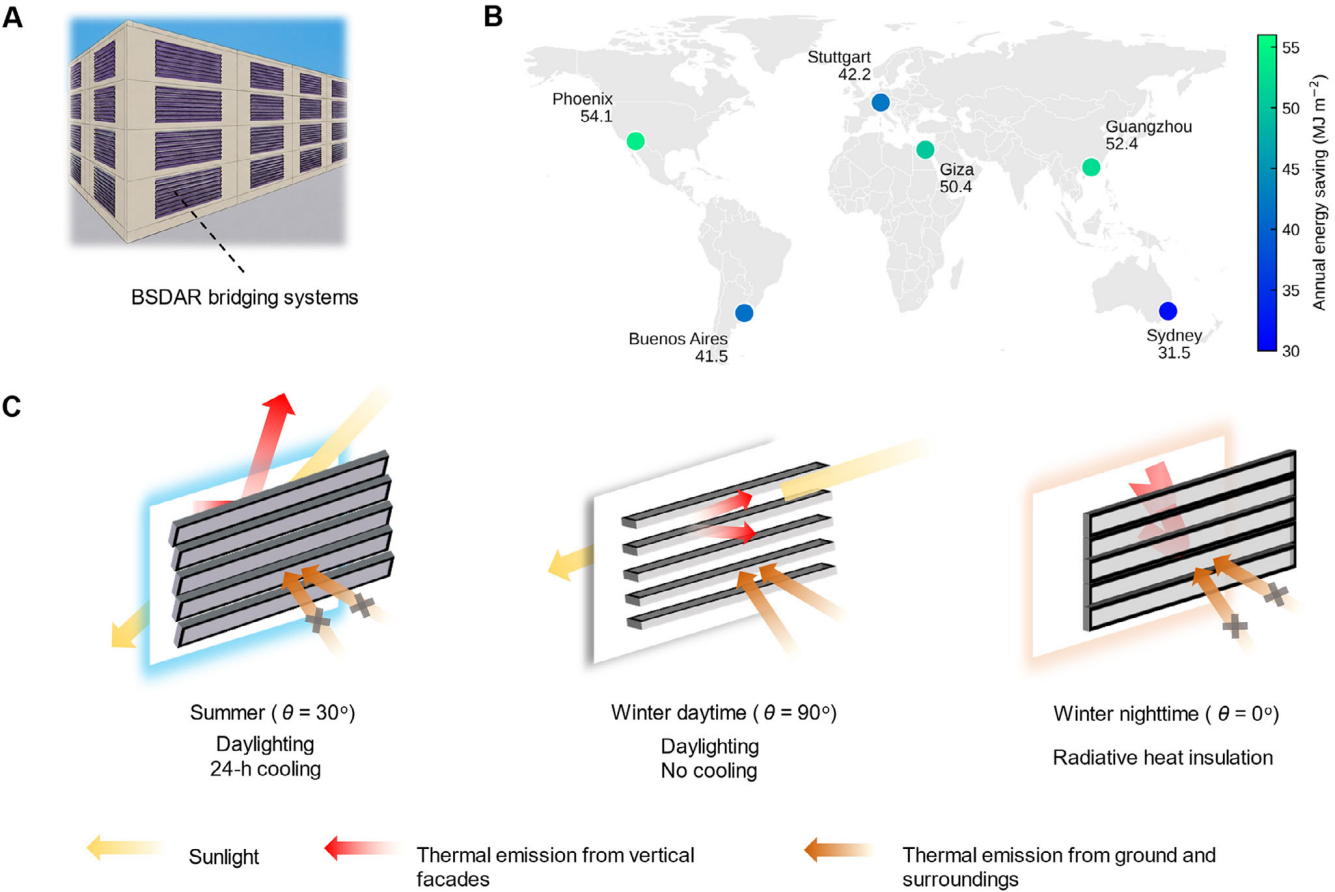
Energy savings enabled by the BSDAR bridging system with seasonally adjustable thermal management. (A) Building geometry used for energy modeling. In the simulations, facade reflector arrays on windows are assigned the same optical and thermal properties as the BSDAR reflectors. (B) Global distribution of cumulative cooling energy savings enabled by the BSDAR bridging system, normalized by floor area, for six cities on six continents. The array is mounted on the windows only. Cooling savings reach 53.5 MJ m^−^^2^ and heating savings 17.0 MJ m^−^^2^, giving an annual total of 31.5 to 54.1 MJ m^−^^2^. (C) Seasonally adjustable thermal management enabled by the BSDAR bridging system. In summer, the system (*θ* = 30°) enhances cooling by directing thermal radiation from the facade toward the cold sky while suppressing environmental heat input and allowing high‐angle sunlight for daylighting. In winter, the system (*θ* = 90°) suppresses cooling and permits low‐angle sunlight during daytime, while the system (*θ* = 0°) minimizing radiative heat exchange with the exterior at night.

Building upon the demonstrated cooling effect of the BSDAR bridging system on real city buildings, we further evaluate its global potential for energy savings and CO_2_ emission reduction. To ground this analysis in a realistic architectural context, we identify a real building whose windows are equipped with a louvered blind‐like structure similar to the BSDAR bridging system (Figure S32). Using this building as a representative model (Figure 4A), we construct a simulation framework in which all windows are fitted with the BSDAR bridging system (Figure S33). To assess the energy‐saving potential, we use EnergyPlus in combination with Open Studio to simulate building energy consumption [46]. The simulations incorporate solar radiation and meteorological conditions characteristic of various regions around the world (https://energyplus.net/weather). The optical properties of the BSDAR structure are taken from the spectral measurements in Figure 2, and the directional radiative exchange is obtained by ray tracing on the as‐built array geometry. The measured sub‐ambient depressions are used as an independent check that the simulated facade temperatures are not overstated. The full simulation workflow is summarized in Figures S34 and S35. By comparing the monthly cooling energy consumption with and without the BSDAR bridging system, we quantify the resulting energy savings. This comparison provides insight into the global potential of the BSDAR bridging system for reducing cooling energy demand.

We select six cities on six continents, spanning tropical, subtropical, desert, temperate and oceanic climates: Guangzhou, Stuttgart, Phoenix, Giza, Buenos Aires and Sydney. The seasonal energy savings with the BSDAR bridging system are presented in Figure 4B, with the array mounted on the windows only (calculation details in Text S2). In summer the addition of the BSDAR bridging system results in substantial cooling energy savings with the array held at 30°. Relative to a bare window, cooling demand falls by 20.5% in Guangzhou, 36.7% in Stuttgart, 22.3% in Phoenix, 24.4% in Giza, 31.5% in Buenos Aires and 31.4% in Sydney, corresponding to 25.2–53.5 MJ m^−^^2^ of building floor area. The largest absolute savings occur in the hot climates of Phoenix and Guangzhou, consistent with their high cooling demand. In winter the array is reconfigured, being opened to 90° during the day to admit low‐angle sunlight and closed at night to suppress radiative loss, a strategy described in detail below. Heating demand in winter also falls, by 1.1% to 9.9% or 0.6 to 17.0 MJ m^−^^2^. The system therefore does not trade a summer benefit against a winter penalty: the annual total falls by 15.6% to 23.2%, or 31.5 to 54.1 MJ m^−^^2^, in every city examined. Holding the array at 30° throughout the year instead angle adjusting will raise winter heating demand by 12% to 95%, so the seasonal adjustment is a requirement for year‐round benefit (Figure S36). With all conditioning disabled, the free‐floating indoor air temperature falls by 2.6–3.4°C at the summer peak and by 1.1–2.3°C on seasonal average, and in winter remains above that of a bare window in every hour of the coldest representative week (Figures S37 and S38). The BSDAR bridging system demonstrates strong performance in cooling energy savings during summer and not increase, placing it among the leading reported systems for vertical radiative cooling [4, 18, 37, 47, 48, 49, 50]. Based on the building energy simulation results, we also quantify the associated carbon emission reduction. This is done by coupling the energy savings in Figure 4B with region‐specific grid electricity emission factors (kg CO_2_ per kWh) [18, 50, 51, 52] (https://ourworldindata.org/co2‐emissions). Figure S39 maps the resulting CO_2_ reduction across the six cities. Emissions fall by 1.19 to 2.55 kg m^−^^2^ of floor area per year. Summed over the six buildings this amounts to 56 t CO_2_ per year, or 2.8 kt for a community of fifty such buildings in each city.

Retrofit deployment also requires the BSDAR to remain stable outdoors. To probe hygrothermal and photolytic degradation, BSDAR were held at 85°C and 85% relative humidity under one‐sun irradiance for 100 h in the climate chamber (Figure S40A), following the damp‐heat stress condition of IEC 61215‐2. The Planck‐weighted reflectance in the atmospheric window (8–13 µm), which governs the bridging function, decreased by 0.22% and the AM1.5G‐weighted solar reflectance by 0.71%, indicating no appreciable degradation of the optical function. Ten days of outdoor dust exposure reduced the same two quantities by 1.17% and 1.65%, respectively, and a single rinse equivalent to one rainfall event fully restored the surface, with no deposit remaining visible (Figure S40B). Extrapolating the measured degradation to solar reflectance losses of 4–12% attenuates the sky‐coupled fraction of the window's emission by 2.3–6.7%, while the summer cooling demand increase by less than 1.0% in all three typical cities (Figure S41).

The seasonal reconfiguration in BSDAR system referred to above is achieved by easily adjustable slat angles (Figure 4C), addressing the limitations of conventional radiative coolers by providing winter tunability [19, 53]. In summer mode, with the array tilted to 30°, the BSDAR bridging system enhances cooling by redirecting thermal radiation from the facade toward the cold sky, suppressing heat input from the surroundings, while allowing high‐angle summer sunlight to enter for daylighting. Ray tracing of the glazing's emission through the as‐built geometry gives 43.5% reaching the sky against 6.2% reaching the ground, with the fraction returning to the glazing carrying no net heat (Text S3). During winter daytime, the array is rotated to approximately 90°, which suppresses radiative cooling and permits low‐angle solar radiation to enter the interior. During winter nighttime, the array is closed to 0°, forming a closed configuration in which the BSDAR bridging system reflects thermal radiation from the facade back toward the interior and further reduces radiative heat exchange with the exterior by reflecting cold radiation from the ground away (see Figure S42 for experimental results). This closed configuration reduces heat loss through the window by 66%, against 47% for a commercial blind of identical geometry; the difference arises entirely from the infrared reflectance of the slat surface (Text S4). Because the solar trajectory is set by latitude and declination, we further evaluated whether the summer angle remains acceptable across both. At the latitude of our field measurements, Karlsruhe area (49°N), the fixed 30° configuration satisfies the net‐cooling and daylight criteria throughout the hot season required cooling, and acceptability increases with latitude; at lower latitudes the higher solar altitude reduces daylight admission below the criterion while net cooling is still achieved, so daylight rather than cooling capability sets the limit there (Figures S43 and S44).

Building on the results above, the BSDAR bridging system achieves sub‐ambient glazing facade cooling at levels comparable to leading vertical radiative cooling technologies, as shown in Table S1. Beyond its cooling performance, the system offers several practical advantages: it preserves daylighting and exterior visibility; it is adjustable, rollable and closable, enabling flexible energy management and seasonally reconfigurable thermal management; and it is portable, storable and easily transportable, supporting large‐scale deployment. It also carries no unusual installation burden. Its mechanical configuration is that of a conventional external venetian blind, differing only in the optical treatment of the slat surface, so it can adopt the support design and certified wind‐resistance classes of an established building product and can be retracted under extreme wind. It mounts externally without modification of the glazing or frame, leaving the window opening unobstructed, whereas low‐emissivity glazing requires replacement of the insulating glass unit. Its hydrophobic surface is restored by a single rainfall event, so rainfall alone maintains optical performance in temperate climates. These features make the BSDAR bridging system suitable for diverse glazed elements of vertical facades, including windows, glazed doors and curtain walls. Extension to opaque surfaces would require the emitter to be applied to rough, porous substrates; PDMS applied to a rough wall surface remained adherent over ten days’ exposure (Figure S45). Among commercially available alternatives, neither exterior venetian blinds nor low‐emissivity glazing provides this radiative pathway (Table S2 and Text S5). By converting advanced horizontal radiative cooling materials into functional coolers for vertical facades, the BSDAR bridging system provides a practical pathway from material innovation to functional solutions for building‐scale cooling, enabling scalable, year‐round energy savings and carbon reduction.

## 3 Conclusion

This study presents a practical and directional‐redirection strategy to extend radiative cooling from horizontal surfaces to vertical building facades through the BSDAR bridging system. By combining broad‐spectral reflectivity with directional thermal management, the BSDAR bridging system enables common radiative cooling materials, originally designed for horizontal surfaces, to operate effectively on vertical windows and walls in dense urban environments. The BSDAR bridging system maintains essential facade functions, including daylighting and exterior visibility, while delivering sub‐ambient cooling under sunny, cloudy, and nighttime conditions across all window orientations.

Large‐scale outdoor tests confirm that the BSDAR bridging system provides consistent cooling in real urban canyon settings. Energy modeling across global climate zones further shows that integrating the BSDAR bridging system into building facades can substantially reduce cooling energy demand and associated CO_2_ emissions. The system also offers seasonal adaptability, as it can manage both thermal and daylighting by easily adjusting angles to improve summer cooling and winter insulation performance.

The combined advantages of directional radiative control, compatibility with existing materials, optical functionality, mechanical adjustability, and ease of installation make the BSDAR bridging system a practical solution for urban buildings. By bridging well‐developed horizontal radiative cooling materials to vertical surfaces, this work provides a direct pathway toward expanding radiative cooling across the full building envelope, enhancing urban thermal management, and supporting sustainable cooling in cities worldwide.

## 4 Methods

### 4.1 Material Sources

Polydimethylsiloxane (PDMS) solution is obtained from MOMENTIVE (RTV615A+B kit, material no. 62520, batch no. 24FWFA026). High‐transparency glass is purchased from Hongfeng Quartz Co., Ltd. The MLRE reflector blades of the BSDAR bridging system are custom‐made by ALANOD using MIRO high‐reflective‐95 material. The 3D printed polylactide (PLA) filament is purchased from PRUSA (PRUSA MINI white PLA). The BSDAR ladder cord is purchased from JALOUSIENE (flat slat 80 mm, grey). Transparent acrylic glass panels are purchased from E&H Design. Transparent polyethylene film is obtained from REWE (no. 2105216). Adhesive glue for bonding the double MLRE reflectors and assembling 3D‐printed components is purchased from Nayrmaer (PLA glue). The insulation material, consisting of air‐filled heat‐insulating raised foam between double‐sided reflective aluminum foil, is obtained from PRODUKTIV HANDEL (Product No. 534460‐V) and applied to the exterior sides of the small‐scale testing device. Single‐sided ethylene–vinyl acetate (EVA) sealing tape, used for sealing the testing device and preventing wind interference, is purchased from COUMENO.

### 4.2 Preparation of PDMS and Assembly of BSDAR Bridging System

PDMS precursors A and B in the RTV615A+B kit are mixed at a 10:1 weight ratio and manually stirred for 10 min. The mixture is placed in a vacuum oven (VACUTHERM 6050 M, Thermo Scientific Thermo Electron LED GmbH) for 30 to 40 min, with repeated vacuum cycles at room temperature until all bubbles are removed. The PDMS mixture is then coated onto glass using an automatic film applicator (ZAA 2300, ZEHNTNER Testing Instruments) and an adjustable blade (ZUA 2000.100, ZEHNTNER Testing Instruments) at 1 mm/s. The coating thickness is set to 75 µm and is confirmed using scanning electron microscopy (SEM). After coating, the PDMS‐coated glass is placed on a hot plate (IKA C‐MAG HS7 digital, IKA) at 100°C for 20 min to fully cure the film. For large‐scale applications, PDMS can also be coated manually. Tesa tape of known thickness (tesa Perfect+, part number 56536) is applied around the glass edges to control thickness. PDMS is then spread evenly with a flat bar and left at room temperature for 5 h to cure. The MLRE reflectors of the BSDAR bridging system are dimensionally cut using a foil cutting machine. For small‐scale tests, MLRE reflector blades are cut to widths of 1.2 cm; for large‐scale tests, MLRE reflector blades are cut to 8 cm. PLA adhesive glue is used to bond two MLRE reflector pieces into a double‐sided high‐reflective blade. In small‐scale testing, the double‐sided MLRE reflector blades are fixed onto a 3D‐printed frame to control angle and spacing (Figure S46). In large‐scale testing, the MLRE reflector blades are integrated into ladder cords, with angle and spacing controlled by a rope mechanism (Figure 3).

### 4.3 Outdoor Testing on Real City Building Windows

In the small‐scale test, the BSDAR bridging system is mounted on a frame and aligned in parallel (Figure S47). Comparison samples including bare glass and PDMS‐coated glass are fixed within the 3D‐printed frame). A polyethylene film is placed in front of each glass sample (10 cm × 10 cm) to reduce convection effects. A 2 mm thick acrylic plate (10 cm × 10 cm) is placed behind each sample. The device's four sides are insulated with air‐filled raised foam lined with reflective aluminum foil. The edges are sealed with window‐grade EVA tape to prevent wind interference. Ambient temperature is measured using a 3D‐printed white temperature chamber modeled after commercial weather station housings, positioned in front of the wooden testing frame. Solar irradiance is measured using a thermoelectric sensor (spectral range 300 to 3000 nm), oriented upward. The temperature of a reference white wall is measured by attaching thermocouples to the wall surface with copper tape. Each sample uses a Capson ultrafine T‐type thermocouple placed at its center. A miniature reflective shading plate prevents direct sunlight from heating the sensor wire. Thermocouple outputs are recorded using a Picolog data logger. Relative humidity and wind speed are recorded using a nearby weather station. For large‐scale BSDAR bridging system tests, the thickness of the sensing frame is reduced to 2 cm to maximize contact with the window while maintaining sealing against convection (Figure S48). The sensing frame is directly attached to the building window, and all other testing procedures remain consistent with the small‐scale method. Testing is conducted in Eggenstein‐Leopoldshafen, Germany. Small‐scale tests are performed from September 18 to 21, 2025 (south, east, and west orientations) and on October 2, 2025 (north orientation). Large‐scale testing and optical imaging are conducted across October 1 to 5, 2025. A reference PDMS‐coated glass sample is also tested horizontally under identical conditions, except without a BSDAR frame.

Daylighting performance was quantified using an integrated illuminance meter (AS813, Xima) to record time‐resolved illuminance. The meter features a semicylindrical sensing head, which was mounted upright (vertical) with its curved sensing surface facing outdoors. The sensor was placed at the geometric center of the window segment (unless otherwise stated). To isolate the effect of the BSDAR bridging system, paired measurements were performed under matched outdoor lighting conditions by comparing windows with and without BSDAR. Paired measurements were performed simultaneously on adjacent reference and BSDAR‐mounted window parts. In the test of daylight retention at the window plane in Figure 3E, to evaluate whether the BSDAR structure alters the intrinsic light admission at the window surface, the light probe was placed in the cavity between the BSDAR and the window glass on a west‐facing window facade (located at Eggenstein‐Leopoldshafen, Germany). Measurements were conducted for two configurations: (i) BSDAR installed, and (ii) BSDAR removed (reference). Daylight retention was defined as the ratio of the measured illuminance with BSDAR to that without BSDAR under the same condition. In test of indoor transmitted daylight in Figure 3F, to assess perceived indoor daylighting, the light probe was placed indoors at a fixed location 5 cm from the south‐facing window plane (located at Eggenstein‐Leopoldshafen, Germany), with the sensor facing the window to capture transmitted daylight reaching the near‐window interior zone. Illuminance was recorded with the window in two configurations: (i) BSDAR installed, and (ii) BSDAR removed (reference). The comparison quantifies the influence of BSDAR on indoor daylight transmission. In the multiple probe testing, the large‐format BSDAR array was installed on the exterior of a south‐facing window of an occupied room. Interior surface temperatures were surveyed with the thermal camera at an emissivity setting of 0.95, together with matching visible photographs. Interior illuminance was recorded with two calibrated photometric probes on the room centerline at 0.90 m and 2.35 m from the window at 0.75 m height, under the three configurations, at 9:00, 12:00 and 17:00 on 25 July, 2026. The sky was clear throughout the day, so that cloud movement did not confound the comparison. At each time point the three configurations were exchanged in rapid succession and readings were taken once the probes had stabilized, ensuring that the three measurements at a given time were made under effectively identical sky conditions. For the comparison with a commercial device, BSDAR and a commercial venetian blind were installed side by side on the exterior of a real building and monitored without any wind shield or thermal insulation on 12 July, 2026. The slats of the commercial blind were detached from their original headrail and reassembled on the same support and at the same width, pitch and tilt as the BSDAR array, so that the two devices differ only in slat surface. Thermocouples were placed on the slat surfaces and on the glazing behind each device.

The small‐ and large‐scale calorimetric measurements described above were conducted under standardized conditions consistent with those existing reports, thereby enabling direct comparison and establishing an upper‐bound estimate of the radiative cooling performance. Specifically, the polyethylene (PE) windshield film was employed to suppress convective heat exchange, thereby emulating the worst‐case scenario of a calm summer day on which wind‐driven cooling is absent. The insulating frame assembly was designed to thermally decouple the sample from the surrounding indoor environment, ensuring that the measured temperature differential reflects localized radiative cooling rather than conductive or convective interactions with the building interior. The baseline for the intrinsic cooling performance of the PDMS horizontal radiative cooling layer on glass was shown in Figure S49.

In addition to these standardized evaluations, we performed supplementary measurements under real‐world building conditions, in which all framing components and the PE windshield film were entirely removed. In these experiments, thermocouples were placed directly in the cavity between the window glass and the BSDAR bridging system to capture the sub‐ambient temperature drop achieved under natural, uncontrolled outdoor conditions representative of an occupied building facade. These results, which reflect the practical cooling performance of the BSDAR system in the absence of any experimental boundary conditions, are presented in Figures S24–S31.

To follow the indoor condition immediately behind the treated glazing, a sealed enclosure was mounted on the interior surface of the window. Its opening matched the projected area of the BSDAR array on the glazing, so that the enclosure sampled the region of the window that the array covers, and it formed a cavity 5 cm deep. The enclosure was 3D‐printed in white PLA and sealed to the glass with adhesive tape along all edges. A thermocouple at the geometric center of the cavity recorded the air temperature, logged at the same interval as the surface and ambient sensors. The enclosure is a proxy for the near‐window indoor environment: it has no internal heat gain and no ventilation, so it follows the glazing more closely than a real room would, and its temperature is reported for the trend rather than as an indoor air temperature.

### 4.4 Characterization

SEM imaging for Figure S5A is performed using an Inspect F50 system, while Figure S5B is obtained using a ZEISS SUPRA 60VP at 5.00 kV. Although individual layers in the MLRE structure are not visually distinct in SEM due to deposition and inter‐diffusion, the TiO_2_ top layer is exposed through diamond knife scratching (Figure S5C). Reflection and transmission spectra are measured using a UV–vis–NIR spectrophotometer (Agilent Cary 7000) over 300 to 2500 nm, using an integrating sphere for both diffuse and direct components. Emissivity measurements are taken using a Fourier‐transform infrared spectrometer (Bruker Vertex 70) equipped with an A562 gold‐coated integrating sphere. Infrared thermal images are captured using a FLIR C5 camera with emissivity set to 0.95 and relative humidity to 50%. Images are annotated and exported using FLIR Ignite software.

### 4.5 FEA Simulations, Radiance Simulations, Energy Savings Calculations, and Visualization

The FEA simulations in Figure 1 are performed using COMSOL Multiphysics, using surface‐to‐surface radiation and solid heat transfer modules. External radiation is included in the model. Full details of model structure and parameter settings are provided in Text S1. Energy savings in Figure 4 are computed using EnergyPlus, with implementation details described in Text S2. The ray‐tracing analysis of the directional infrared transport of the array, summarized in the last paragraph of this section, is described in full in text S3 (Figures S50–S53). The global energy savings distribution map in Figure 4 is generated using MATLAB based on the Köppen climate classification map, with customized color scales and representative values.

Interior daylighting was simulated with Radiance software for a 5.0 m × 4.0 m × 2.7 m room with south‐facing window, using a TMYx weather file (a typical meteorological year file assembled from recent multi‐year observations at the nearest station) for local on 21 June at 9:00, 12:00 and 17:00. Surface reflectance were 0.8 (ceiling), 0.5 (walls) and 0.2 (floor). The BSDAR array and the reference commercial exterior venetian blind were modeled with identical slat geometry (tilt 30° from the vertical window plane) and differ only in slat optical properties: BSDAR and the commercial blind's optical was assigned as same as measurement (Figure 2 and Figure S54). Work‐plane illuminance was computed on a 0.5 m grid of 10 × 8 points at 0.75 m height; uniformity U_0_ is the ratio of minimum to mean over that grid. Daylight Glare Probability (DGP) was evaluated with evalglare (the standard glare‐evaluation tool of the Radiance suite, which identifies glare sources in a fisheye luminance image and returns the DGP) from 180° fisheye renderings taken at seated eye height (1.2 m), facing the window and facing the opposite wall.

The directional infrared transport of the array was quantified separately from the surface‐to‐surface radiation solution of Figure 1, which returns the self‐consistent radiosity balance between surfaces and does not resolve individual ray paths. Emission from the PDMS‐coated glazing was traced in Radiance on the as‐built geometry, with rays launched in cosine‐weighted directions and followed until each terminated by reaching the sky, reaching the ground, returning to the glazing, or being absorbed on a slat. Specular reflection at each slat encounter was applied as d’ = d − 2(d·n)n, with absorption sampled against the measured infrared reflectance, and the terminal fractions were converted to radiative flux per unit glazing area using the measured emissivity. The tracing was applied in two ways. For the array itself, 120,000 rays were traced at the as‐built geometry and the calculation repeated over a range of slat‐to‐glazing gaps and slat pitches. For the comparison with a commercial blind, the same number of rays and the same random seed were used for a bare window, a slat of infrared emissivity 0.90 and BSDAR, so that the three cases differ only in slat optical properties. Directional thermography and slat rear‐surface temperature measurements were carried out to verify the mechanism experimentally. The cost comparison between the BSDAR slats and commercial exterior blinds, together with the resulting payback estimate and its underlying assumptions, is given in Text S5 (Figures S55–S58).

## Author Contributions

Conceptualization: G.H. and X.M.; Methodology: G.H. and X.M.; Investigation: X.M. and G.H.; Visualization: X.M. and G.H.; Funding acquisition: G.H. and X.M.; Project administration: G.H.; Supervision: G.H.; Writing – original draft: X.M. and G.H.; Writing – review & editing: X.M. and G.H.

## Funding

Initiative and Networking Fund of the Helmholtz Association, Helmholtz Investigator Group (VH‐NG‐21‐07, UNICOS) (G.H.), Helmholtz Association Research Field Energy, Topic 1 (ref. 38.01.04) (G.H.), Karlsruhe School of Optics and Photonics (KSOP) (G.H.), Helmholtz–OCPC International Postdoctoral Exchange Fellowship Program (ZD2024010, Karlsruhe Institute of Technology and Southeast University) (X.M.)

## Conflicts of Interest

The authors declare no conflicts of interest.

## Supporting information




**Supporting File**: advs77392‐sup‐0001‐SuppMat.pdf.

## Data Availability

Data is available from corresponding author upon reasonable request.
