# Supplementary material for: Bridging Horizontal to Vertical Radiative Cooling
Source: Adv Sci (Weinh). 2026 Aug 24:e77392. Online ahead of print. doi: 10.1002/advs.77392 (PMC13502264; doi:10.1002/advs.77392)
Supplement: Supplementary file 1 [file ADVS-9999-e77392-s001.pdf]

Supporting Information

Bridging horizontal to vertical radiative cooling

*Xiangyu Meng, and Gan Huang**

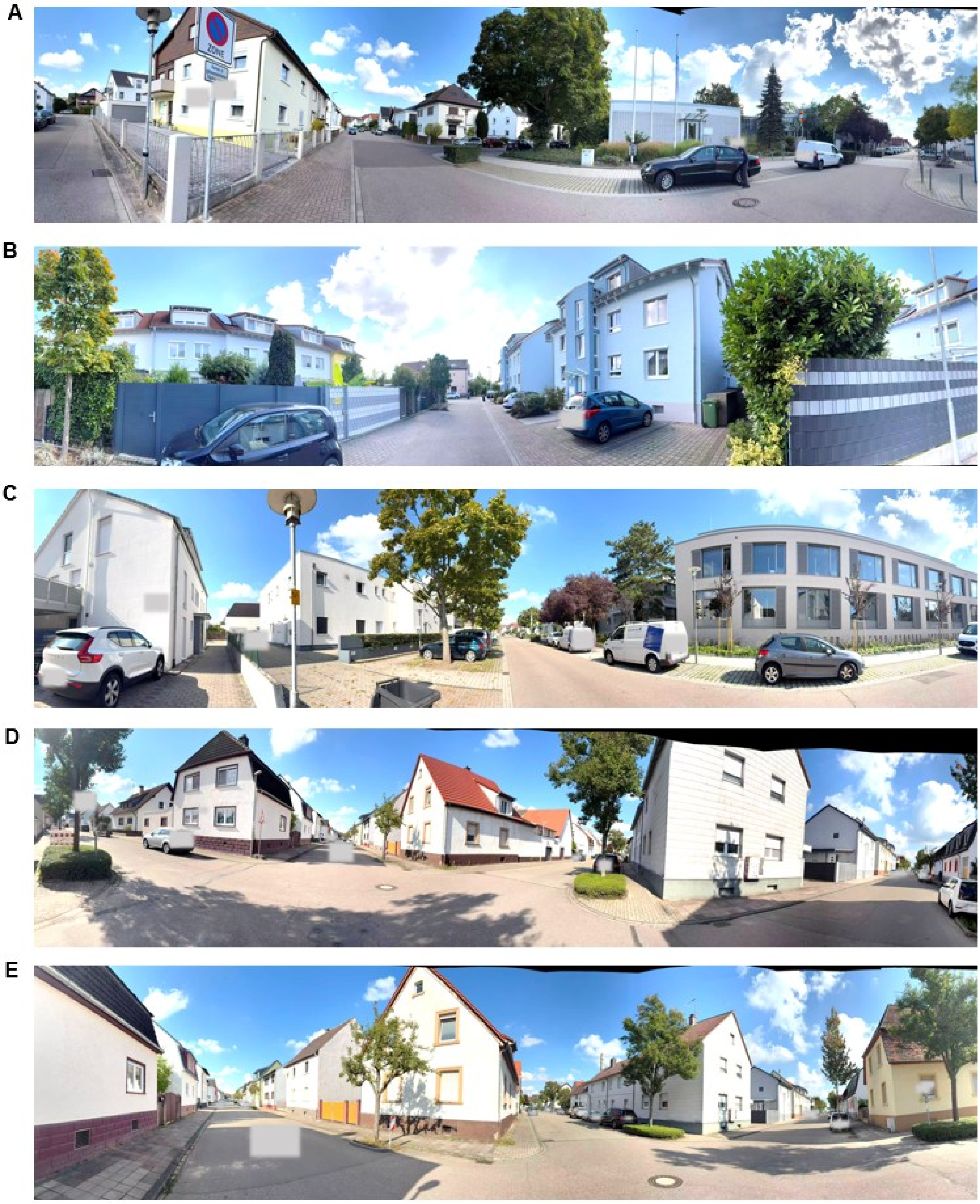

Figure S1. City building views. (**A–E**) Typical residential neighborhood in a German city, illustrating how buildings are commonly arranged along both sides of long, canyon-shaped street. The images above were taken in Eggenstein-Leopoldshafen, Germany, between 10:00 A.M. and 11:00 A.M. on August 29, 2025. The photos were captured using the PANO feature on a mobile phone, with a zoom factor of 1×. To protect privacy, car license plates, human faces, and street information in the images have been blurred.

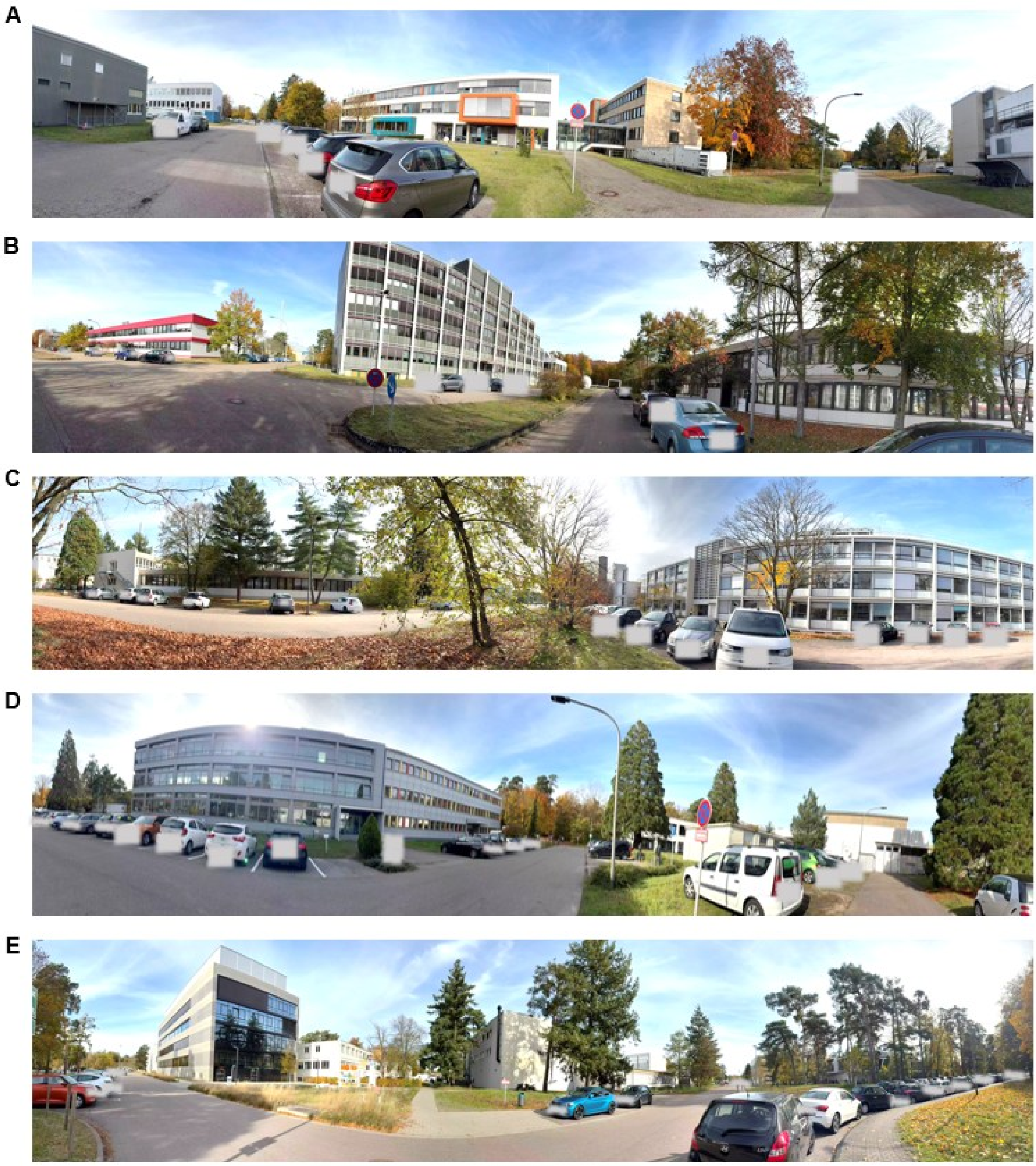

Figure S2. City building views. (**A–E**) Typical office-building in Germany, showing buildings are often arranged along both sides of elongated, canyon-shaped street corridors, with a high proportion of window-covered facades. All the images above were taken in Eggenstein-Leopoldshafen, Germany, between 2:00 P.M. and 3:00 P.M. on September 9, 2025. The photos were captured using the PANO feature on a mobile phone, with a zoom factor of 1×. To protect privacy, car license plates, human faces, and street information in the images have been blurred.

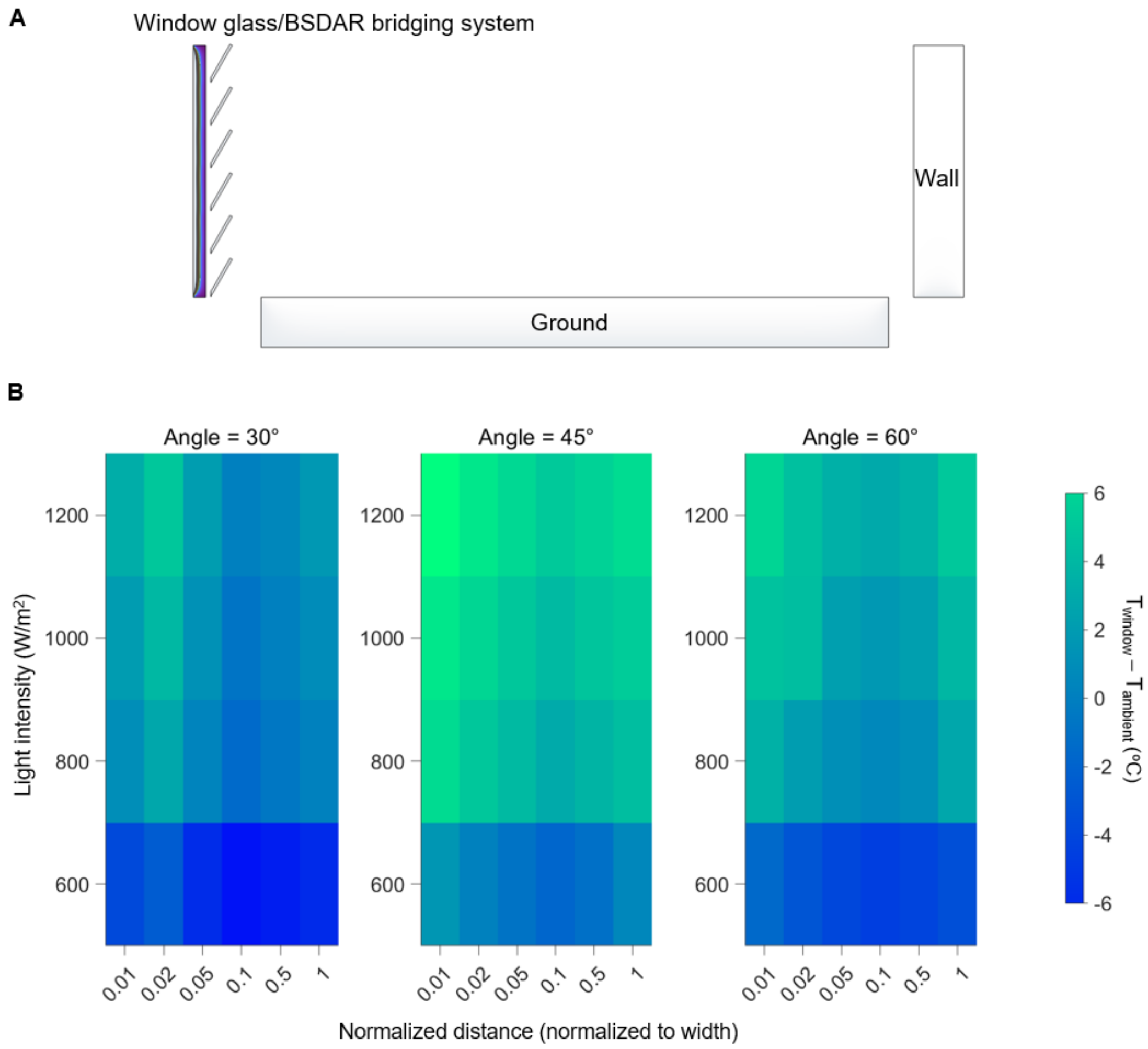

Figure S3. Additional details of the FEA simulation in Figure 1. (**A**) The model used in the COMSOL simulations. See more structure parameter in the following supplementary text S1. Colors within the window show the temperature distribution under surface cooling by PDMS. The window temperature is defined as the average temperature along the central vertical line of the window cross section. (**B**) Heat maps of the window-temperature reduce below ambient, under different solar irradiances for the representative normalized distance (normalized to width) at three typical BSDAR array tilt angles (30º, 45º, 60º). The parameter definitions here are the same as those in Figure 1. By presenting the data in the form of a heat map with limited data points, the reader can more quickly identify the optimized structural parameter ranges, which are still under the conditions of a 30°-angle and a normalized distance close to 0.1.

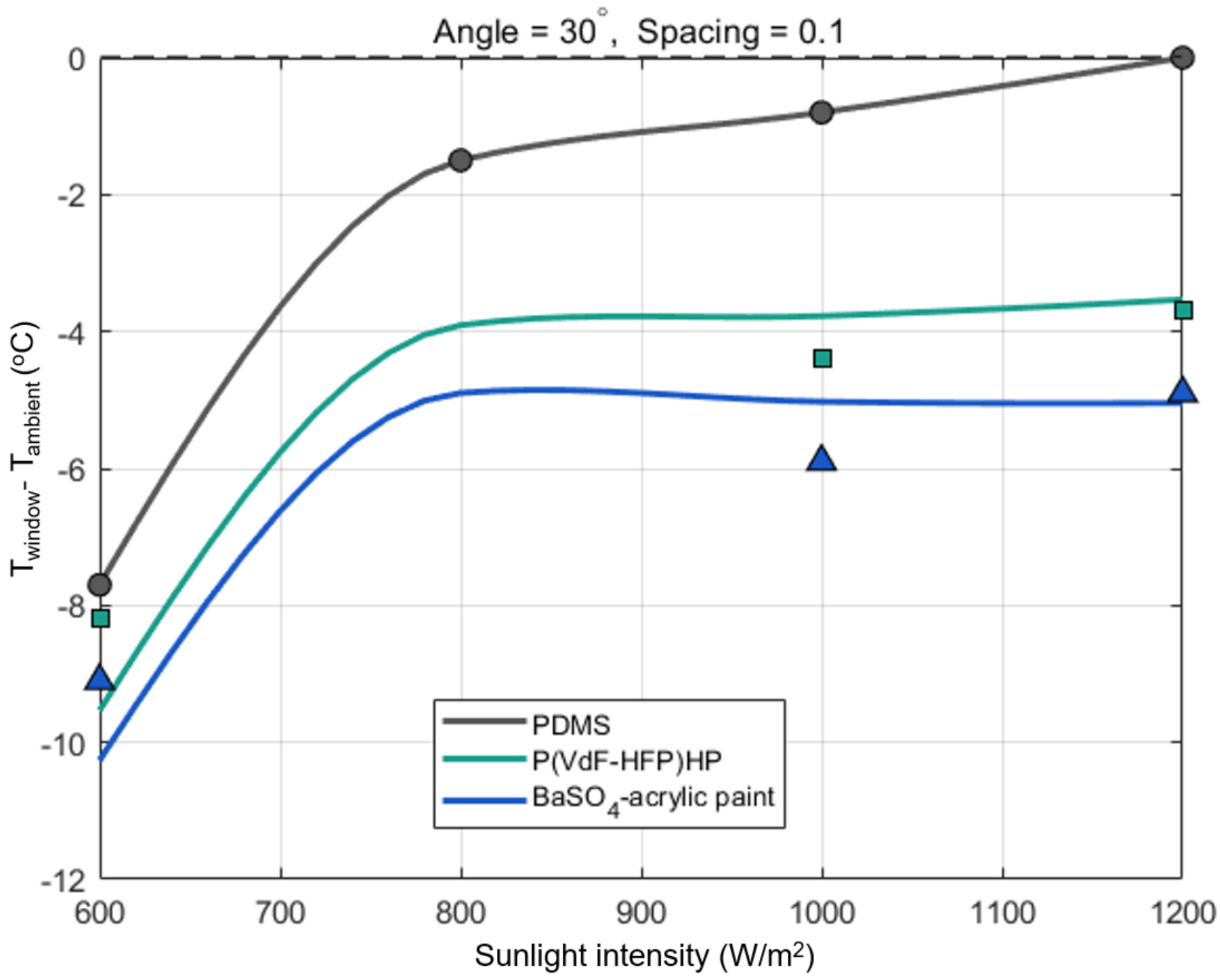

Figure S4. Generality of the BSDAR bridging effect across different radiative-cooling emitters, using the same thermal model as in Figure 1. Window sub-ambient temperature depression, $\Delta T = T_{window} - T_{ambient}$, as a function of incident solar irradiance for three emitters combined with an identical BSDAR array (tilt angle 30°, normalized distance 0.1). The parameter definitions here are the same as those in Figure 1. Solid curves are obtained in MATLAB from a standard radiative-cooling energy balance, in which the spectral emissive power is integrated band-by-band over the Planck distribution; symbols are the corresponding results of the COMSOL model used throughout the manuscript, computed at three representative irradiances. The two routes are fully independent; black, PDMS as used throughout this work; green, hierarchically porous P(VdF-HFP)HP; blue, ultra-white $BaSO_4$-acrylic paint. All three emitters remain sub-ambient over the entire irradiance range examined, indicating that the bridging function is set by the array geometry rather than by the choice of emitter. Values are computed under the idealized boundary conditions of Figure 1 and represent the intrinsic cooling potential of each emitter under identical assumptions, which isolates the effect of the emitter itself.

Supplementary Text

S1. Methods in COMSOL simulations

A steady-state two-dimensional COMSOL model was constructed to evaluate the cooling behavior of the BSDAR/window. The geometric setup is illustrated in Figure S3. To enable application in real building environments, all dimensions used here are macroscopic and expressed in meters, and the actual size of standard windows is taken into consideration. The window is modeled as a 1 m–tall vertical segment with a thickness of 0.05 m, while the opposite wall is also 1 m tall with a thickness of 0.2 m. The ground between the two buildings spans 2.5 m in width and is modeled with a thickness of 0.2 m. These dimensions represent a representative facade-scale window segment, allowing the radiative and thermal interactions between the window, the BSDAR structure, the ground, and surrounding buildings to be captured while avoiding scale-specific assumptions. The 1 m window height represents the combined vertical span of multiple floors in a typical mid-rise facade of city building, enabling the model to capture the aggregated thermal behavior of a continuous window band rather than a lab-scale single-window unit. The 0.05 m window thickness serves as an effective value accounting for glazing layers, frames, and air gaps, allowing the facade's thermal behavior to be accurately captured in the model. The ambient temperature was fixed at 22 °C. The opposite building's wall has a thermal conductivity of 2 W/(m·K), and the ground has a thermal conductivity of 1.1 W/(m·K). The window is made of Corning 7940 quartz glass from the material library, while the reflector is modeled using aluminum from the material library, supplemented with experimentally measured properties to refine its optical and thermal parameters.

The BSDAR bridging structure was discretized into six modular reflector units along the 1 m window height, ensuring full coverage in the closed configuration. The units were arranged with a uniform center-to-center vertical pitch equal to the unit width, which remains fixed for all rotation angles. To reduce geometric complexity, Width was used as the characteristic length scale, and the minimum horizontal separation between the BSDAR array and the window was expressed as a normalized distance (distance normalized to width), as shown in Figure 1C and 1D. This dimensionless formulation allows the optimized configuration to be linearly scaled to windows of arbitrary height in practical building applications.

We employed the COMSOL Surface-to-Surface Radiation module to conduct radiative heat transfer. The ray-shooting approach was chosen to account for specular reflections. To handle multiple reflections and absorptions within the structures, the maximum number of reflections

in the ray-shooting algorithm was set to 1,000, which ensures convergence. The view factors for the wall–sky and wall–ground interactions are automatically evaluated by the COMSOL Surface-to-Surface Radiation module. Four wavelength bands with piecewise constant optical properties were considered: 0–2.5 μm (solar), 2.5–8 μm, 8–13 μm (ATW), and >13 μm. The right surface of window was set as the emission layer with the optical properties same as the tested parameter of PDMS (75 μm) using the piecewise function over different wavelength ranges. The BSDAR structures were set as broadband specular reflections. The wall of the opposite building was treated as ideal broadband emitters ($\varepsilon = 1$ for $\lambda \geq 2.5$ μm). α_solar was set as 0.05. The ground with a constant emissivity of $\varepsilon = 0.95$ for all wavelengths, was maintained at the fixed temperature. The sun illuminates the system at a zenith angle φsun, here focus on φsun = 30° as a representative afternoon condition for the southern U.S. The sky emittance is angle-dependent, given by:

$\varepsilon_{sky}(\lambda,\theta)=(1/\cos\theta)\times[1-(1-\varepsilon_{sky,\ zenith}(\lambda))]$

where $\varepsilon_{sky,\ zenith}$ is the sky emittance at the zenith according to the US Standard 1976 Atmosphere Model. The piecewise sky emittance used in the simulations is a simplified average, weighted by the blackbody spectrum at T_{amb}. In the Surface-to-Surface Radiation module, external radiation sources such as the sky and the sun are included. The solar heat flux is used to set different irradiance intensities as input.

The Solid Heat Transfer module is used to model thermal conduction within the structure. The initial temperature of all structural surfaces is set to ambient temperature. Air domains are defined outside the window and reflector surfaces, while the actual exposed outer surfaces of the ground and the opposite building wall are assigned incident beam power consistent with the irradiance of the external radiation sources defined in the Surface-to-Surface Radiation module. Here, we account for heat conduction through the window thickness as well as convective heat transfer with the surrounding air. The window temperatures shown in Figure 1 were obtained by post-processing the COMSOL results: a vertical line was drawn through the center of the window to extract the temperature profile, and the average temperature along this line was taken as the representative window temperature. The window temperature data were then subtracted from the initial ambient temperature to obtain the temperature difference between the window and the ambient, which is presented in Figure 1. Figure S3B shows window-temperature heat maps for several representative array tilt angles and normalized distance, allowing readers to examine the detailed temperature trends.

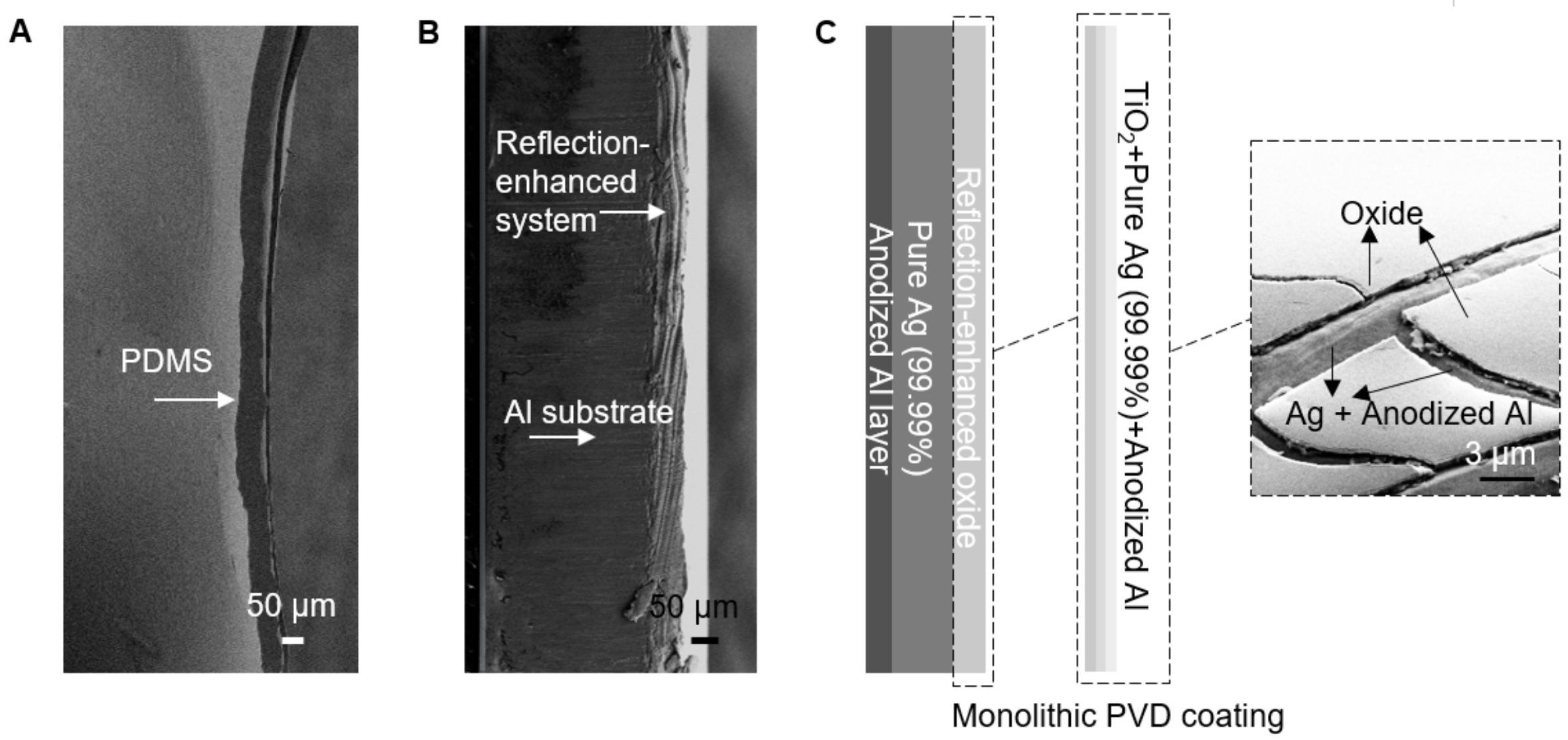

Figure S5. Detailed structures of the PDMS film and the multilayer reflection-enhanced (MLRE) reflector. (**A**) SEM image of PDMS film. (**B**) SEM image of MLRE. The MLRE reflector comprises a pure-aluminum (Al) substrate and a reflection-enhanced coating system (RECS) on the front (light-incident) side. The nominal layer thicknesses are summarized in the text associated with Figure 2A. (**C**) Schemes illustrates the RECS stack, with an inset SEM image (right) showing the PVD-coated RECS surface. The high-purity Ag layer provides excellent reflectivity, particularly in the visible and near-infrared spectra, due to its intrinsic high reflectance properties. When a TiO_2 layer is deposited on the Ag surface, it enhances this reflectivity through several key mechanisms. Firstly, TiO_2's high refractive index enables constructive interference effects, especially in the UV and visible ranges, by controlling its thickness to optimize light reflection. This creates multiple reflections and interference between the TiO_2 and Ag layers, leading to a significant increase in reflectivity. Additionally, the TiO_2 layer serves as a protective barrier, preventing the oxidation of the Ag surface, which helps maintain the Ag's high reflectivity over time. Together, these effects work to substantially boost the system's overall reflectivity. The anodized Al layer offers protection and contributes to the overall stability of the structure. This high-density, continuous nature of the PVD coating ensures uniform optical performance, providing exceptionally high reflection across a broad spectral range. Because the PVD-deposited layers exhibit dense microstructures and indistinguishable interfaces under SEM, individual layers cannot be directly resolved. To estimate the TiO_2 top-layer thickness, we introduced controlled surface scribing using a hand-held diamond knife and applied slight mechanical stress to generate microcracks. Under SEM, partial local delamination of the TiO_2 layer from the underlying Ag layer was observed at the

crack sites, enabling thickness estimation from multiple cracks. As illustrated in the RECS schematic, the RECS stack consists of a TiO_2 top layer, a high-purity Ag reflective layer, and an anodized Al interlayer adjacent to the Al substrate, which provides mechanical support. Thickness values provided by the manufacturer are listed in the text associated with Figure 2A.

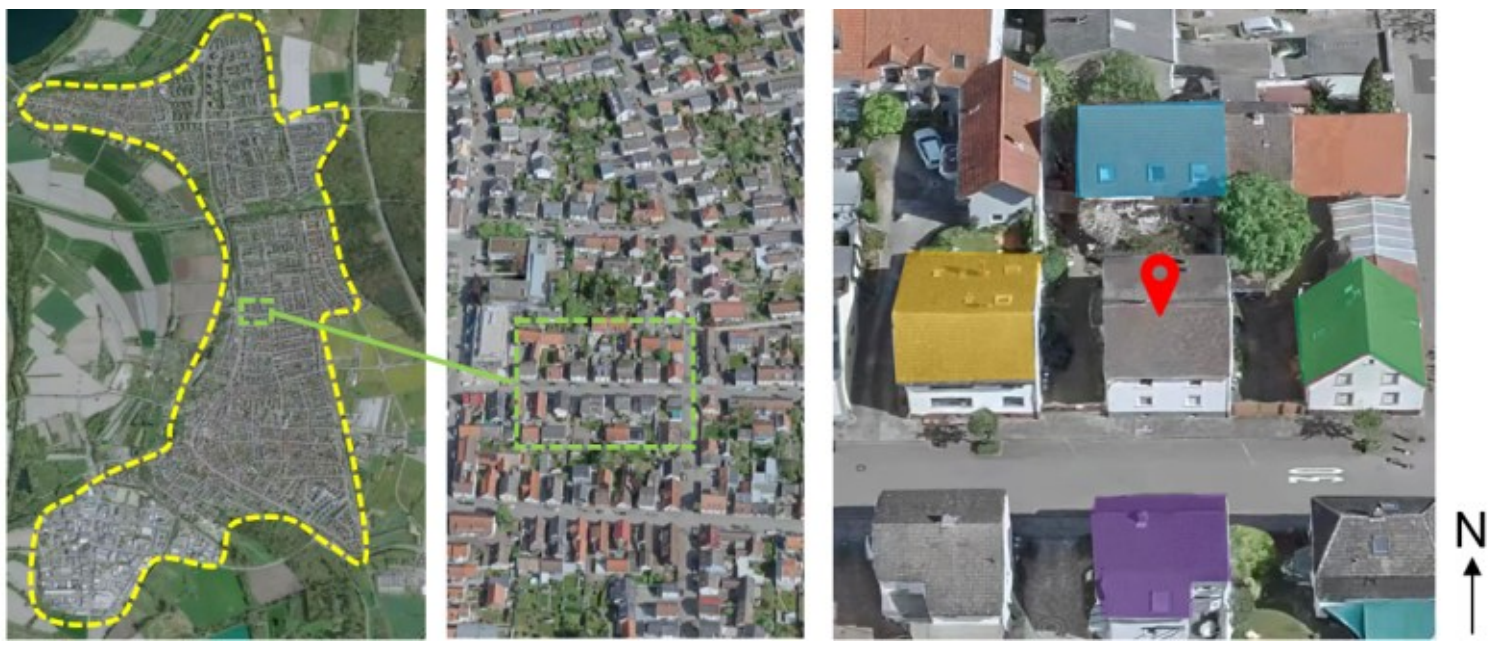

Figure S6. Detailed city building environment in the cooling performance tests in Figure 2. The map and optical images for showing the testing locations and environment in Figure 2. The dashed circle in left highlights the city shape, the dashed boxes in left and in middle highlight the test location and surrounding, the location sign in right highlights the test building. The surrounding buildings are colored for better showing the directions, and the colors are same as the ones in the Figure 2 and same as the frame's color of infrared images in following Figure S9–S13. Three images use a same direction axis. The detailed test location in Figure 2 is in Eggenstein-Leopoldshafen, Germany. The testing was permitted by the house owner.

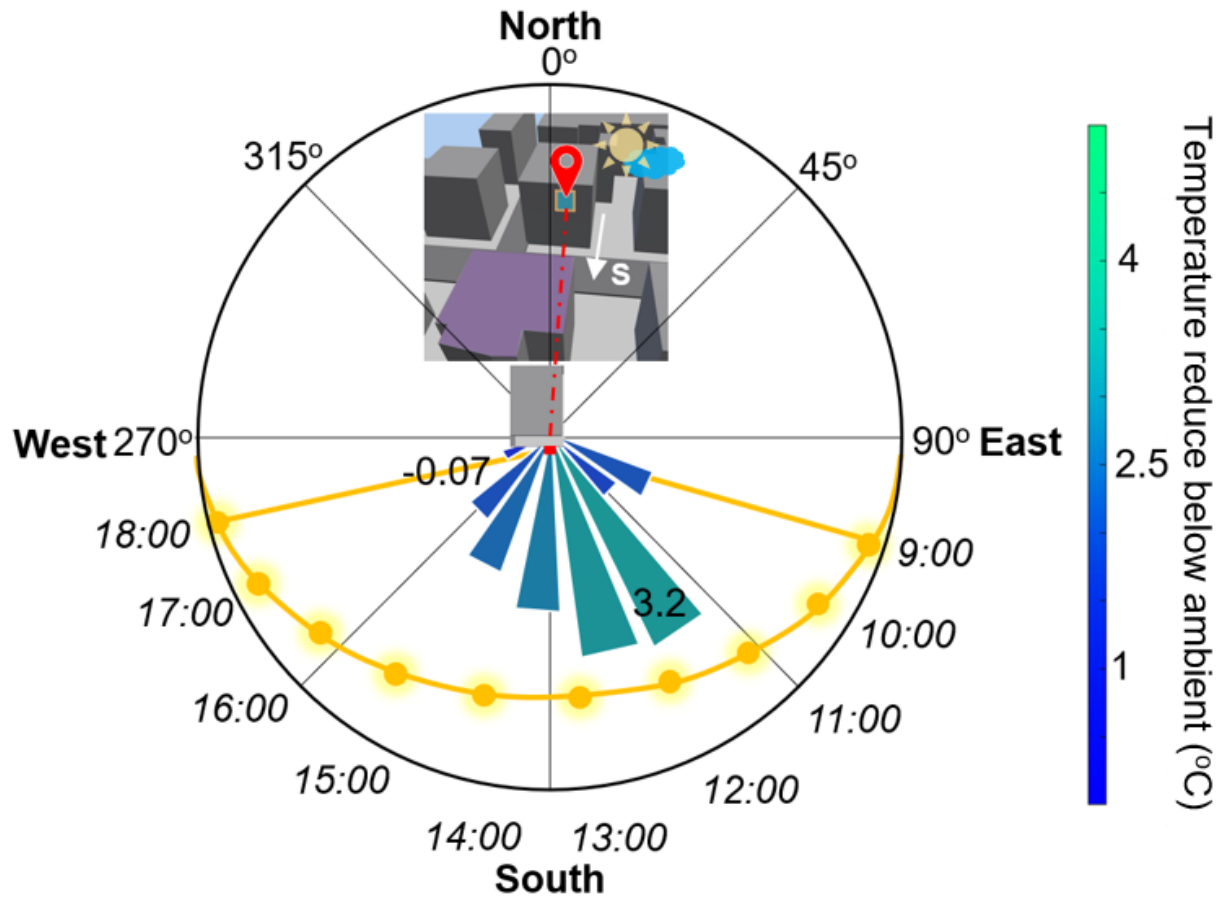

Figure S7. Daytime sub-ambient cooling of a south-facing window under partly cloudy condition. Yellow markers indicate the solar trajectory during testing. The centered top-view schematic shows the instantaneous angle between the window normal and the solar direction. The upper inset depicts the surrounding built environment, and the lower fan-shaped bar reports the hourly averaged below-ambient temperature reduction (color scale at right; maximum and minimum values indicated), where positive values denote temperatures below ambient. Supporting data under the same weather are provided in Figure S10 (temperature vs. time curves and an infrared image of the opposite building), Figure S14 (ambient light intensity, relative humidity, wind speed). Even under partly cloudy conditions, the PDMS/glass window equipped with the BSDAR structure achieves an average of 1.5 °C below-ambient cooling over the full daytime period (9:00 A.M.–6:00 P.M.). As cloud cover decreased, the maximum hourly-averaged below-ambient reduction reached 3.2 °C. Because high-altitude clouds are typically colder than near-ground ambient air, a partially clouded sky can still serve as an effective radiative heat sink for the building structure. These results demonstrate robust performance across variable weather and support broad applicability.

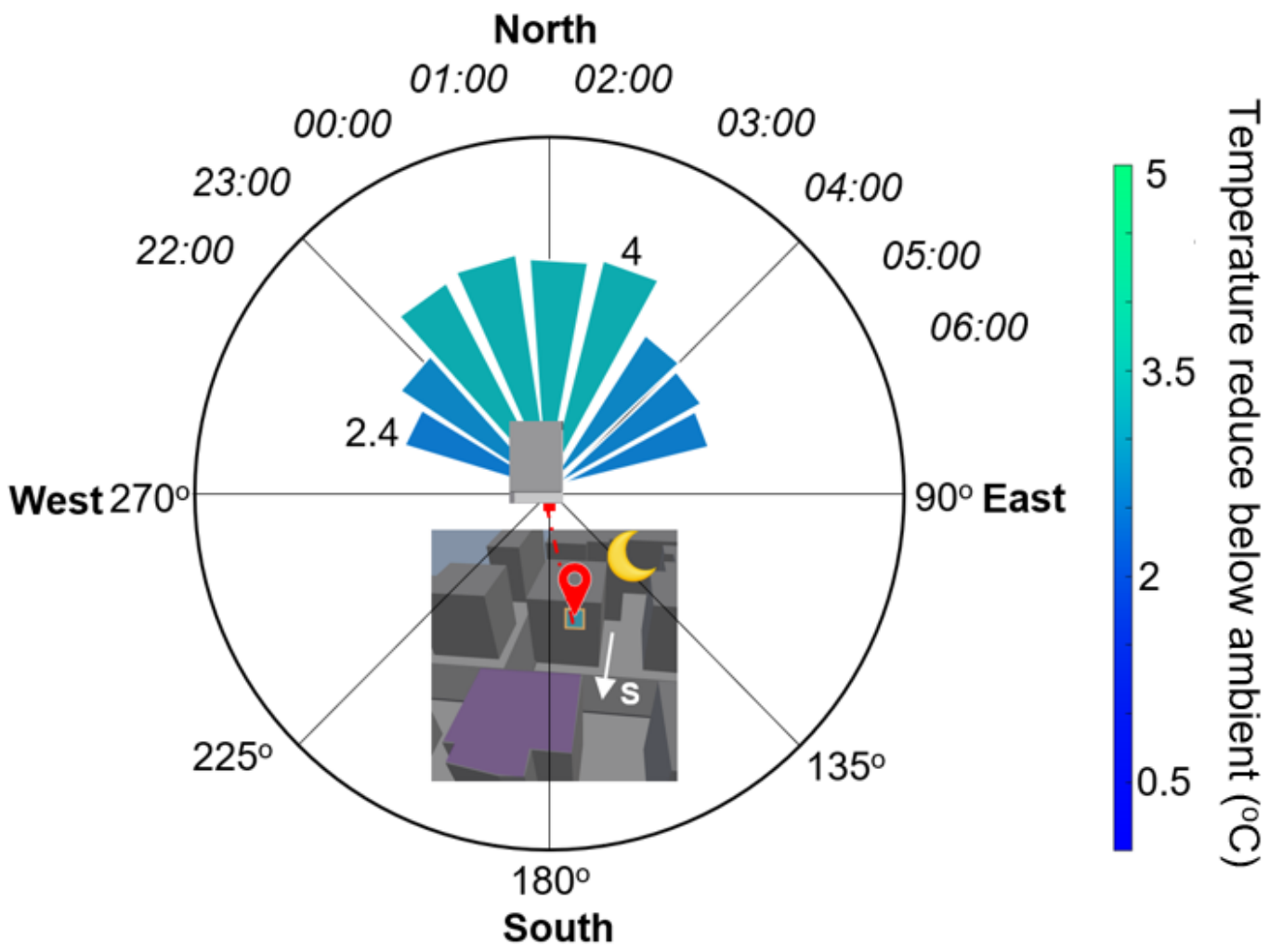

Figure S8. Nighttime sub-ambient cooling for the south-facing window. The top-view schematic (center) marks the tested window on the building. The lower inset shows the surrounding built environment. The fan-shaped bars at the top report the hourly averaged sub-ambient temperature reduction (color scale at right; maximum and minimum values indicated), where the reduction is defined such that positive values correspond to temperatures below ambient. Experimental procedures are described in the Materials and Methods. Nighttime measurements (temperature vs. time traces in Figure S9) show that the PDMS/glass window mounted with the BSDAR structure achieves an average 3.3 °C reduction below the nighttime ambient temperature over the full night (10:00 P.M.–6:00 A.M.). The maximum hourly-averaged reduction reaches 4.0 °C below ambient. This nighttime cooling is slightly stronger than the daytime performance (Figure 2F) of the same sample at the same south-facing location, consistent with clearer sky conditions and reduced solar-driven environmental heating at night (*e.g.*, lower temperatures of the opposite buildings and the ground; infrared image in Figure S9).

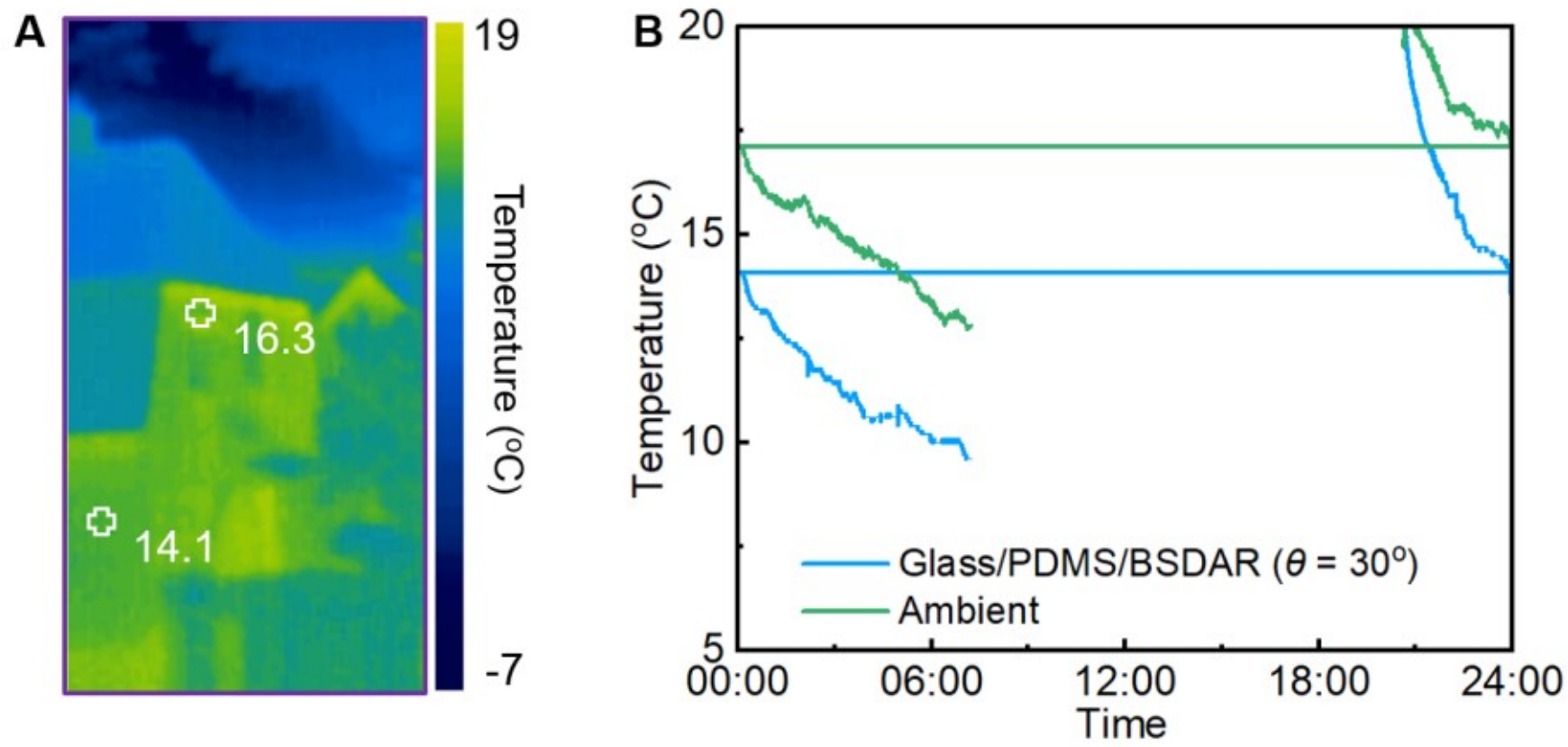

Figure S9. Detailed test environment and temperature vs. time curve for nighttime cooling on the south-facing window. (**A**) Infrared image of the opposing building facade during nighttime, this building is opposite to the south-facing test window. (**B**) Temperature vs. time curve for the south-facing window during nighttime.

The infrared image was captured at 1:00 A.M. Owing to the low radiative contrast under nighttime conditions, the infrared image appears slightly blurred compared with other infrared images in this work. Nevertheless, surface temperatures of the opposing building can be extracted as references using the color legend and temperature readout provided by the imaging software. The temperature-time trace spans from 10:00 P.M. to 6:00 A.M., with the time axis running from right to left.

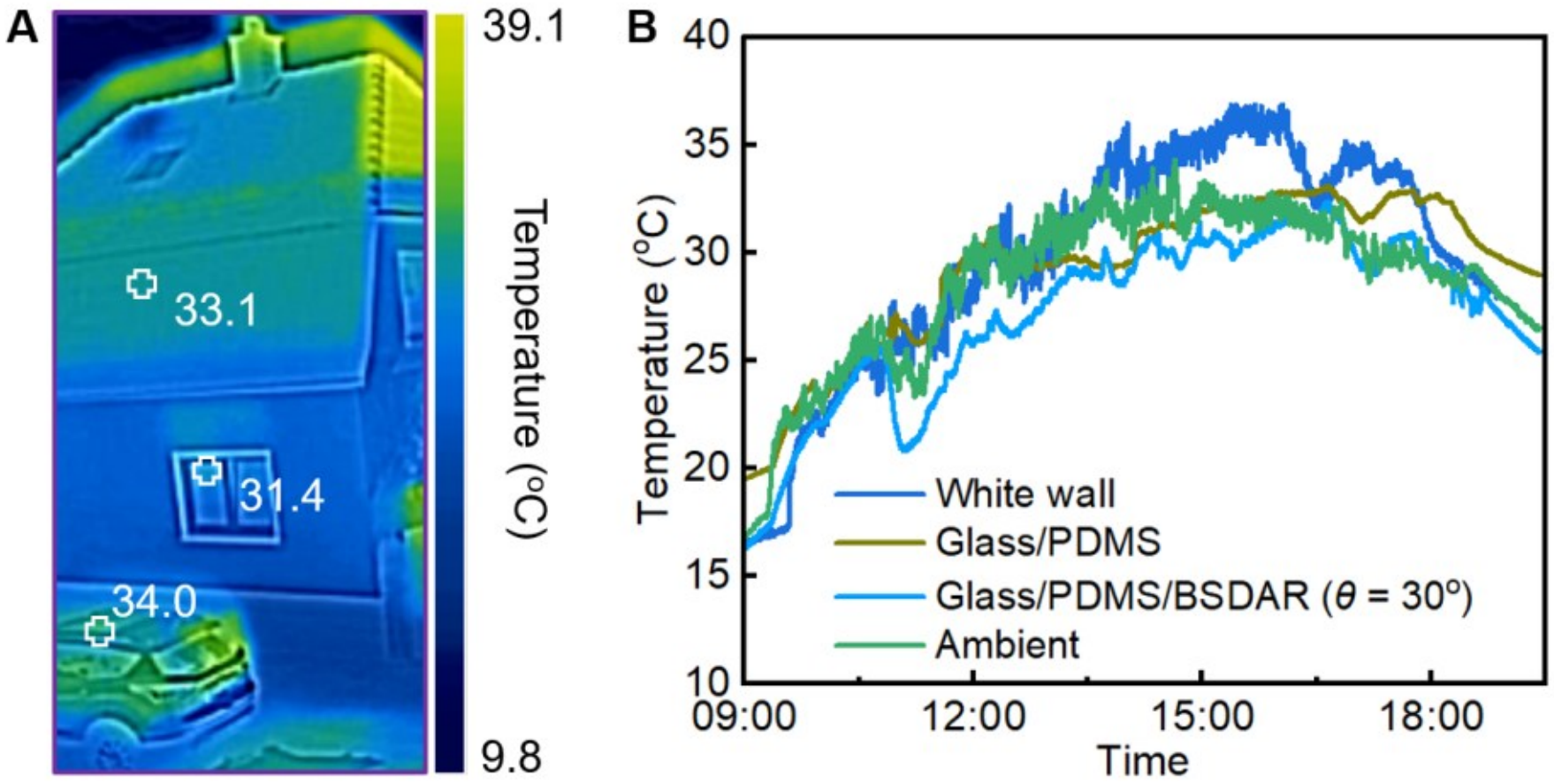

Figure S10. Detailed test environment and temperature vs. time curve for daytime cooling of the south-facing window under partly cloudy condition. (**A**) Infrared image of the opposing building facade acquired during daytime under partially cloudy weather; the opposing facade is directly opposite the south-facing test window. (**B**) Temperature vs. time curves measured for the south-facing window and compared samples under partially cloudy weather. The infrared image was acquired at 12:30 P.M. Environmental light intensity, relative humidity, and wind speed are provided in Figure S14. During this test, temperatures were recorded for a PDMS/glass window mounted with the BSDAR structure, a PDMS/glass reference window without BSDAR, the building's white wall at the same height (with the sensor fixed using a thermally conductive but opaque copper tape), and the ambient temperature. Consistent with the environmental records (Figure S14), after 3:20 P.M. the BSDAR-mounted sample gradually shifted from below-ambient cooling to values near ambient (or intermittently above ambient), coincident with a rapid increase in cloud cover between 3:00 P.M. and 5:00 P.M., which weakened radiative heat exchange with the sky. The within-day contrast between the lower-cloud period (10:00 A.M.–3:00 P.M.) and the higher-cloud period (3:00 P.M.–6:00 P.M.) highlights the stronger benefit of the BSDAR structure under clearer-sky conditions.

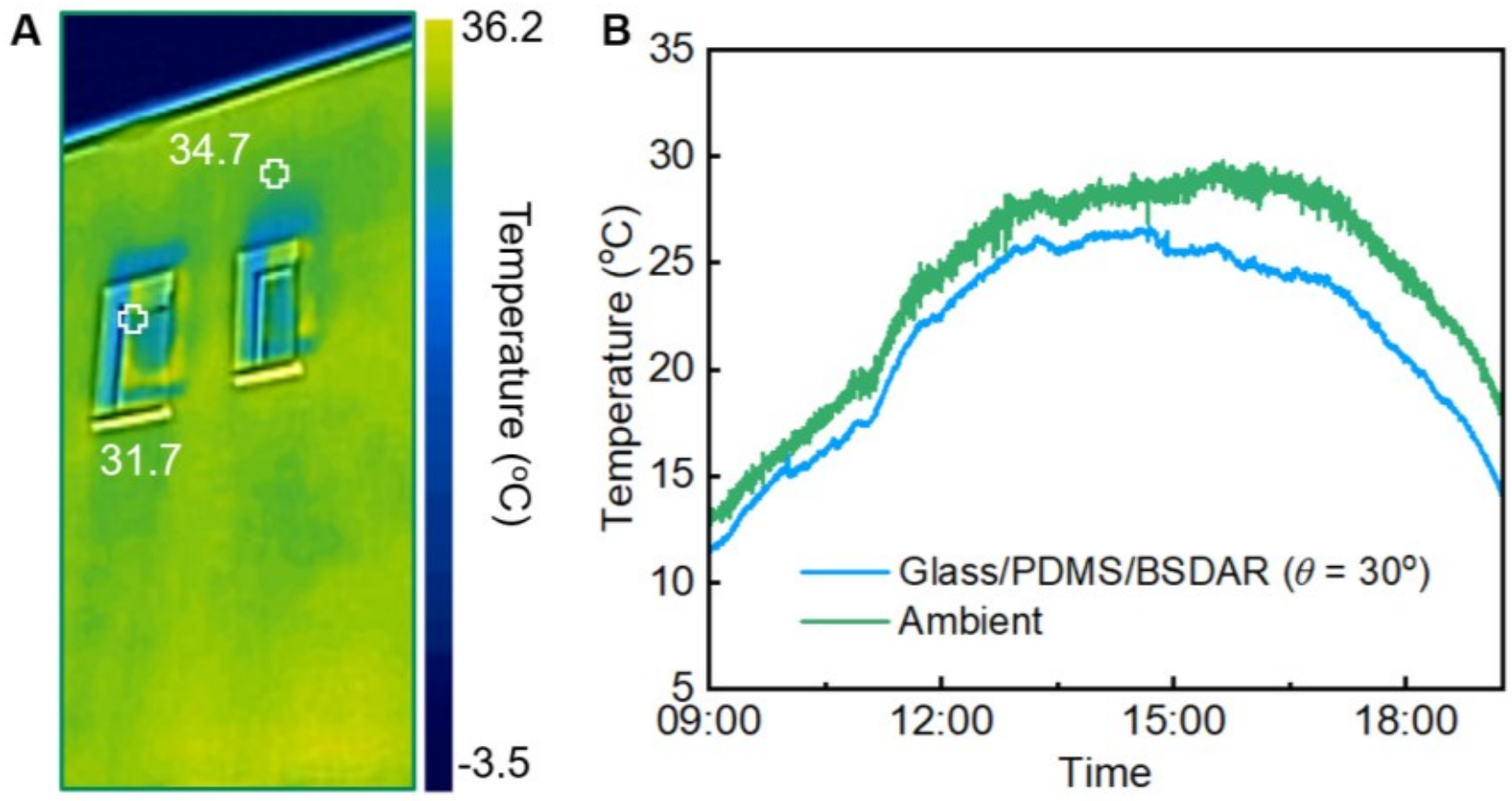

Figure S11. Detailed test environment and temperature vs. time curve for daytime cooling of the east-facing window. (**A**) Infrared image of the opposing building facade acquired during daytime; the opposing facade is directly opposite the east-facing test window. (**B**) Temperature vs. time curves of the east-facing window measured during daytime.

The infrared image was acquired at 1:45 P.M., environmental light intensity, relative humidity, and wind speed are provided in Figure S14. Combined with the solar trajectory and window position in Figure 2G, the below-ambient cooling of the BSDAR-mounted sample varies over the day: cooling is weaker during periods of more direct solar incidence in the morning and stronger when the window becomes oriented away from the solar direction in the afternoon. Nevertheless, the system achieves an average reduction of 2.6 °C below ambient over 9:00 A.M.–6:00 P.M., with a maximum hourly-averaged reduction of up to 4.0 °C below ambient, demonstrating effective performance for the east-facing orientation.

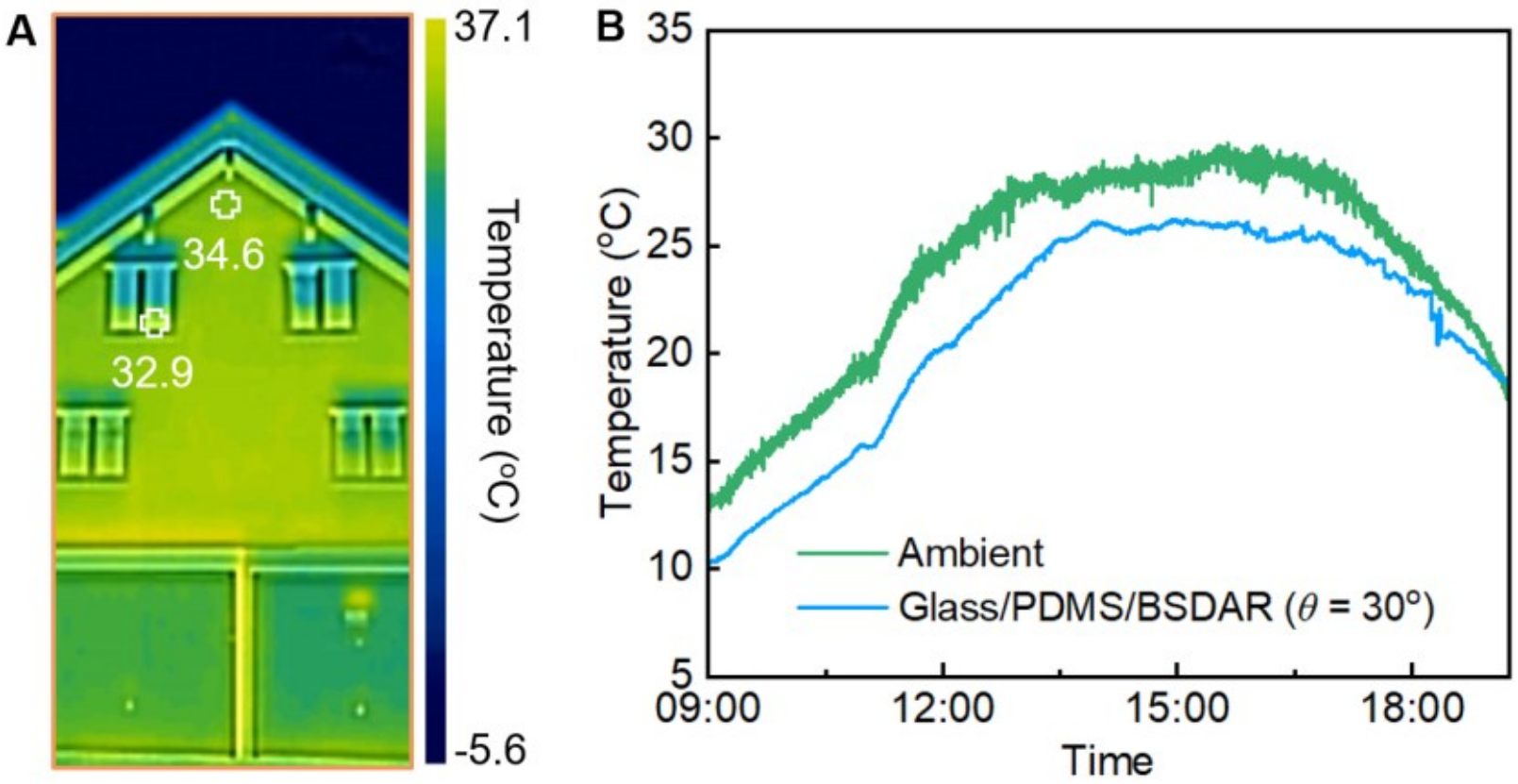

Figure S12. Detailed test environment and temperature vs. time curve for daytime cooling of the west-facing window. (**A**) Infrared image of the opposing building facade acquired during daytime; the opposing facade is directly opposite the west-facing test window. (**B**) Temperature vs. time curves of the west-facing window measured during daytime.

The infrared image was acquired at 3:20 P.M. Environmental light intensity, relative humidity, and wind speed are provided in Figure S14. Combined with the solar trajectory and window position in Figure 2H, the below-ambient cooling of the BSDAR-equipped sample varies over the day: cooling is weaker in the afternoon when the west-facing window experiences more direct solar exposure and stronger in the morning when the window is oriented away from the solar direction. Consistent with the local solar trajectory during the test day, the afternoon west-facing period corresponds to a smaller incidence angle (more direct solar incidence), resulting in higher solar loading and thus slightly smaller cooling than in the east-facing case. Nevertheless, over 9:00 A.M.–6:00 P.M., the system achieves an average below-ambient reduction of 2.9 °C, with a maximum hourly-averaged reduction reaching 4.3 °C below ambient, demonstrating robust performance for the west-facing orientation.

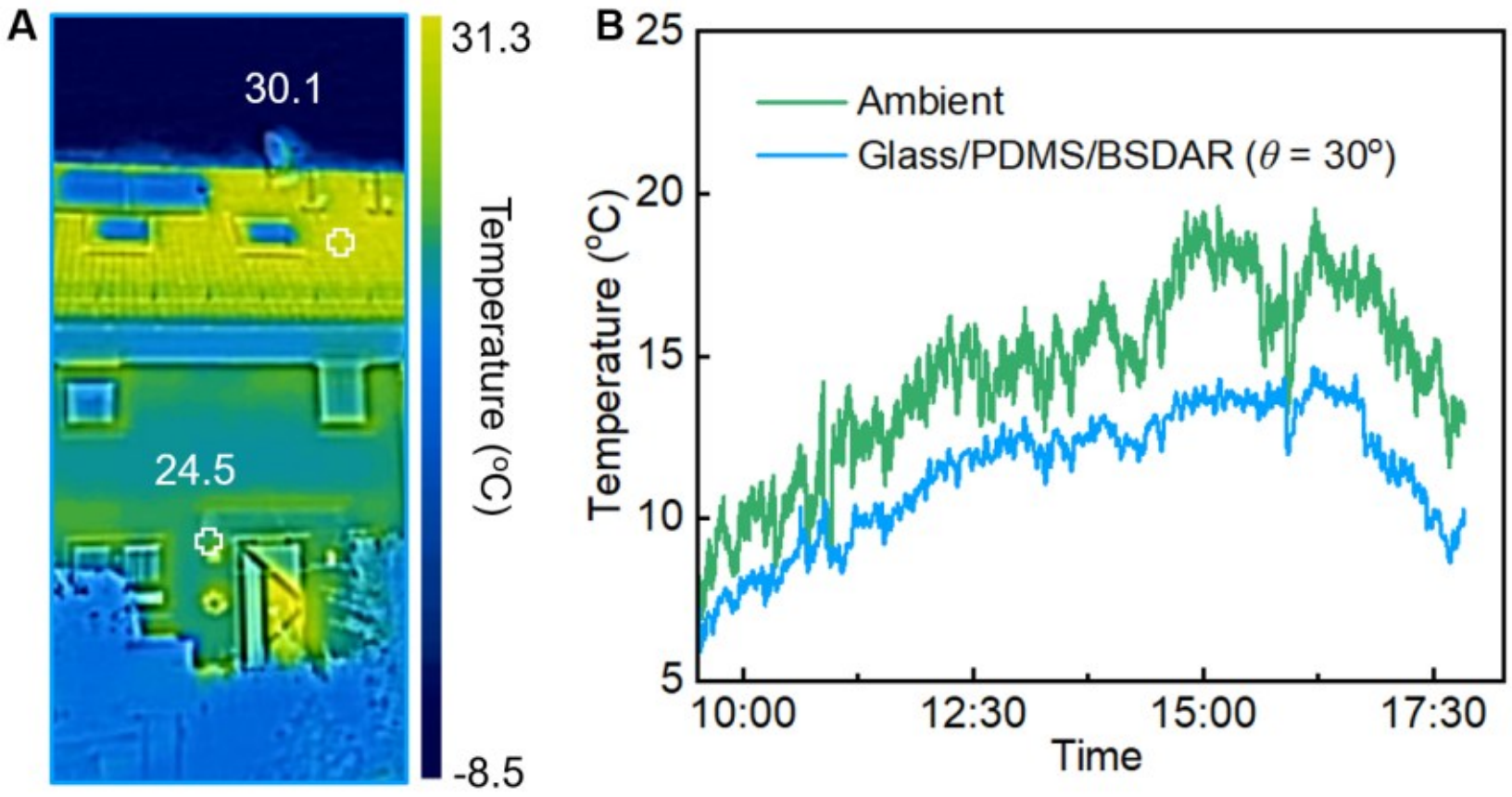

Figure S13. Detailed test environment and temperature vs. time curve for daytime cooling of the north-facing window. (**A**) Infrared image of the opposing building facade acquired during daytime; the opposing facade is directly opposite the north-facing test window. (**B**) Temperature vs. time curves of the north-facing window measured during daytime.

The infrared image was acquired at 3:00 P.M. Environmental light intensity, relative humidity, and wind speed are provided in Figure S14. Although the north-facing facade receives less direct solar incidence around noon, in the real street-canyon environment of the test site it can still experience substantial radiative heat loading from the sunlit opposing facades and the ground. Nevertheless, over the full daytime period, the BSDAR-mounted sample achieves an average reduction of 3.0 °C below ambient, with a maximum hourly-averaged reduction of up to 3.8 °C below ambient, demonstrating robust performance for the north-facing orientation.

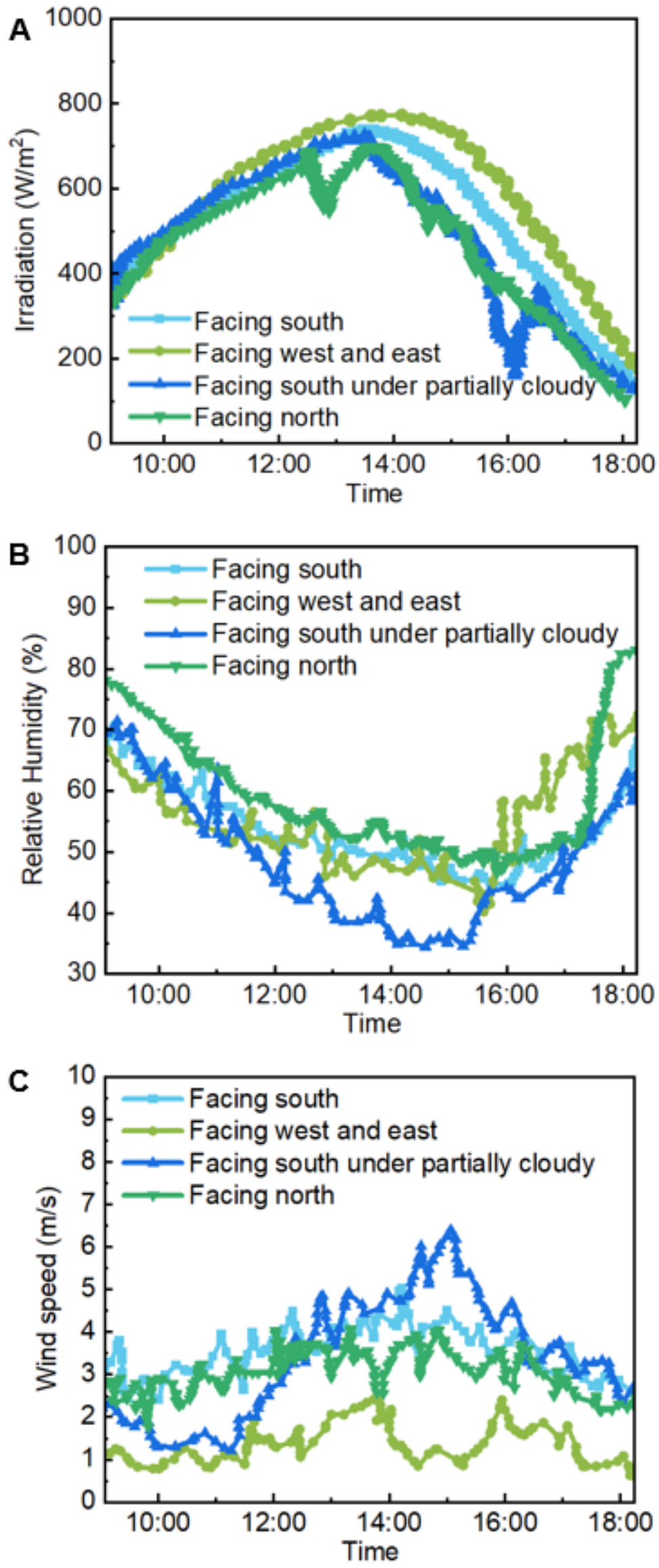

Figure S14. Detailed environmental light irradiation (A), relative humidity (B) and wind speed (C) during tests in Figure 2.

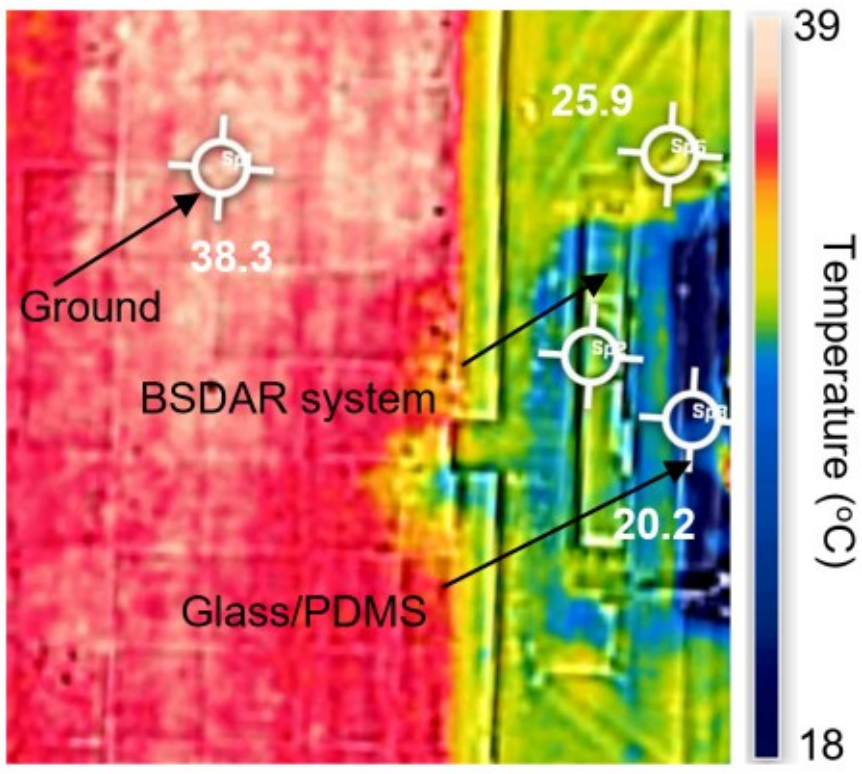

Figure S15. Infrared image of the south-facing BSDAR-mounted window, showing the temperature distribution across the device structures. This infrared image was taken on the south-facing window tested in Figure 2, with the BSDAR device extending 40 cm outward from the window edge and viewed from a top-down oblique angle. Infrared-camera settings used an emissivity at 0.95 and the relative humidity at 50%, consistent with the average humidity during the test and applied for consistent temperature readout. From the temperature distribution it can be seen that the PDMS/glass region behind the BSDAR has the lowest apparent temperature, remaining below the ambient air temperature and below the exterior white wall and the ground, demonstrating that BSDAR can effectively reduce the temperature of a window facade coated with a horizontal radiative-cooling material. The glass region is a high-emissivity surface, so its reading is its own temperature. The slats are specular in the infrared and their reading is instead that of whatever they reflect along the line of sight; at the oblique angle used here they reflect part of the sky, and their apparent temperature is correspondingly low. This directional behavior is examined explicitly in Figure S50, where the same array is imaged simultaneously from the front and from directly above.

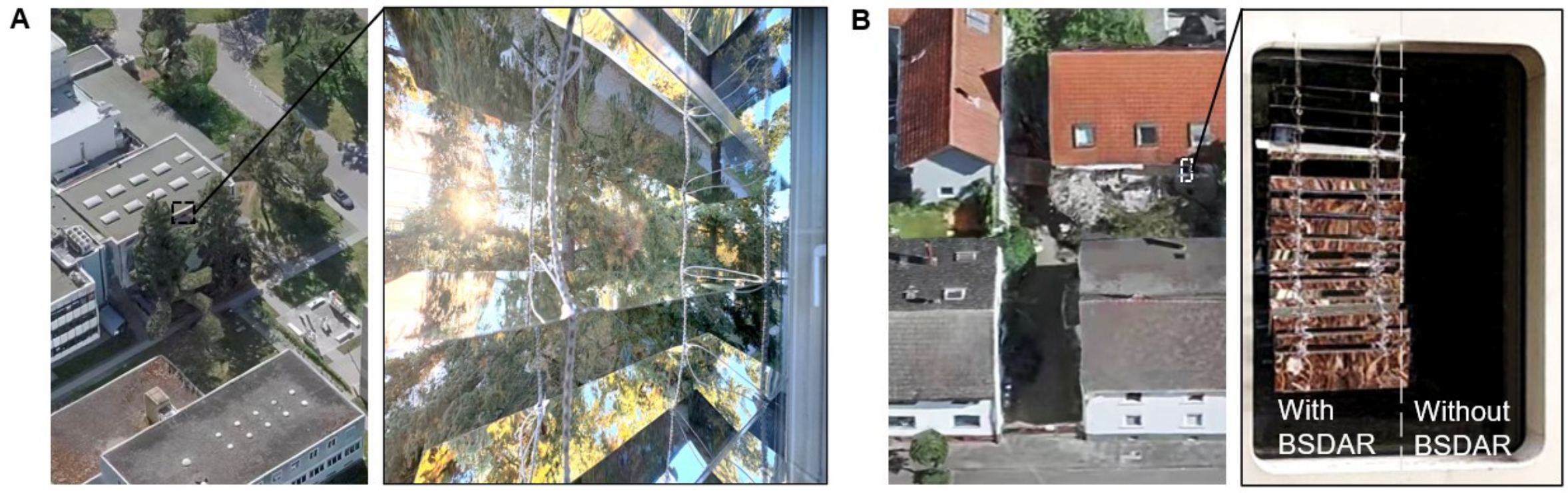

Figure S16. Detailed test building environment and optical images of BSDAR after scaled engineering. (**A**) Application of the enlarged BSDAR on office building windows, demonstrating its adjustable angle for controlling light transmission while achieving full transparency. The test location is at Eggenstein-Leopoldshafen, Germany. (**B**) Application of the enlarged BSDAR on a residential building window, with window sections with and without the external BSDAR for temperature comparison testing. The location is at Eggenstein-Leopoldshafen, Germany.

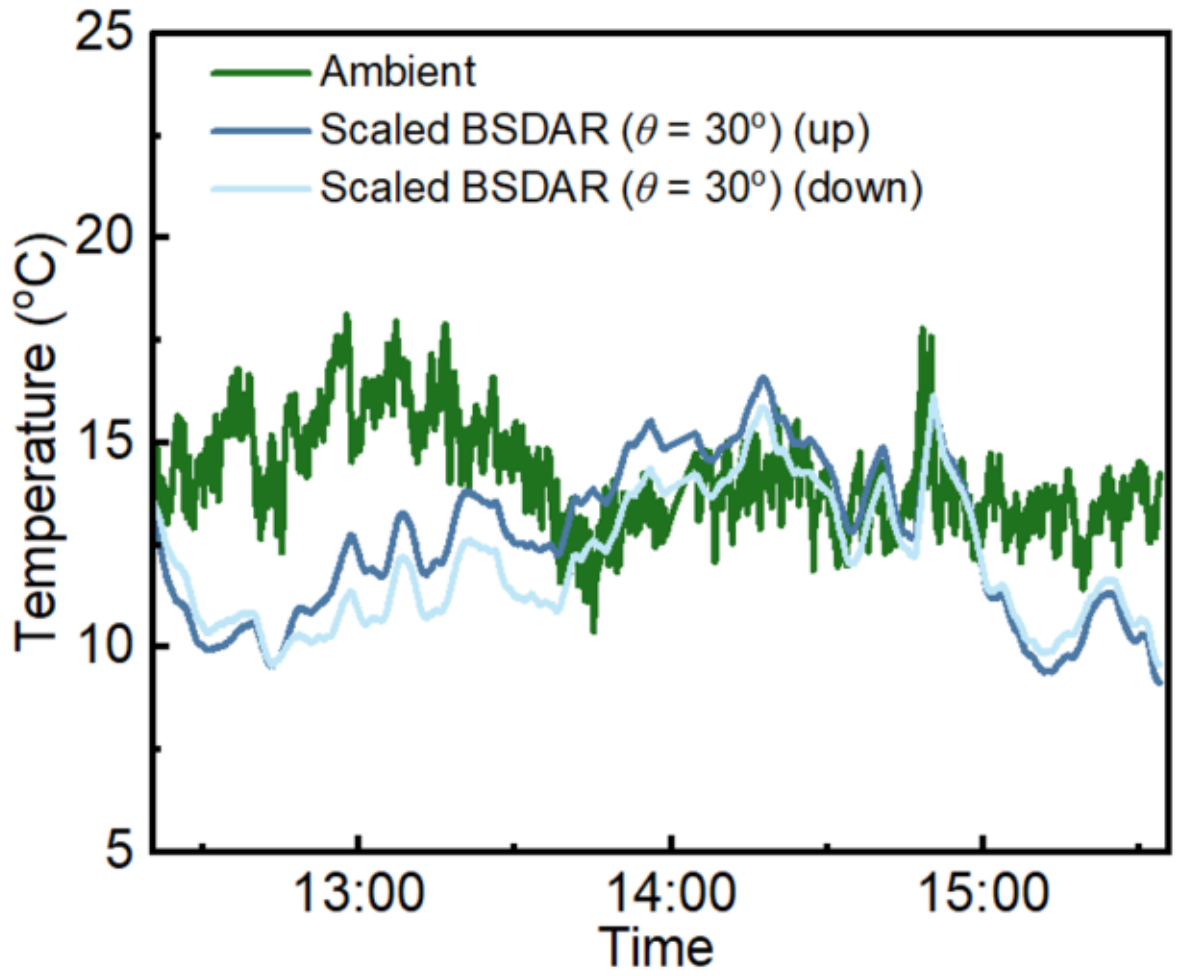

Figure S17. Temperature vs. time curves for the south-facing, engineered BSDAR under variable cloud cover weather. The tested window location, highlighted in Figure S16, is in Eggenstein-Leopoldshafen, Germany. “Upper” and “lower” denote two measurement locations along the vertical direction of the scaled BSDAR; the sensor frame was attached to the second BSDAR unit from the top and the second BSDAR unit from the bottom, respectively. Environmental light intensity, relative humidity, and wind speed during the test are provided in Figure S18. The results show that, under passing-cloud conditions, the engineered BSDAR system enables the PDMS/glass window to reach temperatures below the ambient temperature during cloud gaps. Although the below-ambient cooling is temporarily weakened during periods of increased cloud cover and humidity, the measurements demonstrate feasible cooling under variable weather. The temperature difference between the upper and lower locations remained within ±0.06 °C, indicating vertically uniform performance. This suggests that, after engineering and scale-up guided by the optimized geometric parameters, the BSDAR mitigates mutual coupling between upper and lower reflective pathways on facades, providing a foundation for deployment on larger and taller building facades.

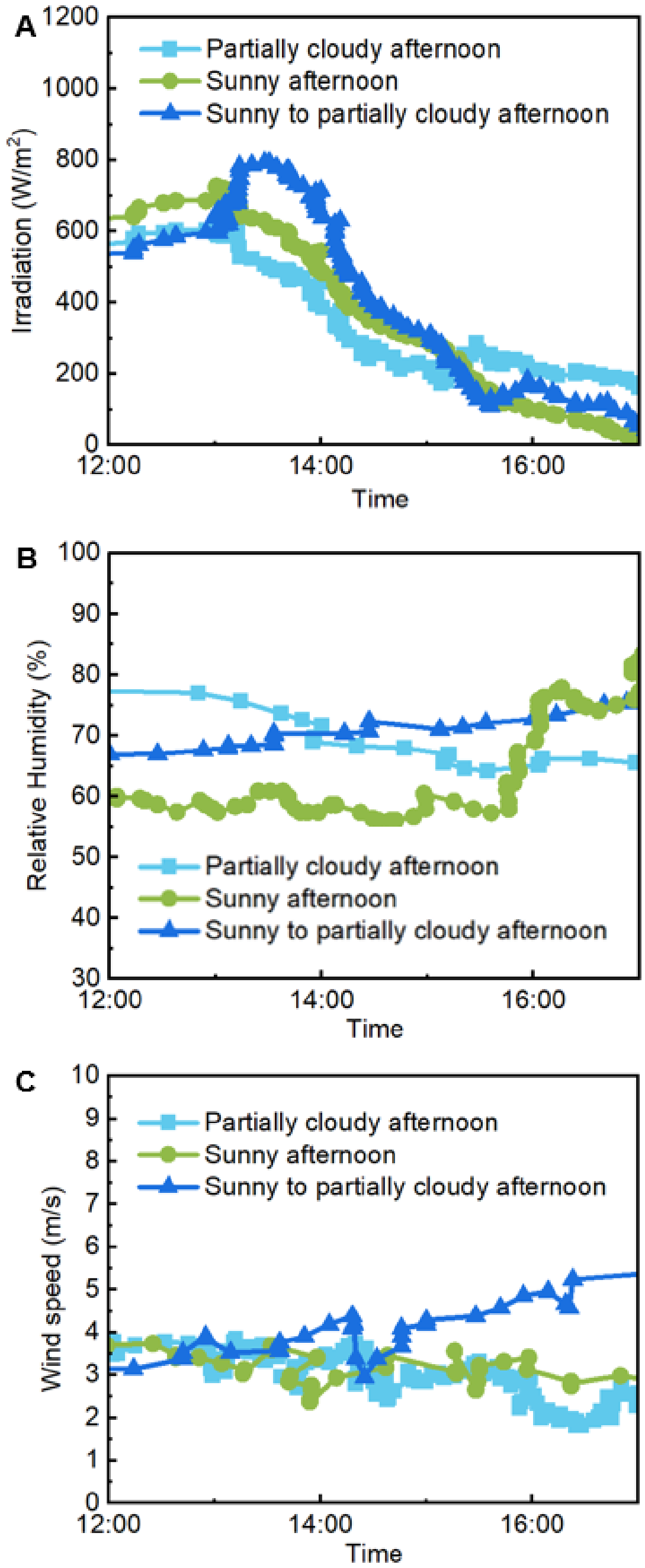

Figure S18. Detailed environmental light irradiation (A), relative humidity (B) and wind speed (C) during tests in Figure 3.

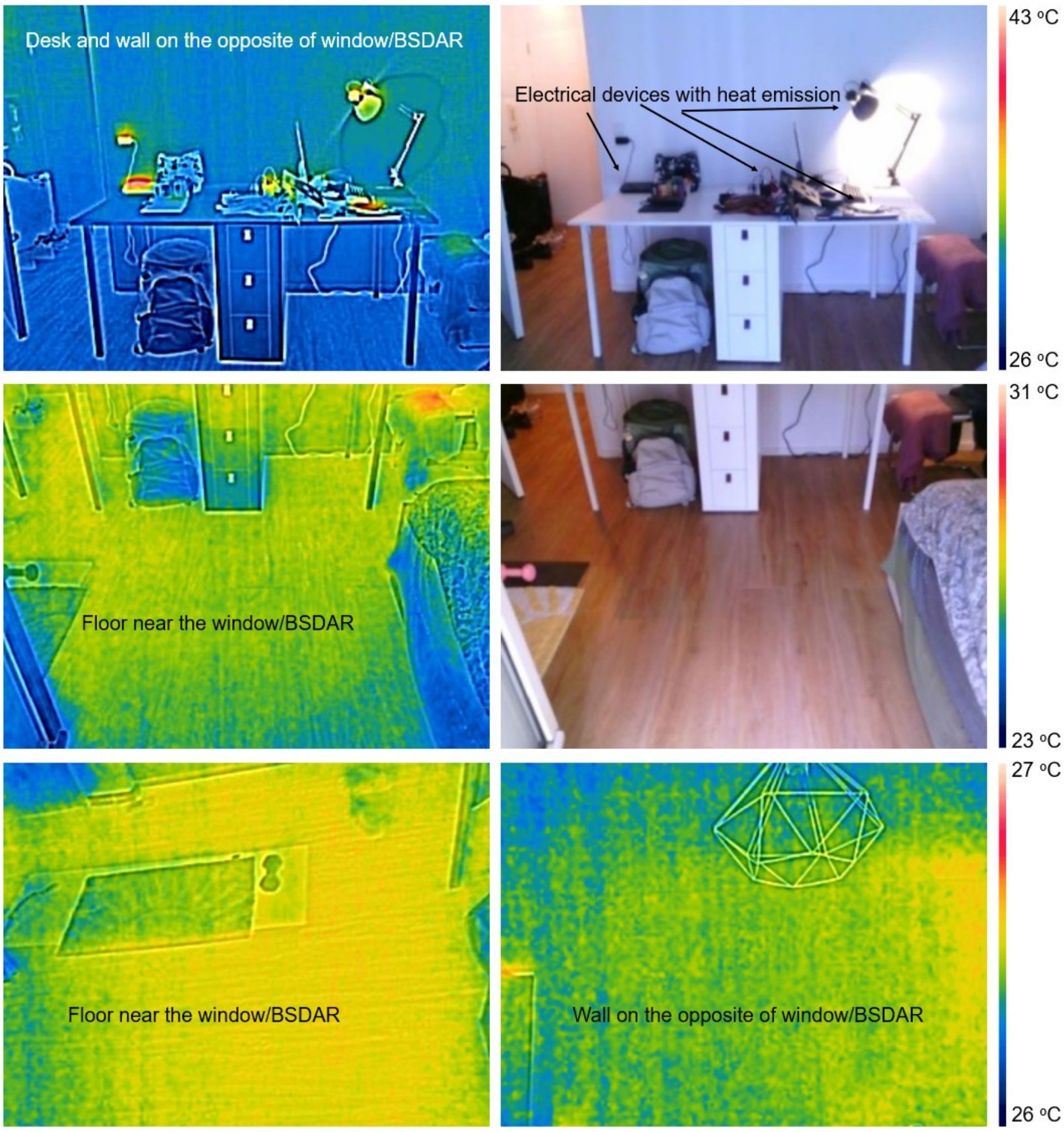

Figure S19. Thermal survey of a room fitted with a large-format BSDAR array. Infrared thermograms and matching visible photographs of the desk and opposite wall, the floor near the window (middle and bottom left) and the opposite wall (top), recorded on 25/07/2026 at 14:00 under sunny condition. Temperature scales differ between panels because the desk view contains electrical equipment operating well above room temperature, whereas the floor and wall views span a narrow range; each scale is chosen to resolve the detail of its own scene. Across the floor and wall surfaces, no localized hot spot attributable to redirected sunlight is observed.

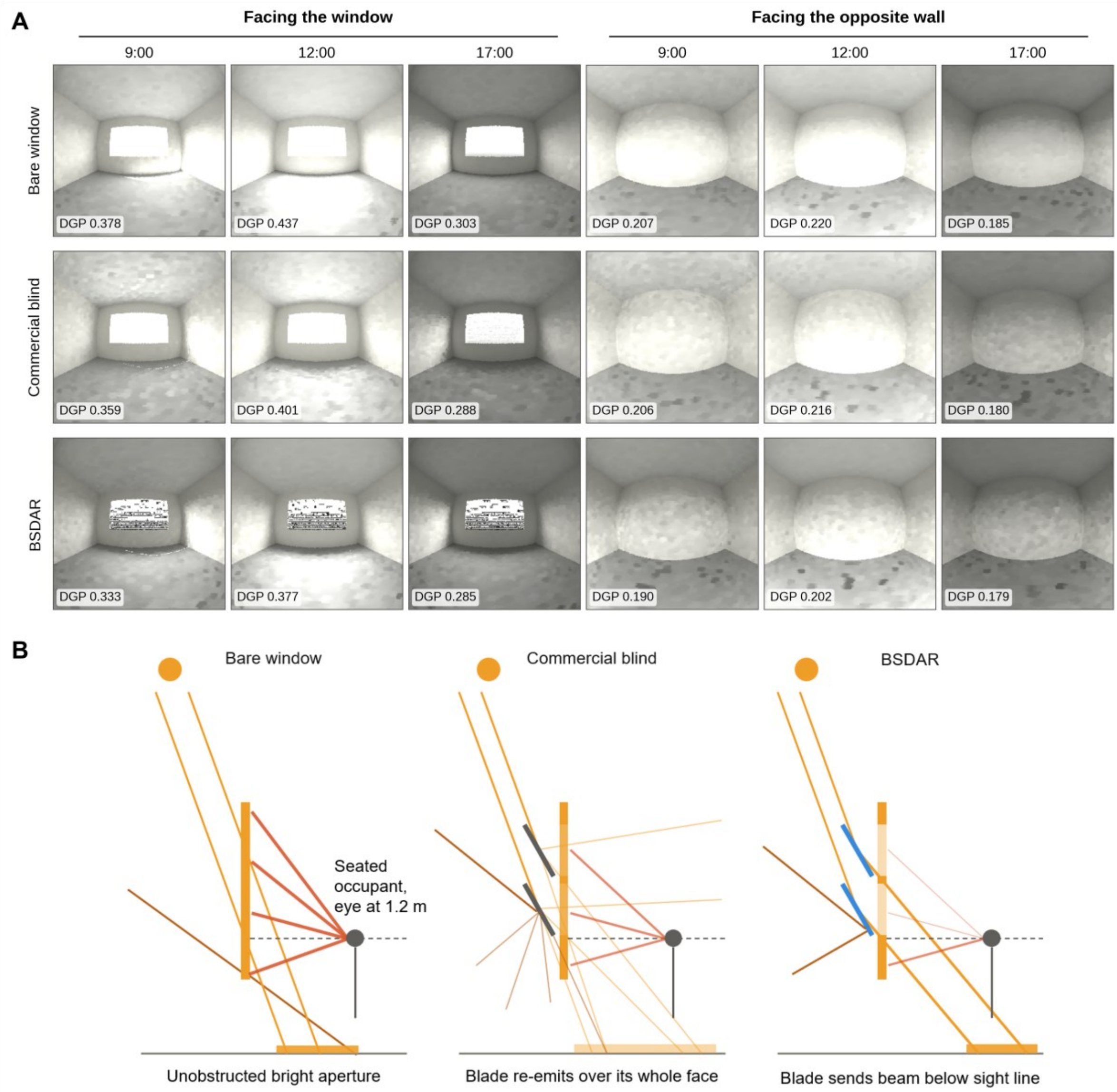

Figure S20. Simulated indoor luminance distributions and glare for a bare window, a commercial exterior venetian blind and BSDAR, and the geometric origin of the difference. (**A**) 180° fisheye renderings at seated eye height under TMYx local weather on 21 June at 9:00, 12:00 and 17:00, facing the window (left) and facing the opposite wall (right). Daylight Glare Probability (DGP, a metric predicting the fraction of occupants who would find a scene glaring, evaluated from the luminance of sources within the field of view weighted by their solid angle and position), evaluated with evalglare (the standard glare-evaluation tool of the Radiance suite, which identifies glare sources in a fisheye luminance image and returns the Daylight Glare Probability) from each rendering, is annotated in the lower left of each panel. The blind and BSDAR share identical slat geometry and differ only in slat optical properties. BSDAR gives the lowest DGP in every condition examined; the aperture appears as fine alternating luminance bands rather than a single broad bright surface. (**B**) Why a high

reflectance does not translate into more glare. Cross-sections of the same room with the sun at 70° altitude, drawn for slats inclined 30° from the vertical window plane and mounted outside the glazing. Amber rays are direct sunlight, which at this altitude reaches the upward-facing surface of a slat; brown rays are low-angle light reaching the outward-facing surface. The coloured strip on the glazing represents the luminance the seated occupant sees across the aperture, the bar on the floor marks where the intercepted sunlight lands, and red lines are the light reaching the eye. Glare is set by the luminance within the field of view, not by the quantity of light admitted. A bare aperture is bright over its whole height and fills the largest bright solid angle. A diffusely reflecting slat re-emits the intercepted sunlight over its entire surface, which lies within the field of view, and is therefore itself a luminance source irrespective of where the sun stands. A specular slat of the same reflectance receives the same radiation but assigns it a single outgoing direction: the sunlight leaves steeply onto the floor near the window, below the seated line of sight and onto a surface of reflectance 0.2, and the low-angle light is returned to the exterior. The reflectance determines how little is absorbed; the slat angle determines where the light goes. Ray paths are drawn from the law of reflection for the as-built geometry and are schematic; the DGP values are those of panel A.

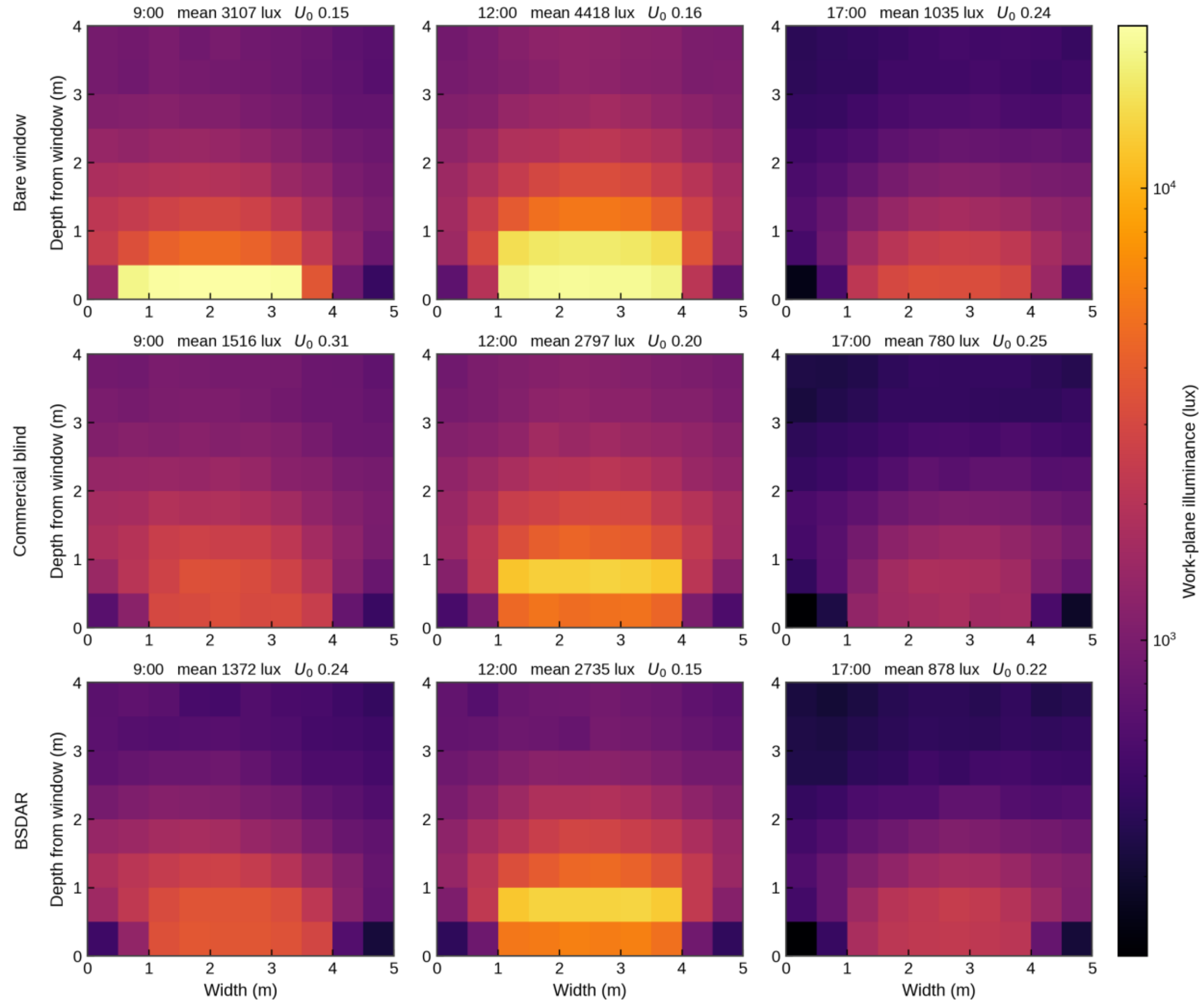

Figure S21. Simulated work-plane illuminance distributions. Illuminance on a 0.5 m grid at 0.75 m height for a bare window, a commercial exterior venetian blind and BSDAR, under TMYx local weather on 21 June at 9:00, 12:00 and 17:00. All panels share the logarithmic color scale at the right. Mean illuminance and uniformity U_0, defined as the ratio of minimum to mean over the grid, are given above each panel. The blind and BSDAR share identical slat geometry and differ only in slat optical properties. At solar noon the peak illuminance within 1 m of the window is 21359 lux for the bare window, 13729 lux for the blind and 13947 lux for BSDAR, so the specular and Lambertian devices differ by 1.6% and both reduce the near-window peak by about 35% relative to the bare case.

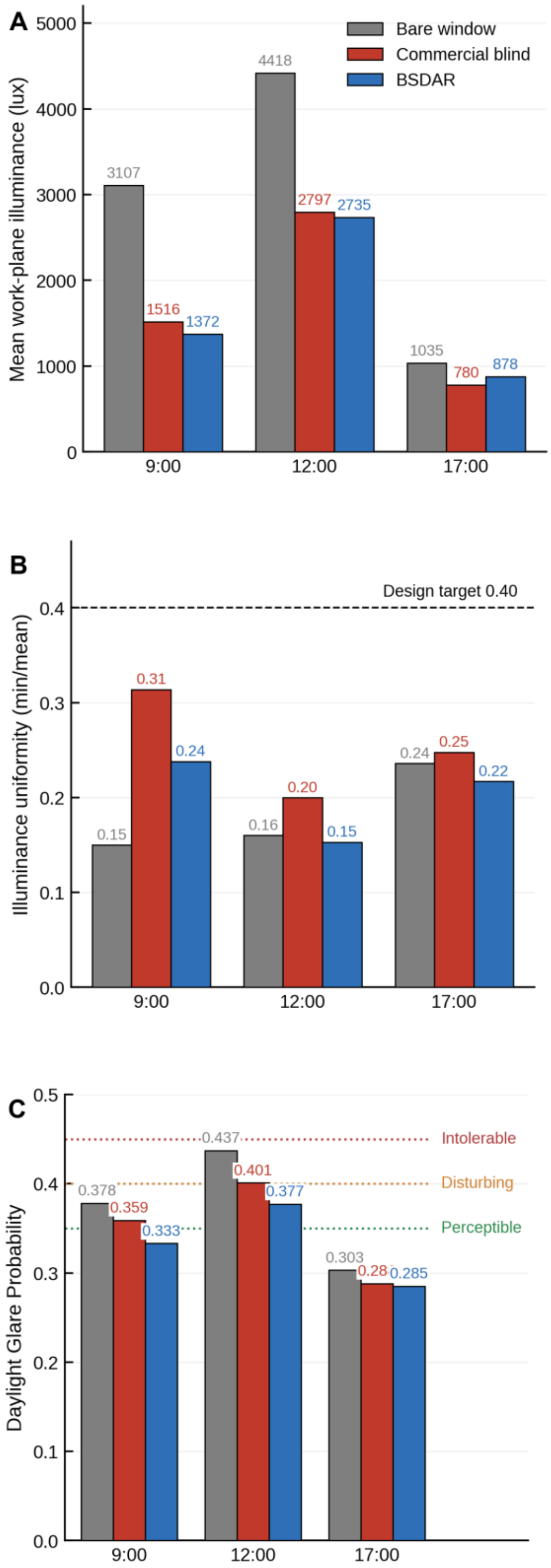

Figure S22. Daylighting and glare metrics. (**A**) Mean work-plane illuminance, (**B**) illuminance uniformity U_0 and (**C**) Daylight Glare Probability for a bare window, a commercial exterior venetian blind and BSDAR, under TMYx local weather on 21 June. Values are derived from the above distributions and the renderings. The dashed line in (B) marks the design target of 0.40; the dotted lines in (C) mark the perceptible, disturbing and intolerable thresholds. BSDAR gives the lowest DGP at every time examined while admitting daylight comparable to

the commercial blind; the blind achieves slightly higher uniformity, consistent with scattering distributing light more broadly with depth.

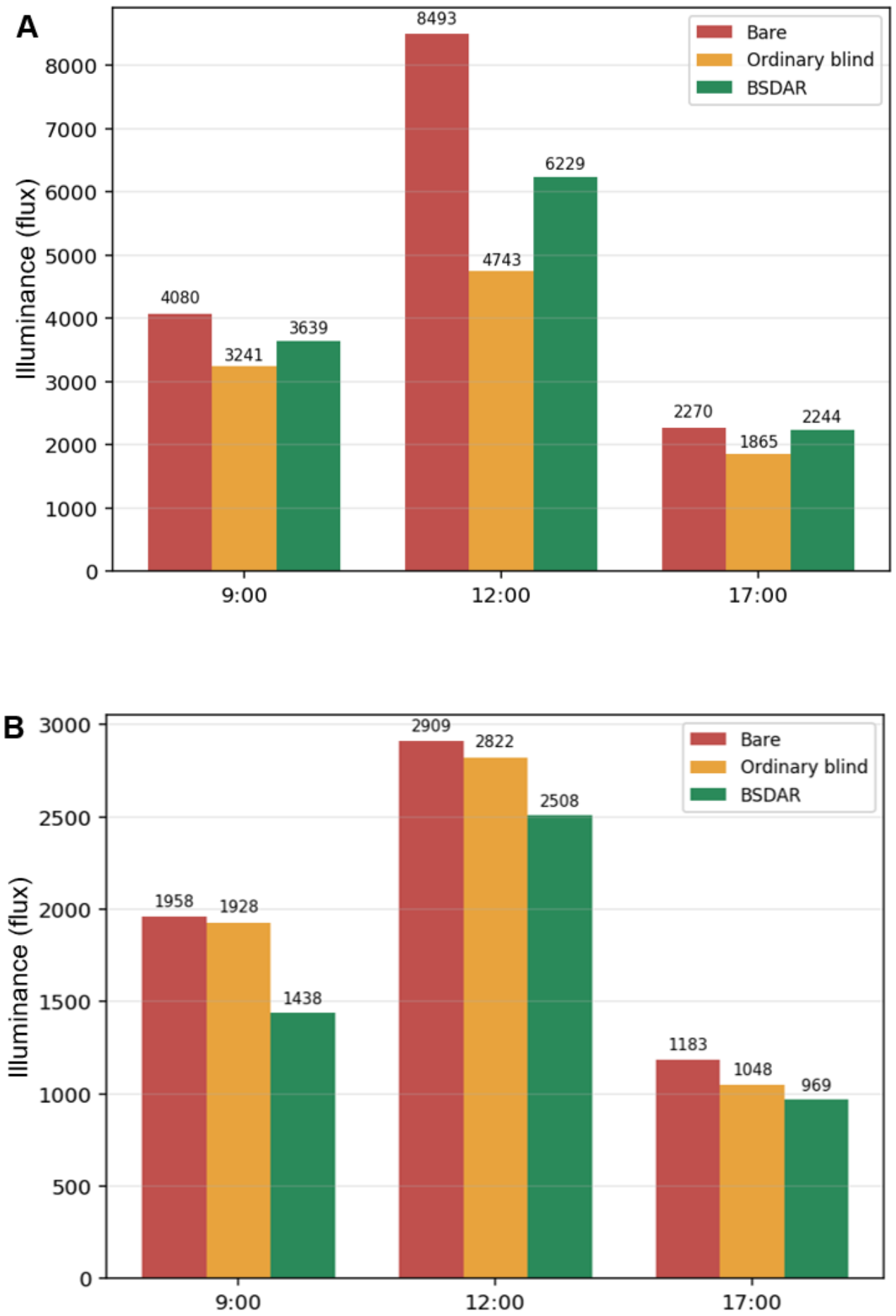

Figure S23. Testing interior illuminance at two depths on the room centerline. Illuminance at (**A**) 0.9 m and (**B**) 2.35 m from the south-facing window for a bare window, a commercial exterior venetian blind and BSDAR at 9:00, 12:00 and 17:00. At the near-window position the ordering of the three configurations matches that of the above simulated distributions; at 2.35 m the two shading devices give closely similar values and their relative order is not resolved. The ratio of far- to near-window illuminance is 0.40, 0.40 and 0.43 for BSDAR against 0.59, 0.59 and 0.56 for the commercial blind, indicating a steeper decay with depth for the specular device, consistent with the above work-plane uniformity (the ratio of the minimum to the mean illuminance over the evaluation grid).

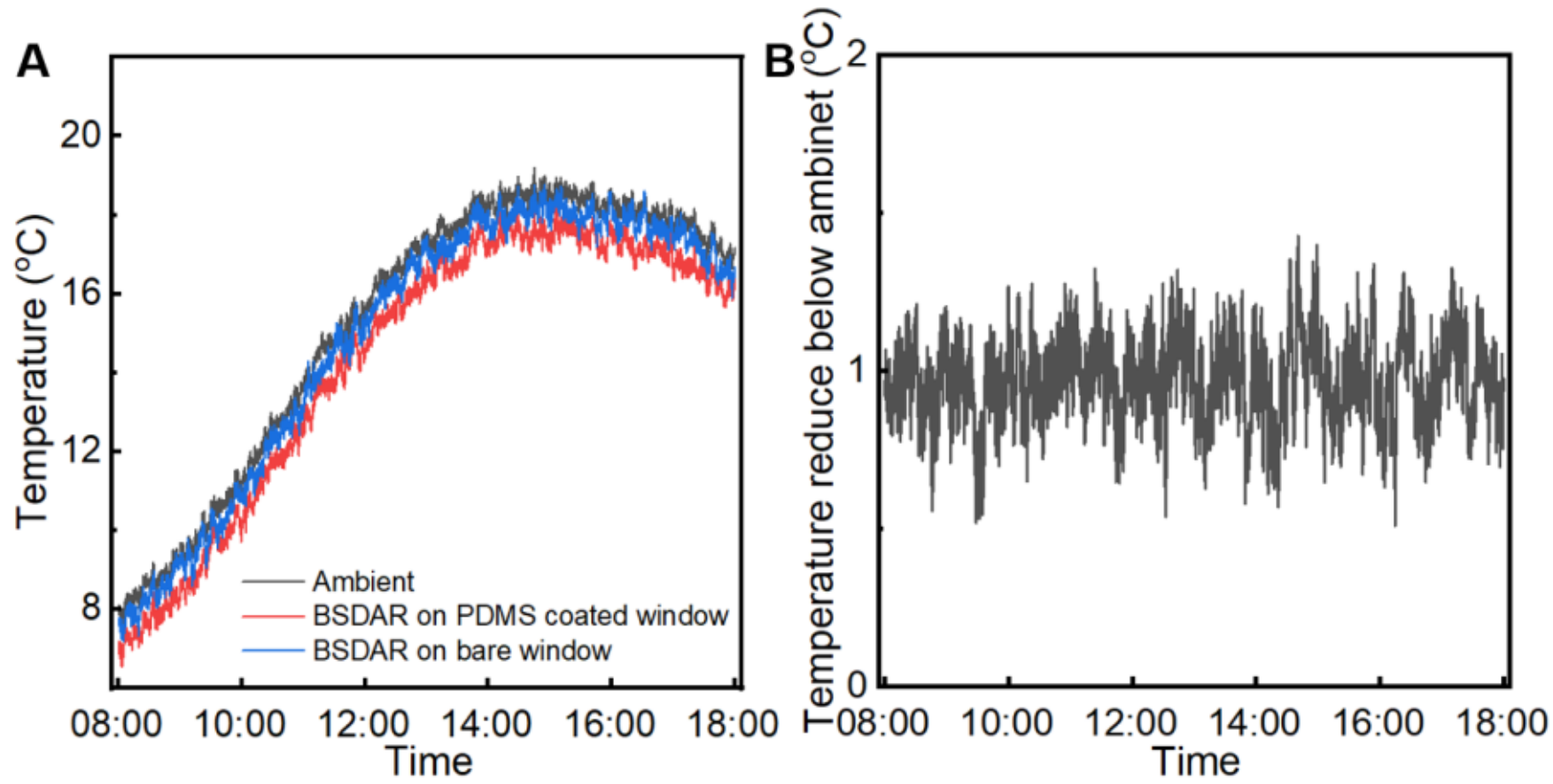

Figure S24. Sub-ambient cooling performance of the BSDAR system on real building windows facing to north under natural outdoor conditions. (**A**) Temperature vs. time curves for the north-facing, engineered BSDAR on real building window under sunny weather, without any wind-shielding or thermal-insulation setups. (**B**) Corresponding temperature reduce below ambient. The tested window location is in Eggenstein-Leopoldshafen, Germany (April, 7th, 2026). A 30 cm × 30 cm PDMS film, assembled by splicing, was applied to the outer surface of a standard building window pane, over which the BSDAR system was mounted; temperature was recorded by a sensor probe anchored directly onto the glass surface (labeled "BSDAR on PDMS coated window"). For comparison, the BSDAR system was also mounted on an uncoated bare window pane under otherwise identical conditions, with the glass temperature measured in the same manner (labeled "BSDAR on bare window"). Critically, unlike the earlier measurements designed to characterize the maximum radiative cooling potential, no polyethylene (PE) windshield films or additional thermal insulation were employed here to suppress ambient convective heat exchange. The results therefore reflect the practical, real-world cooling effect of the BSDAR system integrated into an operational building facade, demonstrating its capability to drive window surface temperatures below ambient air temperature without any convection-suppressing enclosure.

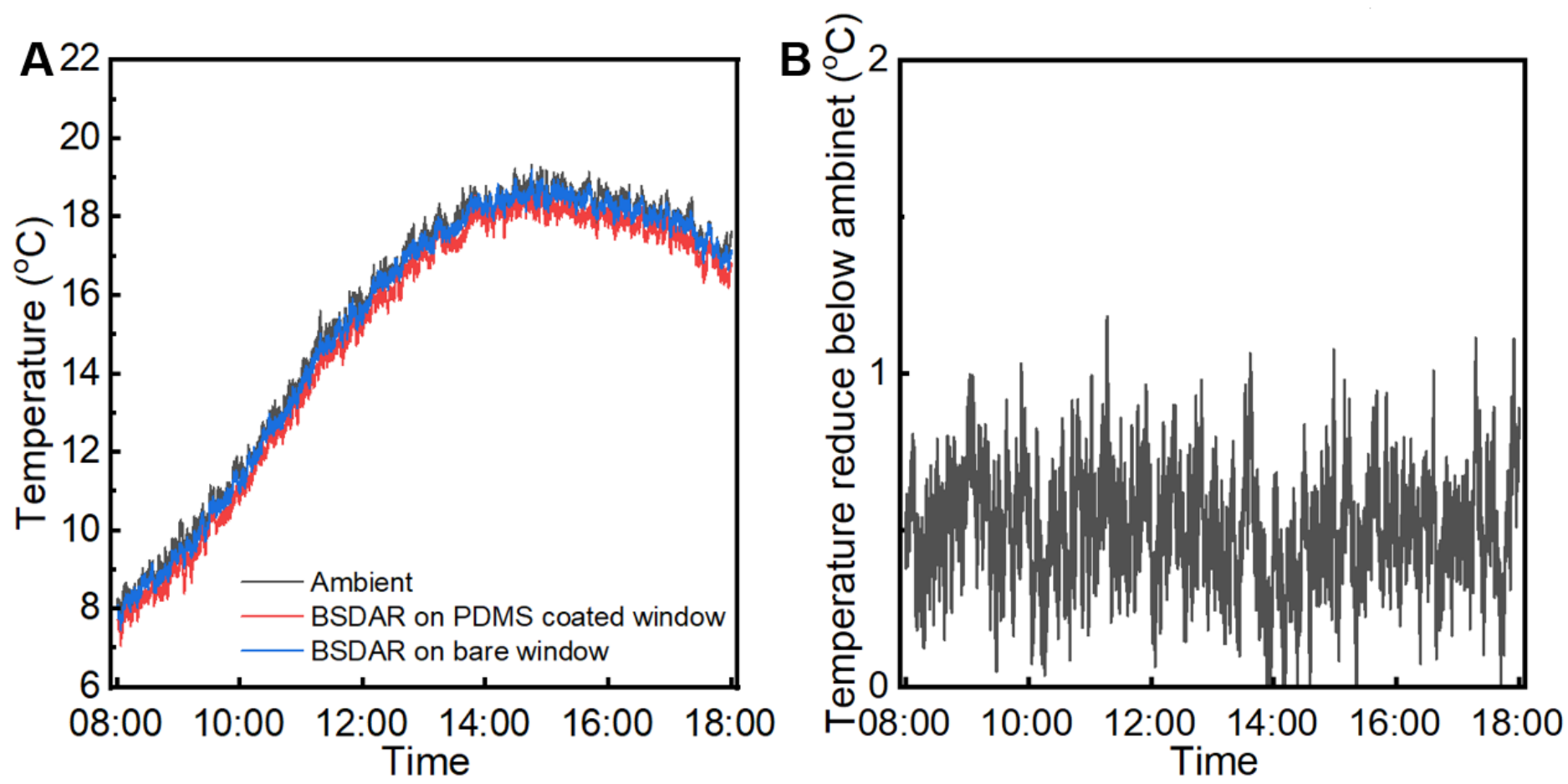

Figure S25. Sub-ambient cooling performance of the BSDAR system on real building windows facing to south under natural outdoor conditions. (**A**) Temperature vs. time curves for the south-facing, engineered BSDAR on real building window under sunny weather, without any wind-shielding or thermal-insulation setups. (**B**) Corresponding temperature reduce below ambient. The tested window location is in Eggenstein-Leopoldshafen, Germany (April, 7th, 2026). A 30 cm × 30 cm PDMS film, assembled by splicing, was applied to the outer surface of a standard building window pane, over which the BSDAR system was mounted; temperature was recorded by a sensor probe anchored directly onto the glass surface (labeled "BSDAR on PDMS coated window"). For comparison, the BSDAR system was also mounted on an uncoated bare window pane under otherwise identical conditions, with the glass temperature measured in the same manner (labeled "BSDAR on bare window"). Critically, unlike the earlier measurements designed to characterize the maximum radiative cooling potential, no polyethylene (PE) windshield films or additional thermal insulation were employed here to suppress ambient convective heat exchange. The results therefore reflect the practical, real-world cooling effect of the BSDAR system integrated into an operational building facade, demonstrating its capability to drive window surface temperatures below ambient air temperature without any convection-suppressing enclosure.

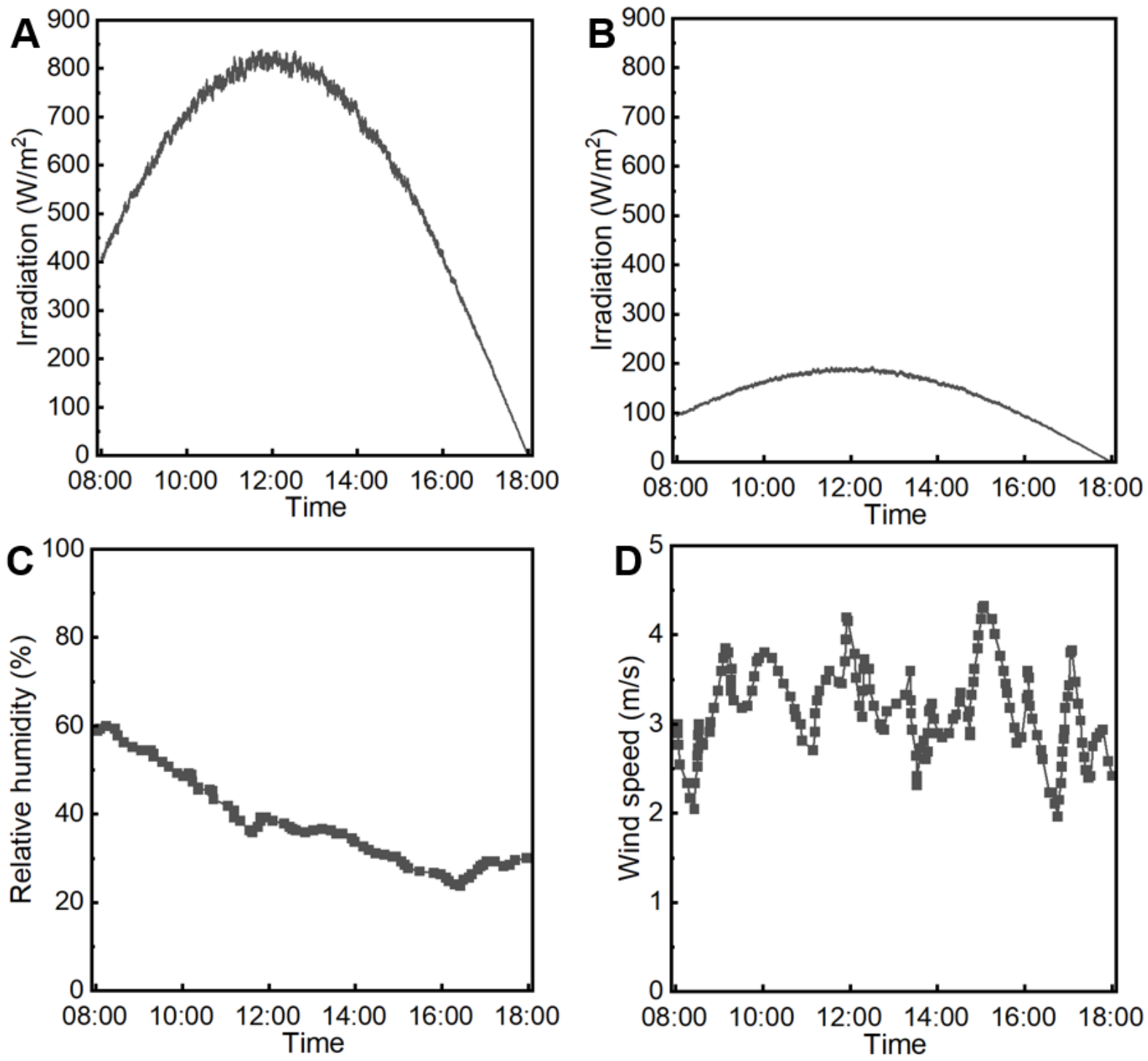

Figure S26. Detailed environmental light irradiation on building window facing to south (A), and facing to north (B), relative humidity (C) and wind speed (D) during tests in Figure S24 and S25.

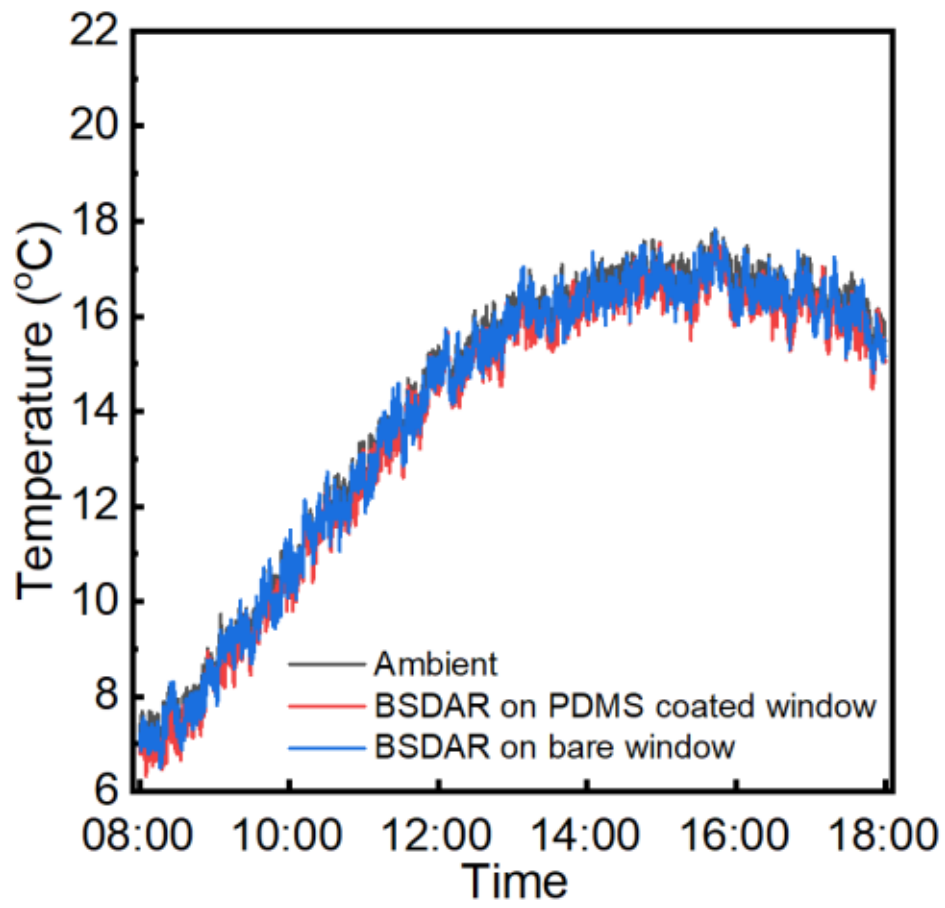

Figure S27. Sub-ambient cooling performance of the BSDAR system on real building windows facing to south under natural outdoor conditions. Temperature vs. time curves for the south-facing, engineered BSDAR on real building window under partially cloudy weather, without any wind-shielding or thermal-insulation setups. The tested window location is in Eggenstein-Leopoldshafen, Germany (April, 5th, 2026). A 30 cm × 30 cm PDMS film, assembled by splicing, was applied to the outer surface of a standard building window pane, over which the BSDAR system was mounted; temperature was recorded by a sensor probe anchored directly onto the glass surface (labeled “BSDAR on PDMS coated window”). For comparison, the BSDAR system was also mounted on an uncoated bare window pane under otherwise identical conditions, with the glass temperature measured in the same manner (labeled “BSDAR on bare window”). Critically, unlike the earlier measurements designed to characterize the maximum radiative cooling potential, no polyethylene (PE) windshield films or additional thermal insulation were employed here to suppress ambient convective heat exchange. The results therefore reflect the practical, real-world cooling effect of the BSDAR system integrated into an operational building facade, demonstrating its capability to drive window surface temperatures below ambient air temperature without any convection-suppressing enclosure.

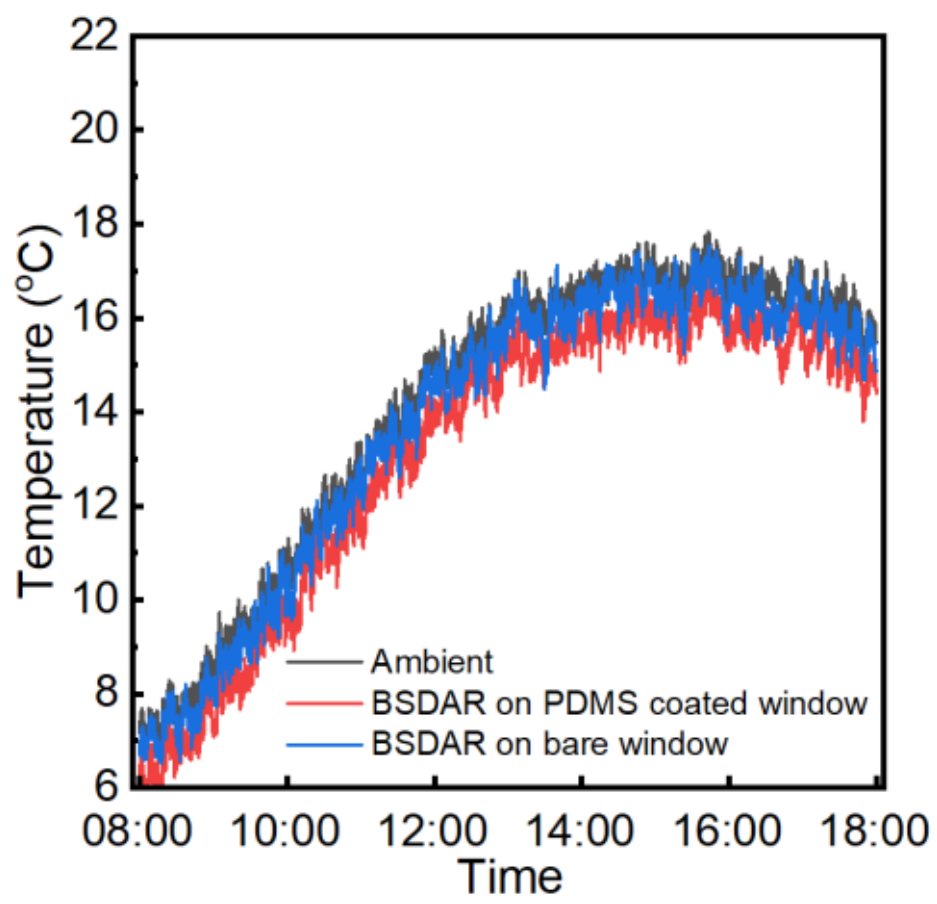

Figure S28. Sub-ambient cooling performance of the BSDAR system on real building windows facing to south under natural outdoor conditions. Temperature vs. time curves for the north-facing, engineered BSDAR on real building window under partially cloudy weather, without any wind-shielding or thermal-insulation setups. The tested window location is in Eggenstein-Leopoldshafen, Germany (April, 5th, 2026). A 30 cm × 30 cm PDMS film, assembled by splicing, was applied to the outer surface of a standard building window pane, over which the BSDAR system was mounted; temperature was recorded by a sensor probe anchored directly onto the glass surface (labeled "BSDAR on PDMS coated window"). For comparison, the BSDAR system was also mounted on an uncoated bare window pane under otherwise identical conditions, with the glass temperature measured in the same manner (labeled "BSDAR on bare window"). Critically, unlike the earlier measurements designed to characterize the maximum radiative cooling potential, no polyethylene (PE) windshield films or additional thermal insulation were employed here to suppress ambient convective heat exchange. The results therefore reflect the practical, real-world cooling effect of the BSDAR system integrated into an operational building facade, demonstrating its capability to drive window surface temperatures below ambient air temperature without any convection-suppressing enclosure.

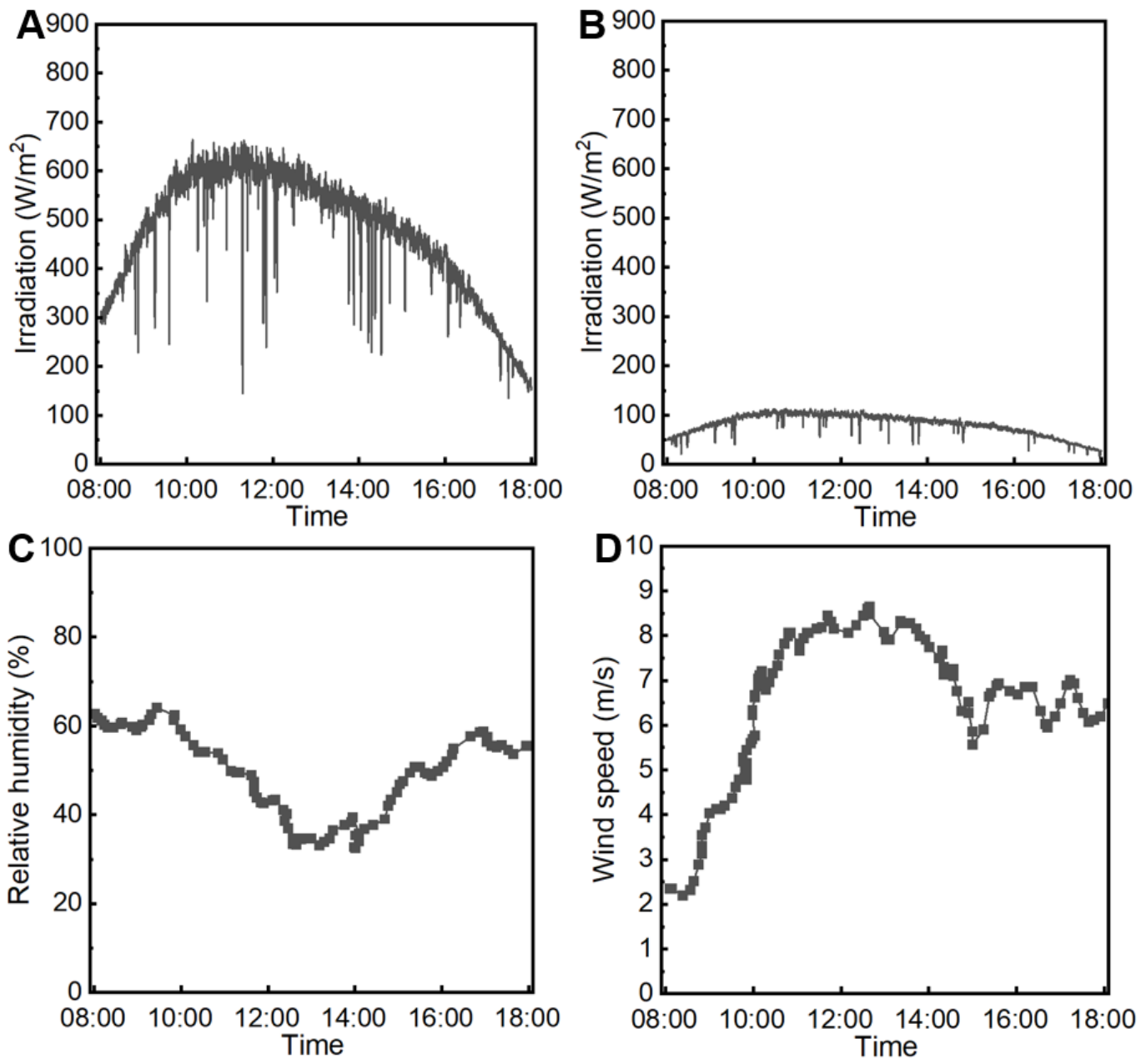

Figure S29. Detailed environmental light irradiation on building window facing to south (A), and facing to north (B), relative humidity (C) and wind speed (D) during tests in Figure S27 and S28.

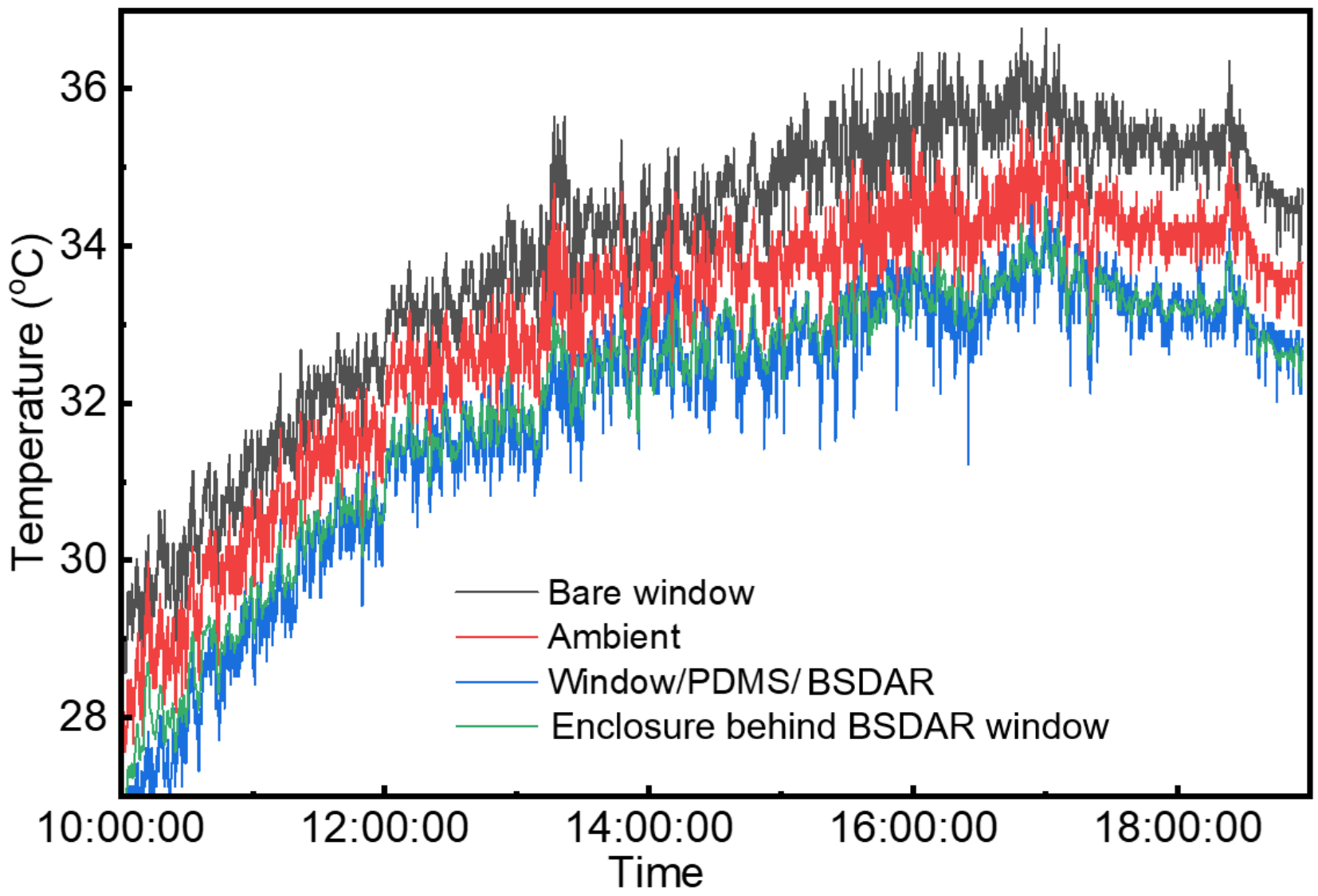

Figure S30. Sub-ambient cooling of the enlarged BSDAR on a south-facing building window under hot weather. Surface temperatures of a bare window and of a window fitted with PDMS and BSDAR, together with ambient air temperature and the temperature of a small enclosure mounted on the interior side of the window as a proxy for the near-window indoor environment. Measurements were made on an unconditioned window under purely passive operation, without any wind shield or thermal insulation, at ambient temperatures above 30 °C on 12 July 2026. Over the daytime period the BSDAR-covered window remains on average 0.907 °C below ambient. The corresponding weather conditions are given in Figure S31.

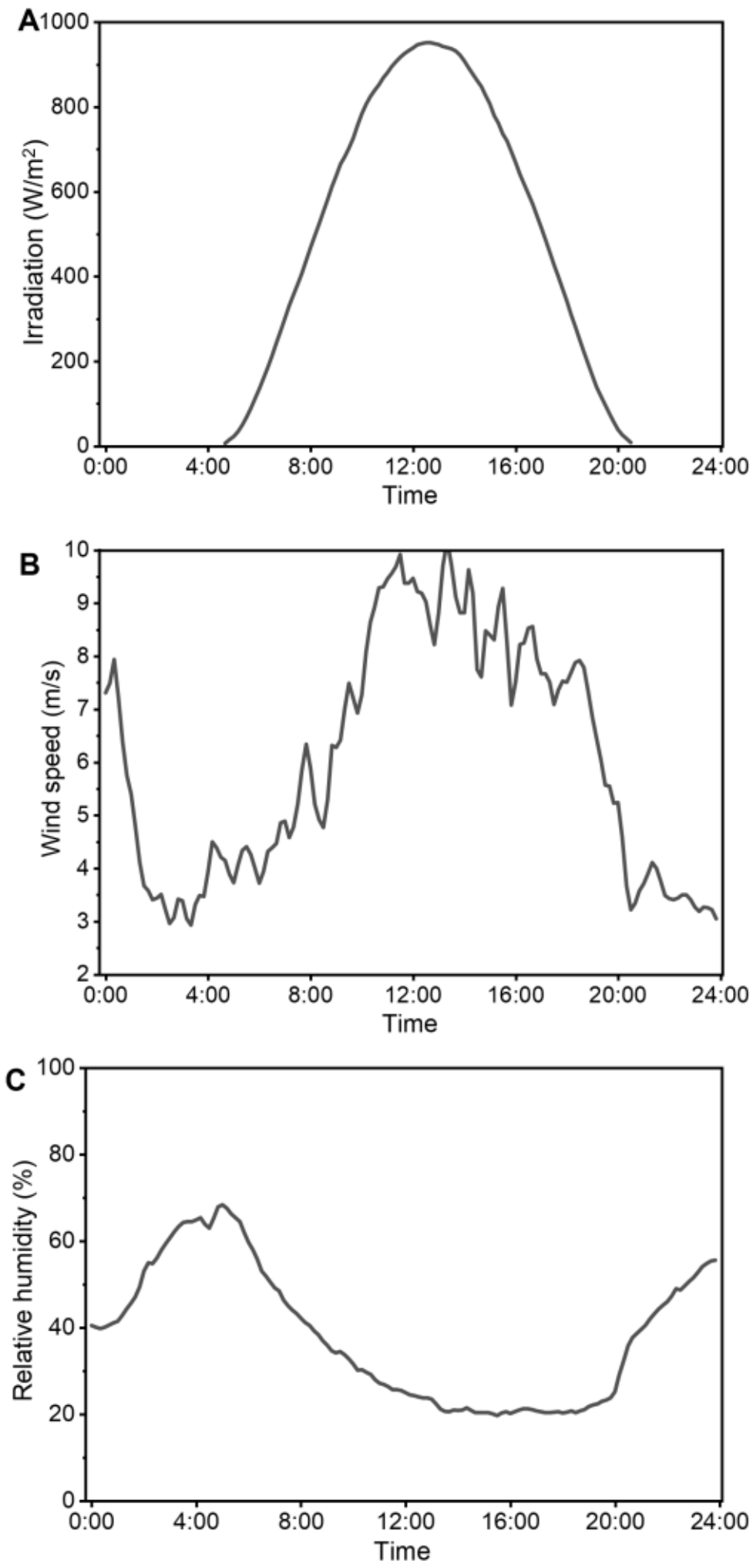

Figure S31. Weather conditions during the measurement of Figure S30. (**A**) Solar irradiance, (**B**) relative humidity and (**C**) wind speed recorded over the same period on 12 July 2026.

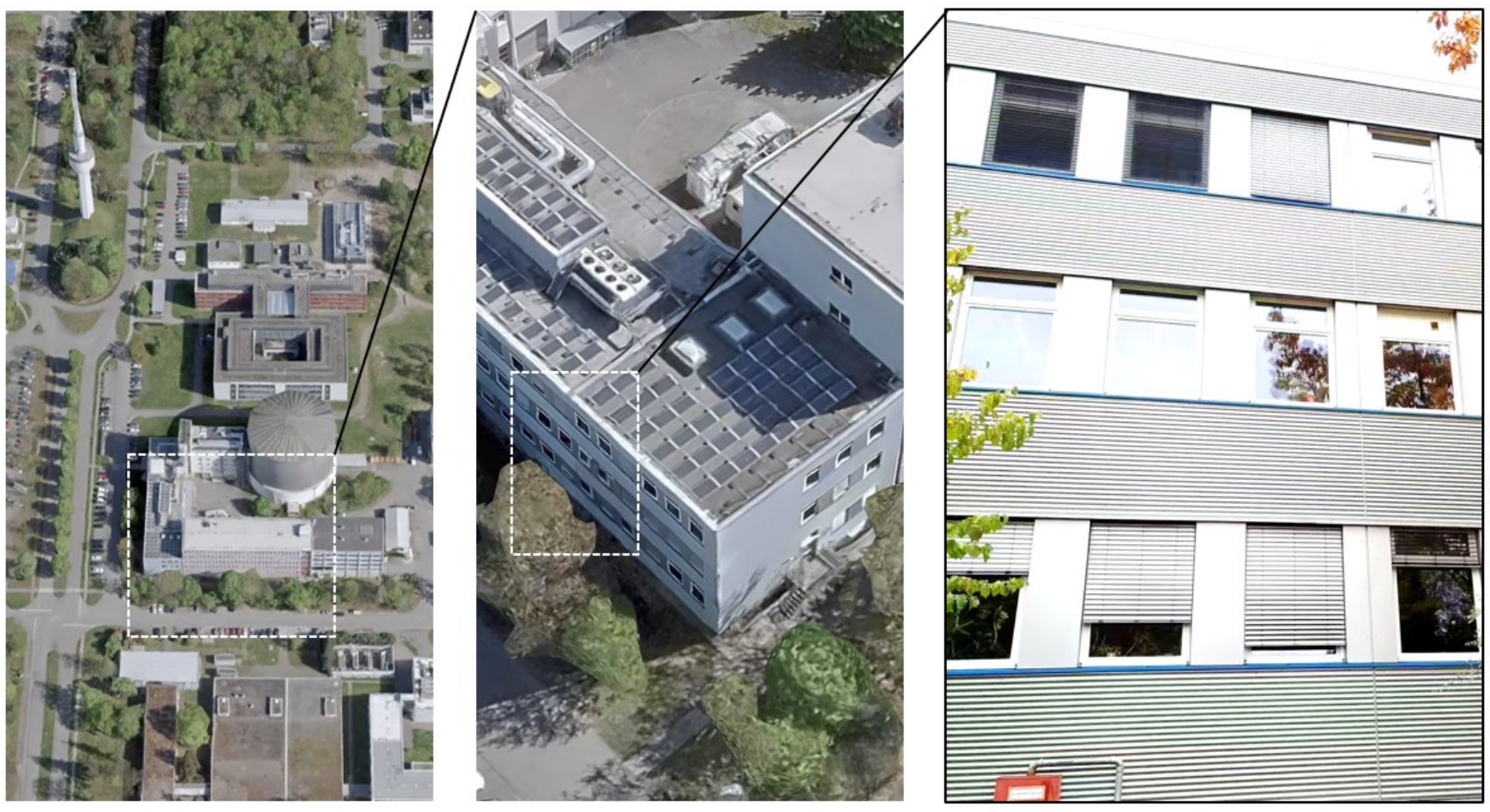

Figure S32. Reference building of the EnergyPlus model. It features with reflector arrays on both the windows. The building is located at Eggenstein-Leopoldshafen, Germany. Our EnergyPlus model is based on this existing building structure and replace all the reflector arrays to BSDAR arrays. Detailed model structure can be seen in Figure S33.

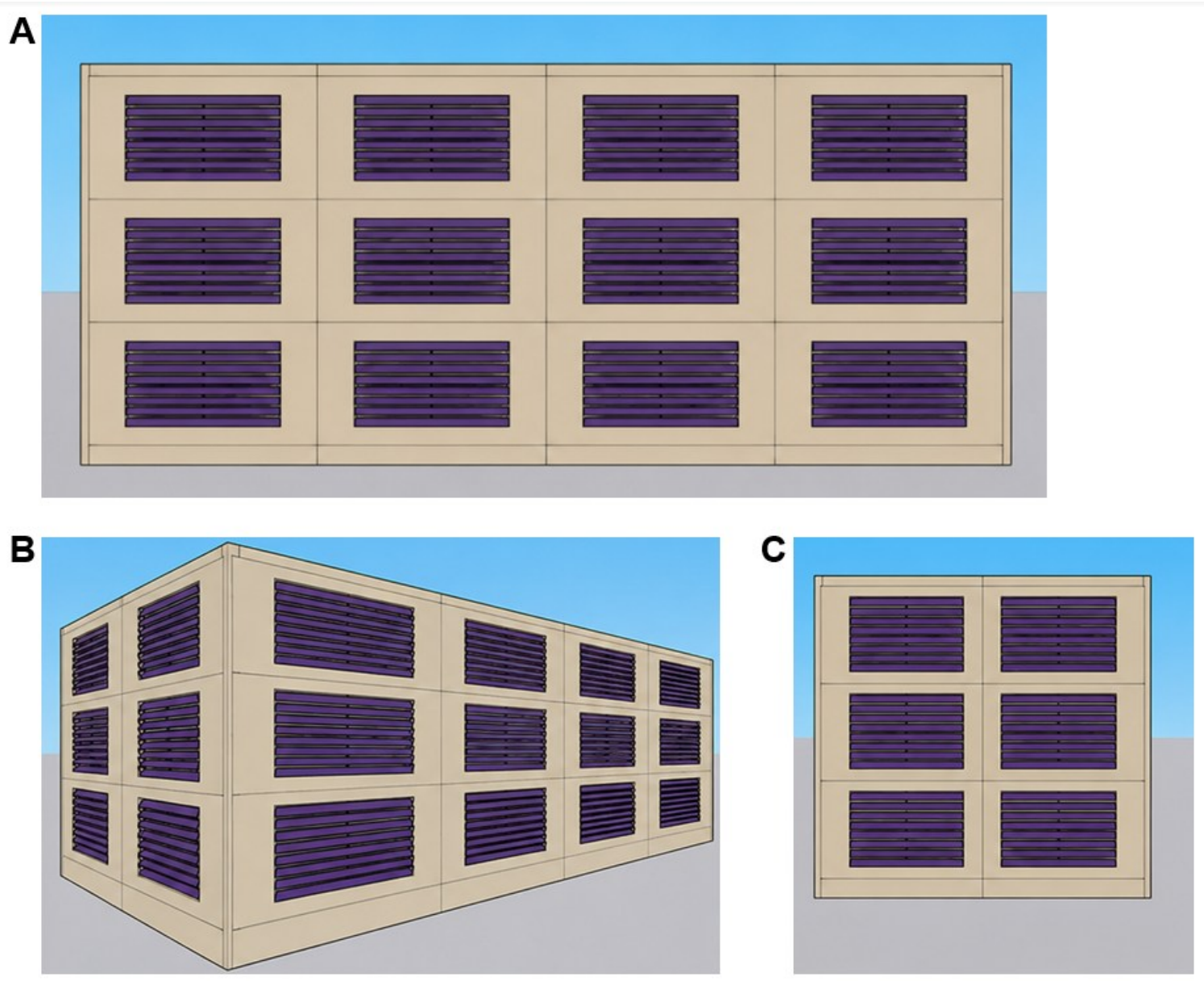

Figure S33. Detailed EnergyPlus simulation model structure. (**A**) Front view, (**B**) perspective view, (**C**) side view. More modeling and calculating details can be found in following supplementary text S2.

Supplementary Text

S2. Methods in energy saving calculations

Annual energy demand was simulated in EnergyPlus for a 55-zone office building of 5400 m² floor area with 80 windows, exported from Open Studio (Figure S33, S34). Three window configurations were compared under otherwise identical models: a bare window, a commercial exterior venetian blind, and BSDAR. Both shading devices were represented by the Window Material: Blind object (the standard EnergyPlus object representing a slat-type shading layer, defined by slat width, separation, thickness, angle and the solar and infrared optical properties of the slat surface) at the optimal geometry identified above, θ = 30° and β = 0.1, and differ only in slat optical properties. Those of BSDAR and the commercial blind were taken from the optical measurements reported here. Slat angle followed a seasonal schedule, held at θ = 30° through the hot season required cooling and, through the cold season required, held at θ = 90° during the day and closed (held at θ = 0°) at night. Simulations were run for a full year at hourly resolution using TMY and IWEC weather files, which represent a typical meteorological year constructed by selecting the most representative month from each year of a recent multi-year observational record at the nearest station, so that the resulting year is statistically typical rather than an actual calendar year, for representative cities on six continents.

The Window Material: Blind object evaluates slat reflection on a diffuse view-factor basis, so the directional character of each configuration was supplied separately. The BSDAR and commercial blind takes its slat optical properties based on the measurement (Figure 2 and Figure S54); the geometry being set to the as-built values common to the commercial blinds and BSDAR devices. Ray tracing was carried out in Radiance (a validated backward ray-tracing suite for lighting and daylighting simulation developed at Lawrence Berkeley National Laboratory) on the as-built geometry, following the thermal emission of the PDMS-coated glazing to termination and recording the fraction reaching the sky for BSDAR, for the bare window and for the commercial blind, with the balance distributed between the ground, the slats and return to the glazing. For the bare window the traced sky fraction agrees with the view factors EnergyPlus applies natively to a vertical surface, and for the commercial blind it is consistent with the default Window Material: Blind treatment; no correction is therefore required in either case. For BSDAR the diffuse treatment cannot represent specular redirection, and the traced fractions were used to assign a distinct directional radiative exchange, resolving solar energy absorbed on the slats, thermal radiation returned to the glazing, and radiation reaching the ground in addition to the sky-directed component. The resulting changes in the exchange coefficients toward the sky and toward the ground, Δc_{sky} and Δc_{gnd}, were combined

with live sensor readings of ambient and effective sky temperature and applied as an equivalent load through an Other Equipment object (an EnergyPlus object that injects a user-defined heat gain, positive or negative, into a zone or surface without consuming any energy or fuel) using the Energy Management System (the built-in runtime scripting facility of EnergyPlus, which reads sensor values at every timestep and writes actuator values back into the simulation), for the BSDAR case only.

The model resolves the path from the glazing surface to the cooling energy in three stages (Figure S35). At the glazing, a surface energy balance combines absorbed solar radiation, infrared exchange with sky and ground including the redirection term above, external convection and conduction into the room; the glazing temperature is the solution of this balance and is therefore an intermediate quantity rather than a prescribed input. That temperature drives conduction through the window, which together with the transmitted solar component forms the window heat flux entering the zone. The zone air heat balance then combines this flux with internal, envelope and infiltration gains over all 55 zones, giving the free-floating (a run with all conditioning disabled, so that the indoor air temperature is determined solely by the zone heat balance) indoor air temperature when no HVAC is present and the cooling and heating load when the zone is held at setpoint.

Cooling and heating demand were extracted from the monthly District Cooling and District Heating Water meters (the EnergyPlus output meters recording the thermal energy delivered to and removed from the conditioned zones), verified against zone-level Ideal Loads Supply Air output (an idealized air system that meets the zone setpoint exactly, so that the reported quantity is the zone load itself rather than the performance of a particular plant). Results are reported as energy intensity normalized to floor area, as free-floating indoor temperature, and as annual cost saving obtained by dividing by the system coefficient of performance and multiplying by local tariff.

The lowest measured sub-ambient surface temperature was not used as a model input. The simulations are driven by the measured optical properties, the array geometry and the weather files alone; the measured depression was used only as an independent check that the simulated façade temperatures are not overstated.

The solar radiation absorbed on the slats was resolved explicitly rather than neglected. The slat solar absorptance was taken from the measured spectra, and the absorbed flux was partitioned according to the surface energy balance of the slat: convection to the outdoor air on both faces, conduction to the glazing across the 8 mm gap, and long-wave radiation to the sky, to the ground and to the glazing. Over the summer this amounts to 5.1, 8.8 and 8.7 GJ for Guangzhou,

Athens and Phoenix respectively. Of that absorbed heat, 85.8% is removed by convection to the outdoor air, 9.7% conducts to the glazing, and 4.5% is radiated, of which 2.2% reaches the glazing; 11.9% therefore arrives at the window in total.

The consequence for cooling demand was obtained by running the model twice, once with the measured slat absorptance and once with a non-absorbing slat of the same geometry and infrared properties. The difference is 0.12%, 0.27% and 0.16% of the summer cooling demand in the three cities, and in each case the demand is lower with the absorbing slat. This follows from where the absorption occurs. Ray tracing of the incident beam shows that 96.4% reaches the glazing with a non-absorbing slat against 90.7% with the measured absorptance, so the radiation removed from the beam would otherwise have arrived at the window almost in full; once absorbed, only 11.9% of it returns there, the remainder being carried away to the outdoor air. A slat that absorbs a small fraction of the incident sunlight therefore acts as an intermediate heat sink placed outside the glazing rather than as a parasitic load upon it.

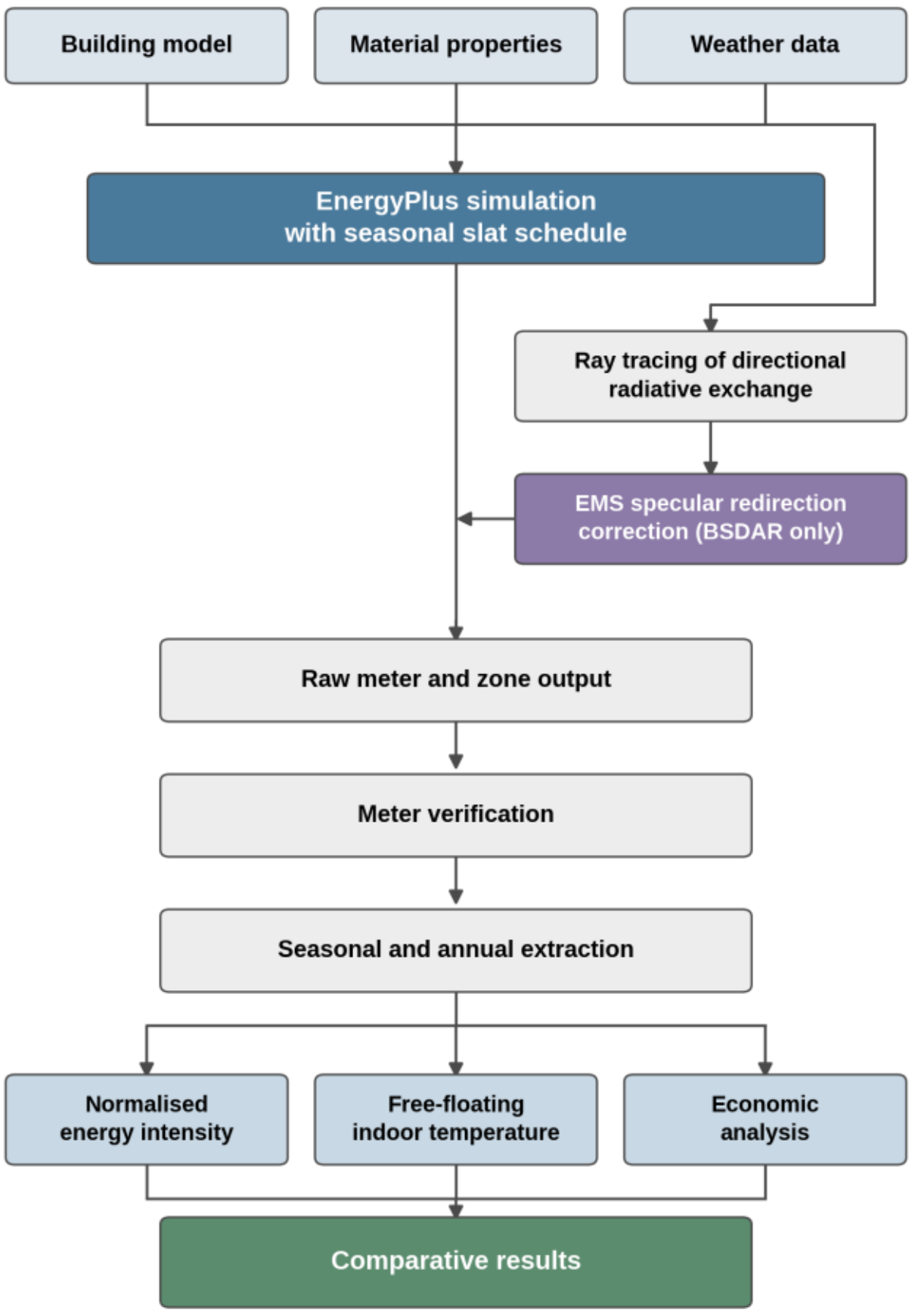

Figure S34. Workflow of the building energy simulation. Building geometry, measured material properties and real weather data drive an annual EnergyPlus simulation with the seasonal slat schedule. Because the Window Material: Blind object evaluates slat reflection on a diffuse view-factor basis, the directional radiative exchange was obtained separately by ray tracing for all three configurations and applied as a correction through the Energy Management System for BSDAR only; for the bare window and the commercial blind the traced transport agrees with the native treatment, so no correction is required. Meter output is verified against zone-level Ideal Loads Supply Air results before seasonal and annual extraction, and the verified values are reported as normalized energy intensity, free-floating indoor temperature and annual cost saving.

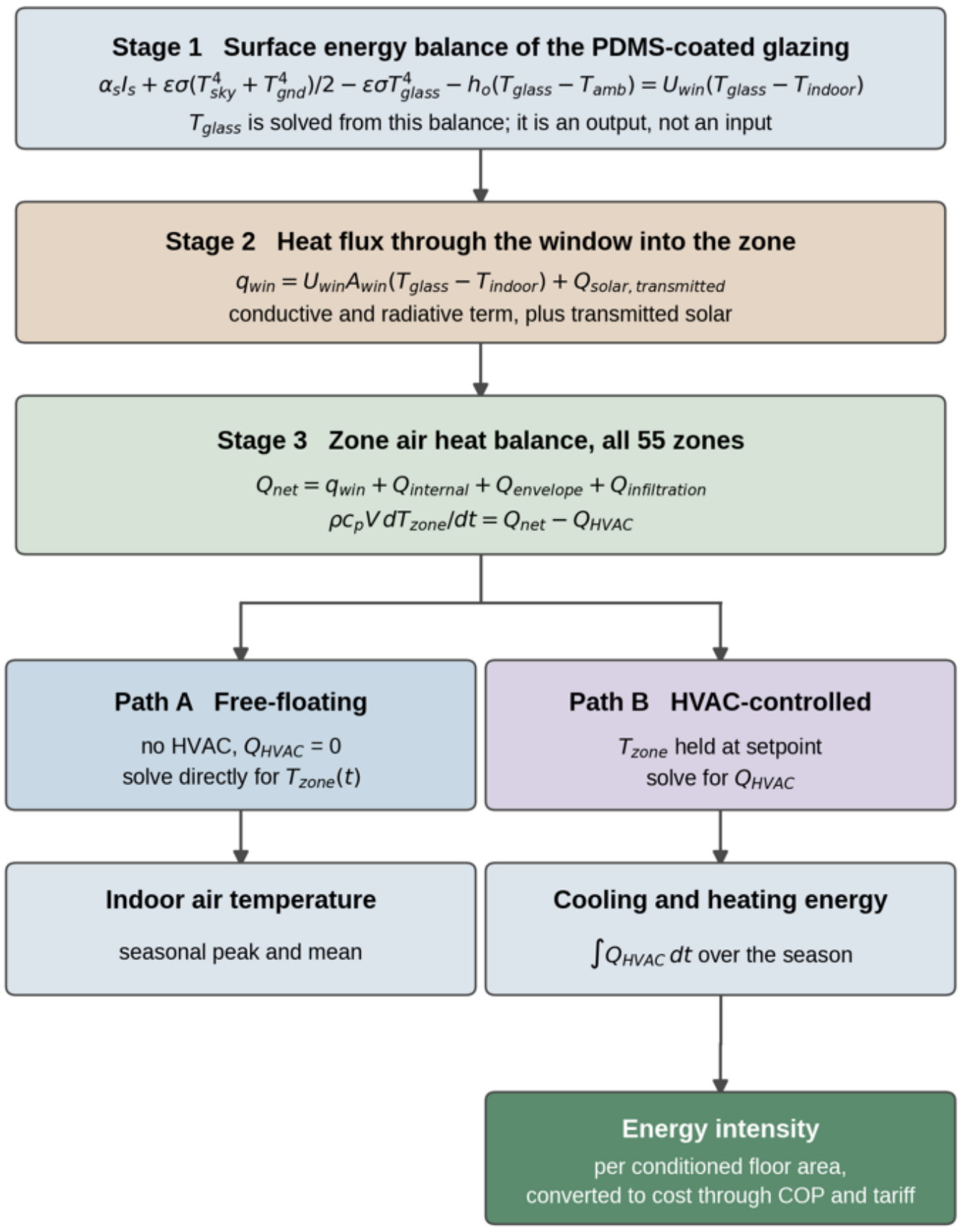

Figure S35. Heat-transfer chain linking the glazing surface to the cooling energy. BSDAR enters the model at the surface energy balance of the glazing, through its solar absorptance α_s, its infrared emissivity ε and the directional redirection term (Stage 1); the glazing temperature T_{glass} is solved from this balance and is therefore an intermediate output rather than an input. This temperature drives conduction through the window, which together with the transmitted solar component forms the window heat flux into the zone (Stage 2). The zone air heat balance over all 55 zones, combining this flux with internal, envelope and infiltration gains, then yields either the free-floating indoor temperature when no HVAC is present (Path A) or the cooling and heating load when the zone is held at setpoint (Path B), the latter being converted to energy intensity and cost (Stage 3).

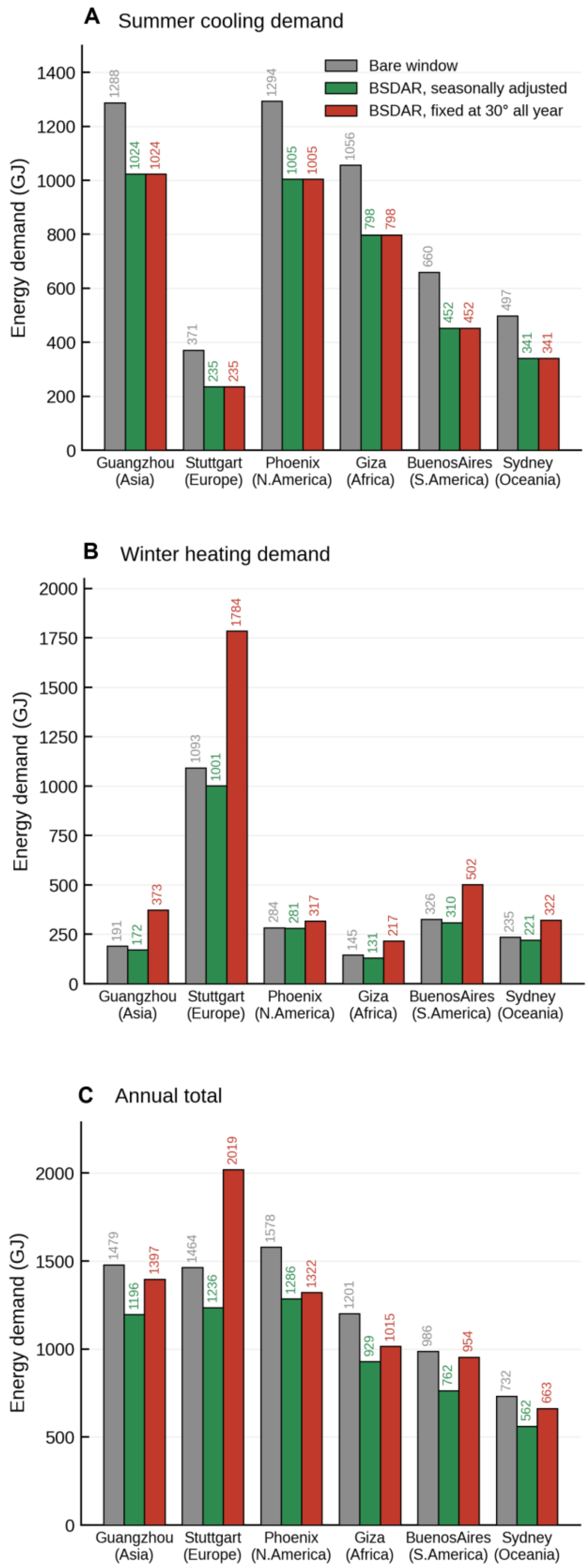

Figure S36. Annual energy balance of the seasonal adjustment strategy. (**A**) Summer cooling demand, (**B**) winter heating demand and (**C**) annual total for a bare window, BSDAR under the seasonal adjustment strategy, and BSDAR held at 30° throughout the year, for six

cities on six continents. All results are from EnergyPlus with the array mounted on the windows only. Summer values are identical for the two BSDAR cases, since both are at 30° during the hot season required cooling. Holding the array at 30° through the heating season blocks most of the low-angle winter sun and maintains the radiative cooling that is unwanted in winter, raising heating demand by 12 to 95%; in 49ºN (Karlsruhe area), where heating dominates the annual load, this outweighs the summer benefit and leaves the annual total 38% above that of a bare window. The seasonal strategy instead reduces heating demand by 1.1 to 9.9% while retaining the full summer benefit, giving an annual reduction of 15.6 to 23.2% in every city examined.

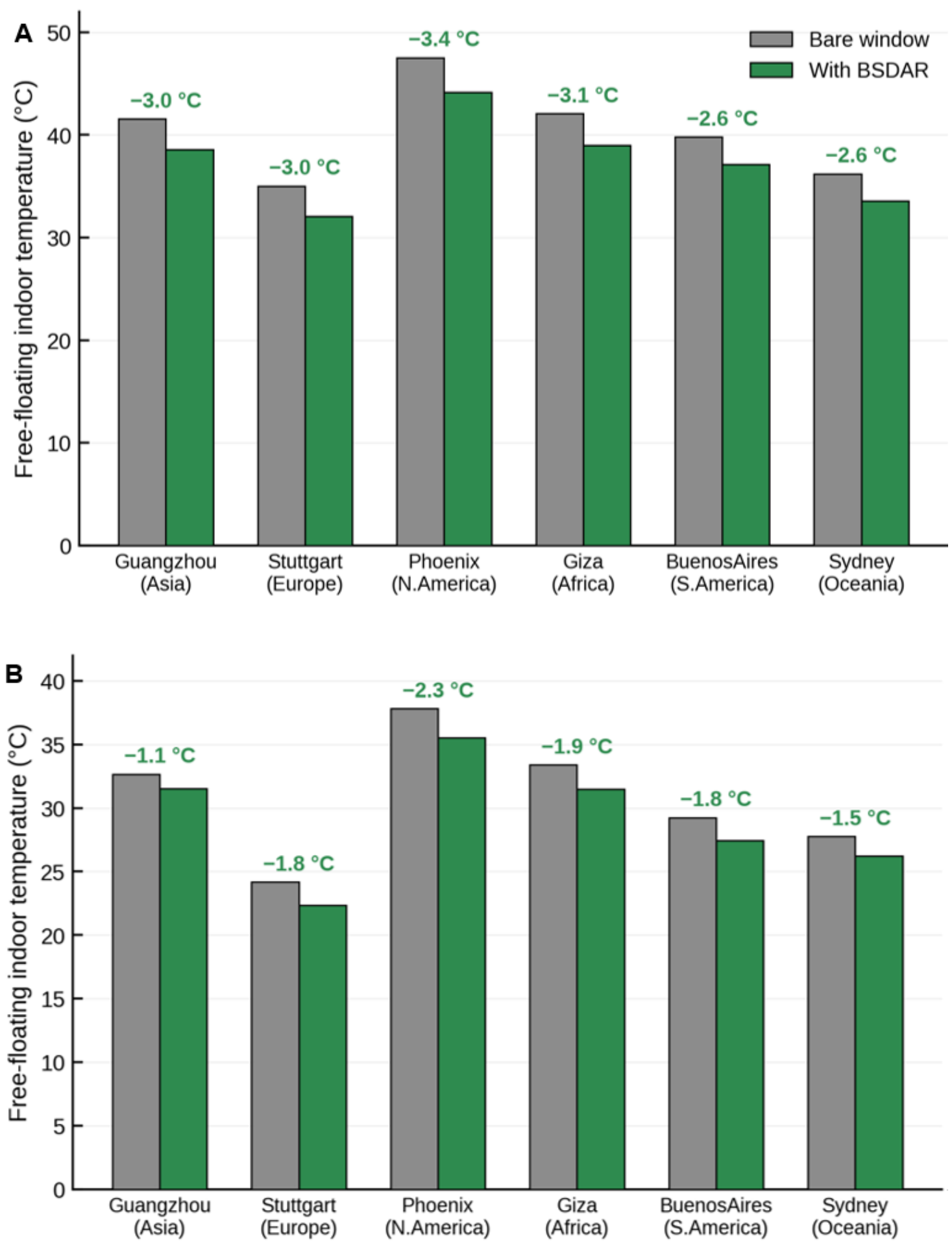

Figure S37. Free-floating indoor air temperature with and without BSDAR in summer. Simulations were repeated with all cooling disabled, so that the indoor air temperature floats freely under otherwise identical conditions. (**A**) Summer peak and (**B**) summer seasonal mean indoor air temperature for a bare window and for a window fitted with BSDAR, in six cities on six continents; December–February is taken as summer for the southern-hemisphere sites. Labels give the reduction achieved by BSDAR.

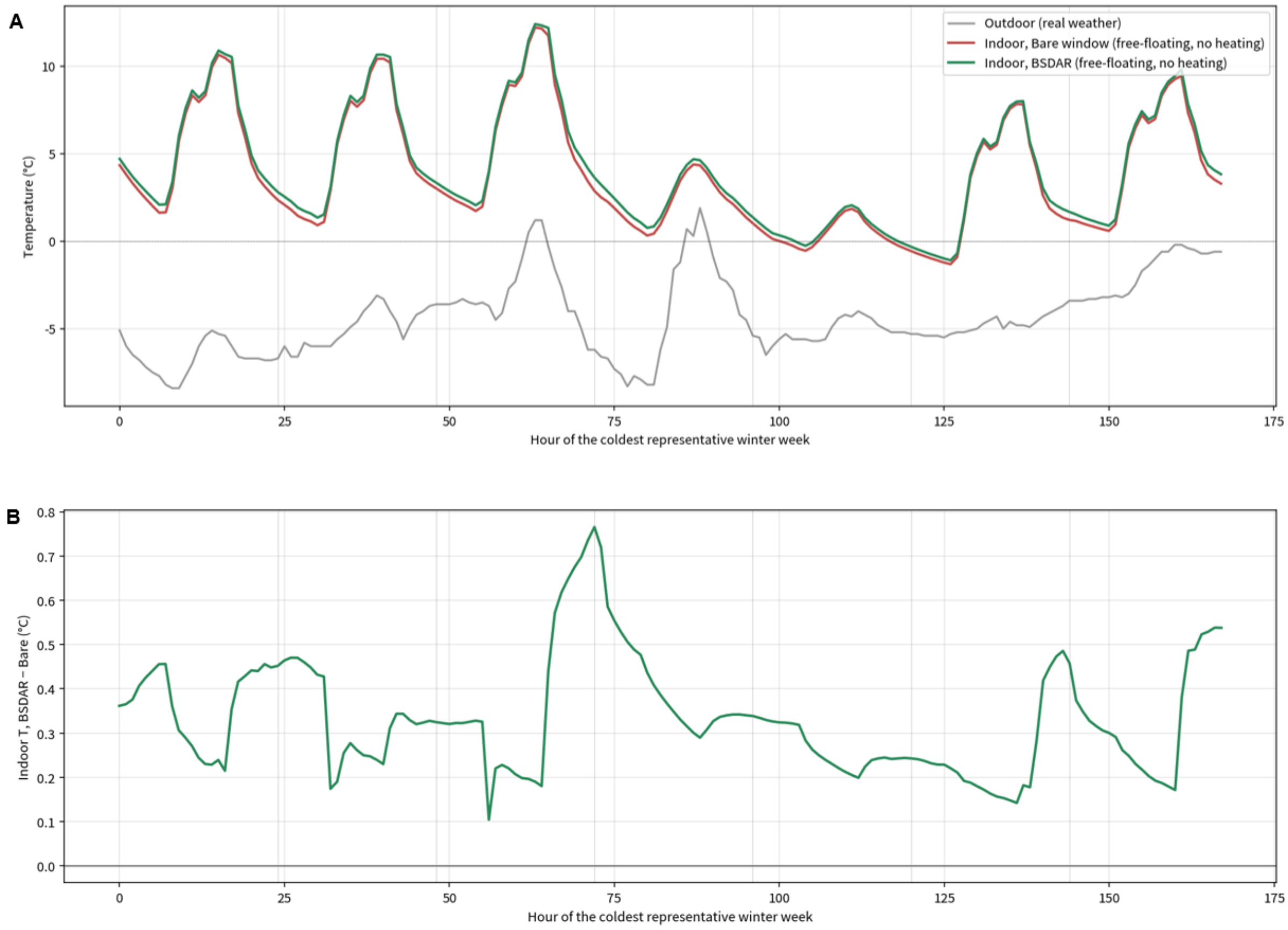

Figure S38. Free-floating indoor temperature over the coldest representative week. Simulated with all heating disabled so that the indoor temperature floats freely; the week shown is the coldest representative week of the weather file at the latitude of our field measurements, 49°N (Karlsruhe area). (**A**) Hourly indoor air temperature with a bare window and with BSDAR under the seasonal adjustment strategy, together with the outdoor air temperature. (**B**) The difference between the two, BSDAR minus bare window. The difference remains positive in every hour of the week, ranging from 0.1 to 0.77 °C; the residual advantage during the day, when the array is retracted, is thermal-mass carry-over from the preceding night.

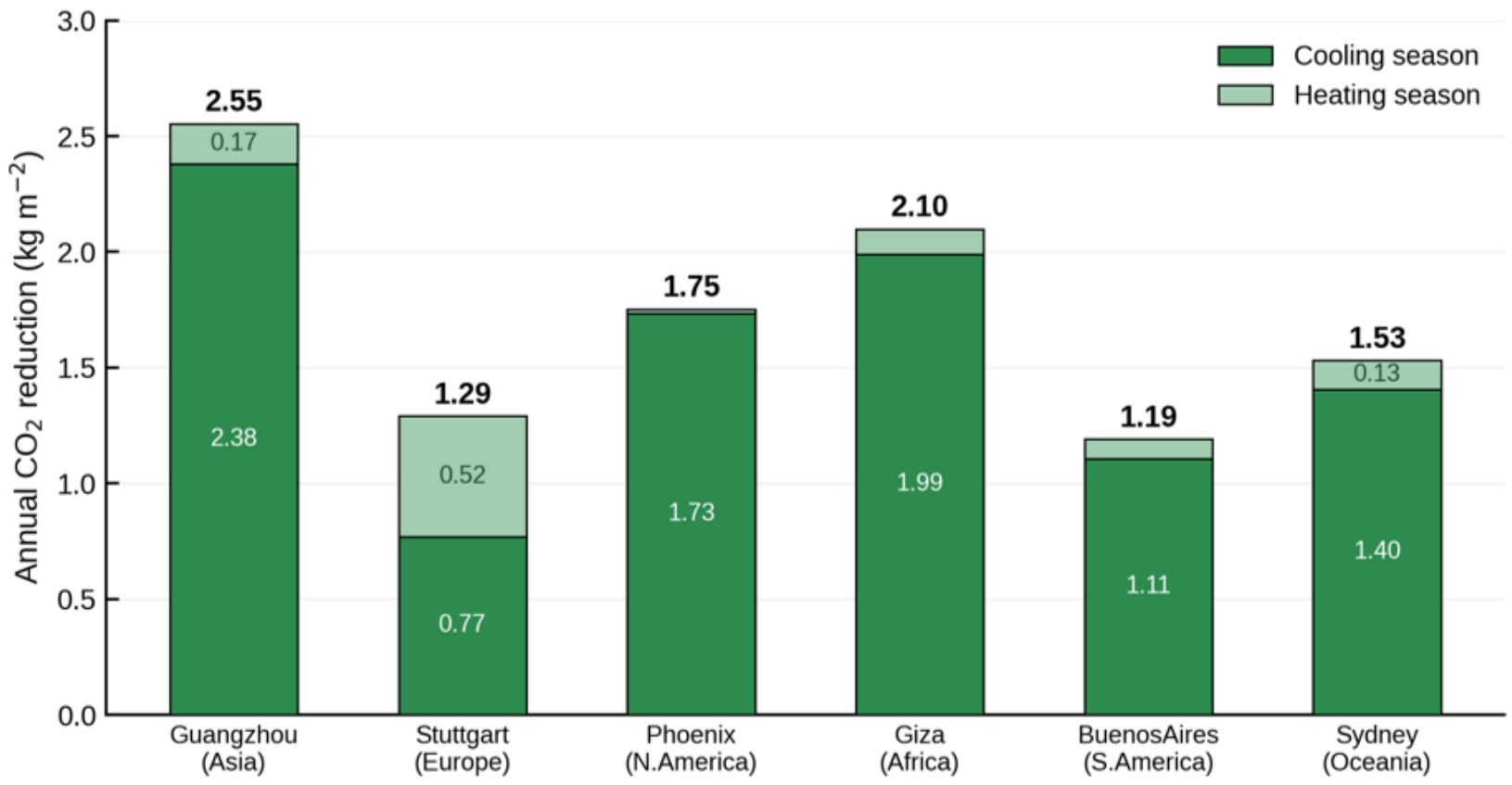

Figure S39. CO_2 mitigation enabled by engineered BSDAR bridging system on building. Global CO_2-emission reduction derived from the modeled energy savings combined with location-specific grid CO_2-emission factors, normalized by floor area.

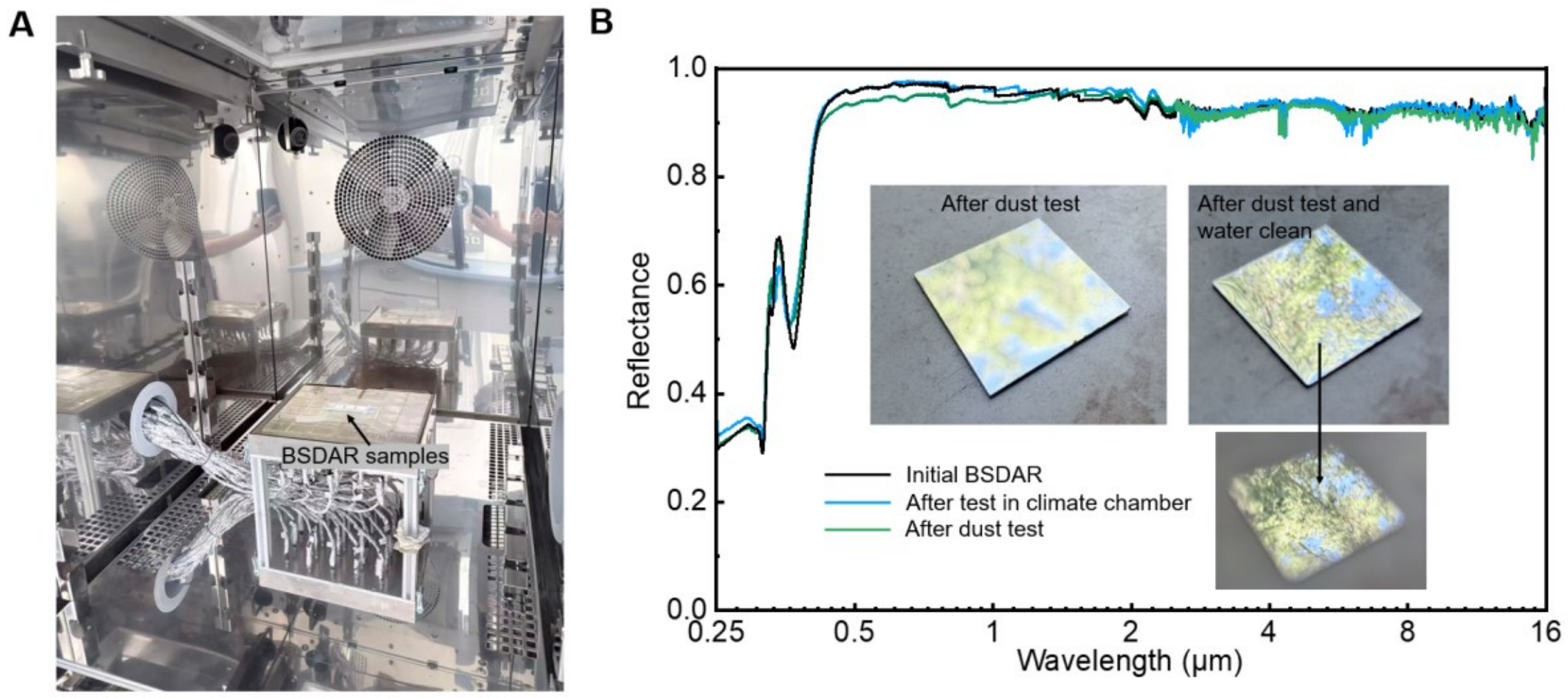

Figure S40. Outdoor stability and soiling behavior of BSDAR. (**A**) The climate chamber used for accelerated ageing, with BSDAR samples mounted inside. Samples were held at 85 °C and 85% relative humidity under one-sun irradiance for 100 h, following the damp-heat stress condition of IEC 61215-2. (**B**) Hemispherical reflectance spectra from 0.25 to 16 μm of the pristine sample (black), after the climate-chamber exposure (blue), and after ten days of unattended outdoor exposure (green; 14–23 July 2026). The climate-chamber exposure reduces the Planck-weighted (300 K) reflectance in the atmospheric window (8–13 μm), which governs the bridging function, by 0.22%, and the AM1.5G-weighted solar reflectance over 0.3–2.5 μm by 0.71%. Outdoor soiling reduces the same two quantities by 1.17% and 1.65% respectively; the attenuation increases toward shorter wavelengths, consistent with the scattering efficiency of micrometer-scale deposits declining as the wavelength approaches and exceeds the particle size, so that the function most critical to BSDAR is the least affected. Insets: photographs of the soiled sample and of the same sample after a single rinse with a water volume equivalent to one rainfall event per unit area followed by natural drying, showing complete removal of the deposit and restoration of the specular reflection of the surrounding scene.

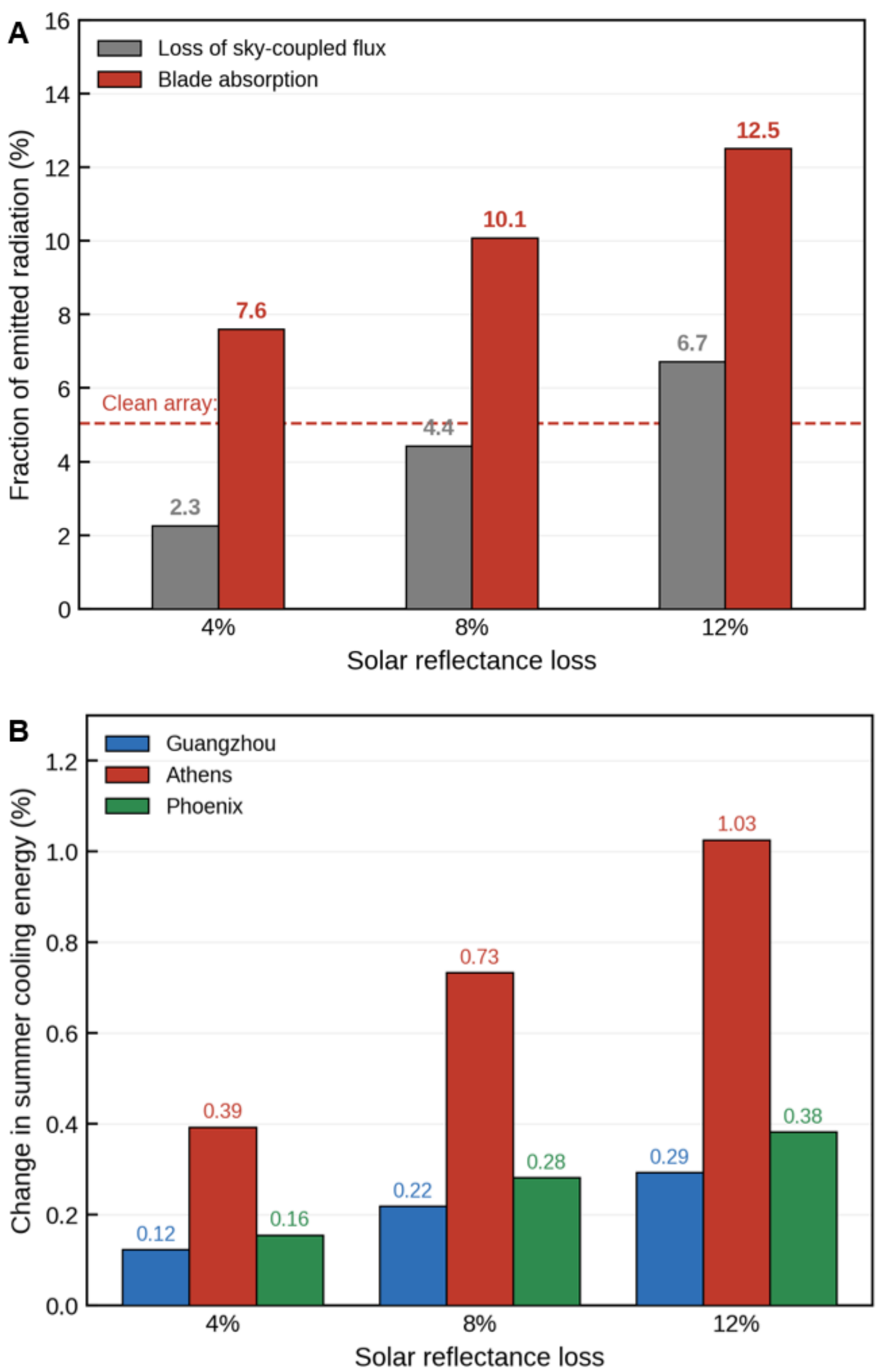

Figure S41. Effect of soiling on BSDAR performance. Soiling levels are defined by the loss in AM1.5G-weighted slat solar reflectance and are extrapolated from the measured ten-day exposure, in which a 1.65% solar reflectance loss was accompanied by a rise in atmospheric-window emissivity of 0.011; both quantities are scaled together in the same proportion. (**A**) Ray-tracing results for the infrared bridging function, showing the loss in the fraction of window-emitted radiation that reaches the sky, relative to the clean array, together with the fraction absorbed on the slats. (**B**) Magnitude of the change in summer cooling energy from EnergyPlus for three representative city's climates.

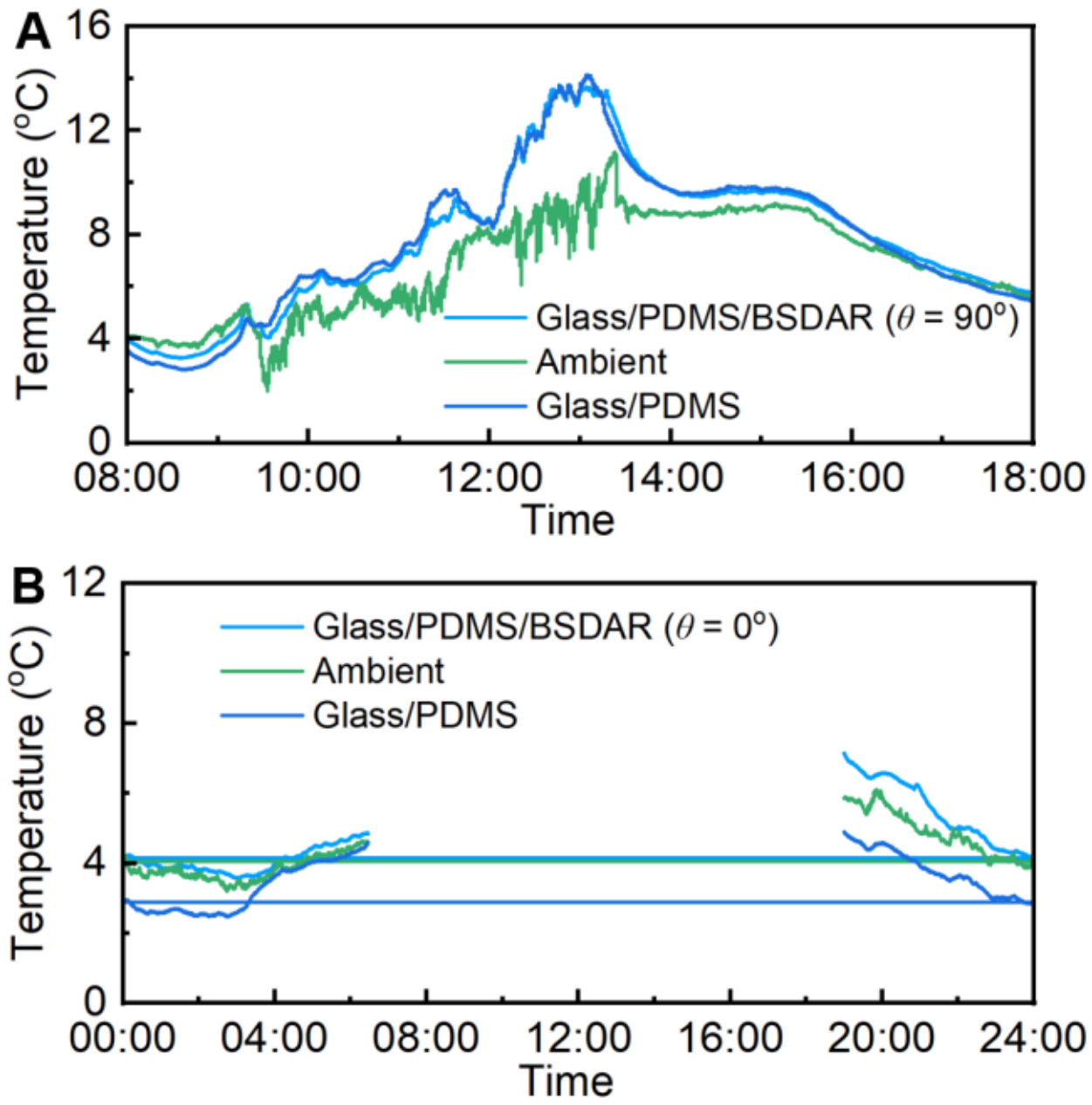

Figure S42. Season adaptivity of BSDAR system in winter. (**A**) Temperature–time profiles showing the absence of cooling when the BSDAR bridging system is oriented at $\theta = 90°$ during winter daytime operation, while maintaining natural daylighting. (**B**) Temperature–time profiles demonstrating the thermal insulation effect of the BSDAR bridging system at $\theta = 0°$ during winter nighttime operation.

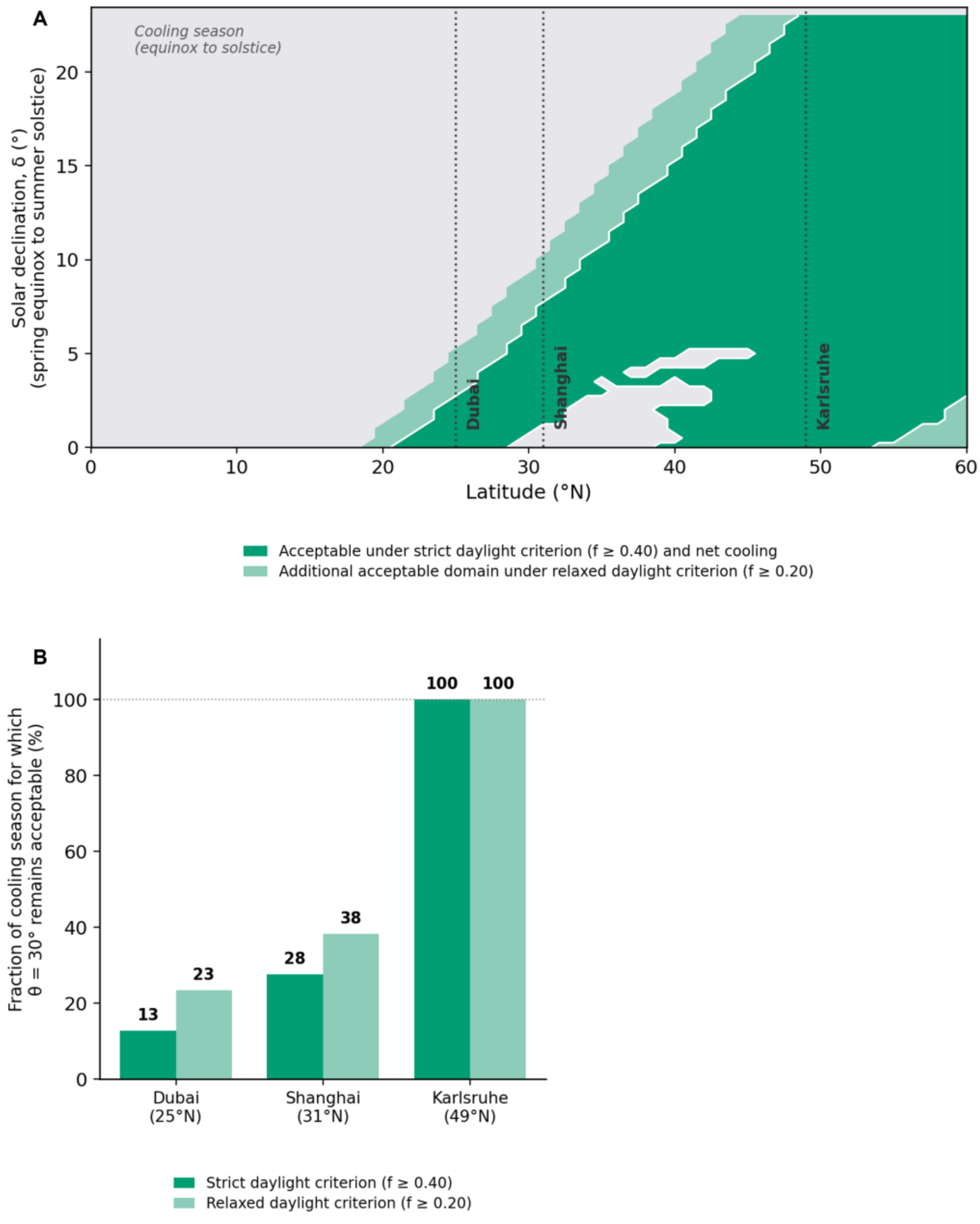

Figure S43. Acceptability of the fixed 30° blade across latitude and season. (**A**) Domain over which the fixed 30° configuration remains acceptable during the hot season required cooling. The horizontal axis is latitude and the vertical axis is solar declination, which advances from 0° at the spring equinox to 23.45° at the summer solstice and therefore represents the progression of the hot season required cooling; each point corresponds to a distinct daily solar trajectory. A configuration is acceptable where two conditions hold simultaneously: the daytime-averaged net heat load on the glazing is non-positive, and the admitted daylight fraction f meets the criterion, where $f = 1 - B$ and B is the fraction of direct sunlight intercepted by the slats, so that $f = 1$ corresponds to an unobstructed window and $f = 0$ to complete interception. Dark green marks the domain acceptable under the strict daylight criterion, $f \geq 0.40$; light green the additional domain admitted when the criterion is relaxed to $f \geq 0.20$; and

grey the remainder. Dotted lines mark the latitudes of Dubai (25°N), Shanghai (31°N) and Karlsruhe area (49°N). (**B**) Fraction of the hot season required cooling for which the fixed 30° configuration remains acceptable at each latitude, under the strict and relaxed criteria. Acceptability increases with latitude and is complete at 49°N under both criteria.

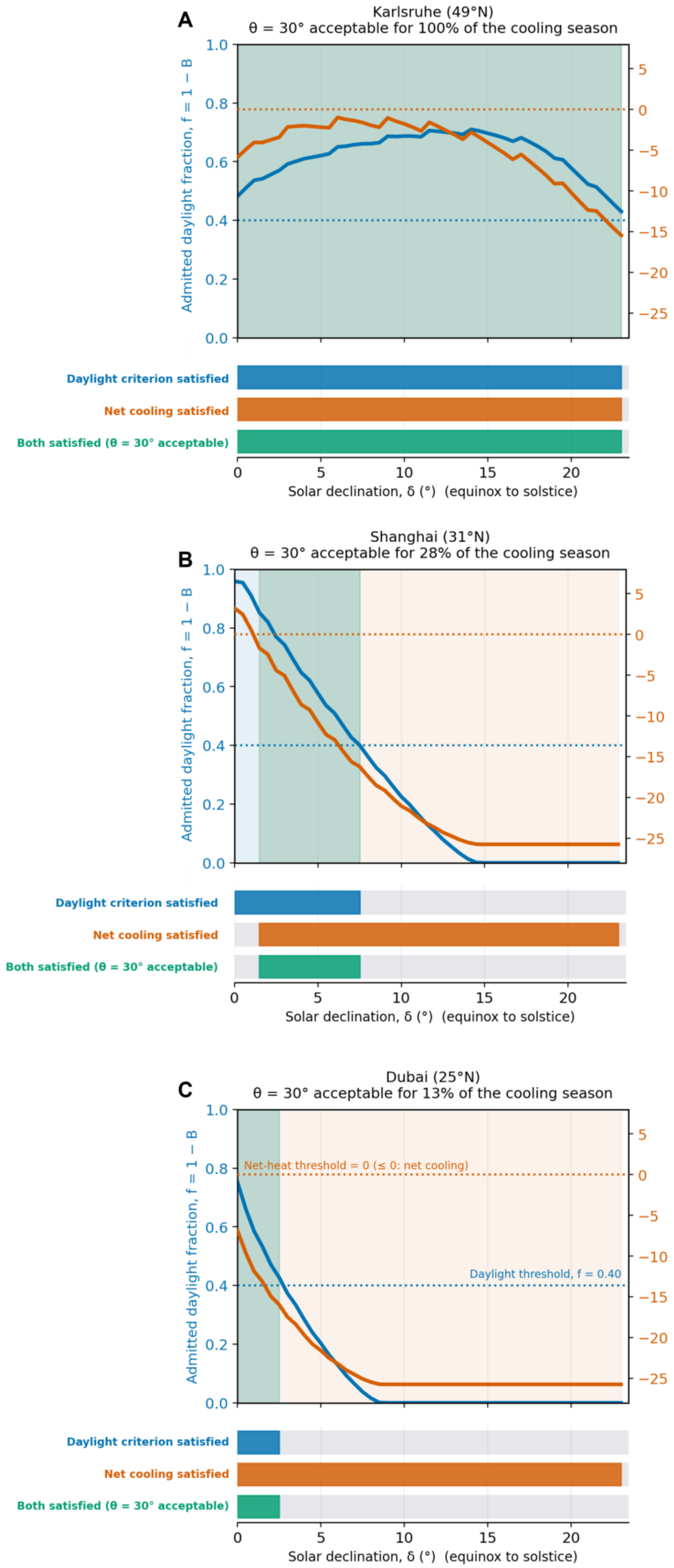
A
Karlsruhe (49°N)
θ = 30° acceptable for 100% of the cooling season
Admitted daylight fraction, f = 1 − B
Daylight criterion satisfied
Net cooling satisfied
Both satisfied (θ = 30° acceptable)
Solar declination, δ (°) (equinox to solstice)
B
Shanghai (31°N)
θ = 30° acceptable for 28% of the cooling season
Admitted daylight fraction, f = 1 − B
Daylight criterion satisfied
Net cooling satisfied
Both satisfied (θ = 30° acceptable)
Solar declination, δ (°) (equinox to solstice)
C
Dubai (25°N)
θ = 30° acceptable for 13% of the cooling season
Net-heat threshold = 0 (≤ 0: net cooling)
Daylight threshold, f = 0.40
Admitted daylight fraction, f = 1 − B
Daylight criterion satisfied
Net cooling satisfied
Both satisfied (θ = 30° acceptable)
Solar declination, δ (°) (equinox to solstice)

Figure S44. Which criterion limits the fixed 30° blade at each latitude. Upper panels: admitted daylight fraction and net window heat load as functions of solar declination, for three latitudes spanning 25°N to 49°N. The admitted daylight fraction is $f = 1-B$, where B is the fraction of direct sunlight intercepted by the slats, so that $f = 1$ corresponds to an unobstructed window and $f = 0$ to complete interception; it is plotted in blue against the left axis. The net window heat load, plotted in orange against the right axis, is the daytime-averaged balance of absorbed solar gain, long-wave gain from the ground and radiative loss redirected to the sky; negative values indicate net cooling.

Dotted lines mark the strict daylight threshold, $f = 0.40$, and the net-cooling threshold of zero.

Lower bars: the declination range over which each criterion is satisfied individually, and the range over which both are satisfied simultaneously. At 25°N and 31°N the net-cooling criterion is met over most or all of the hot season required cooling while the daylight criterion is met only near the equinox, so daylight is the limiting factor; at 49°N (Karlsruhe area) both criteria are met throughout.

Table S1. Comparison of cooling performance among this work and advanced vertical surface cooling reports, thermal testing conditions reported for radiative cooling of vertical building surfaces.

Study	Wind shield	Thermal insulation	Sample size and mounting	Terrestrial heat source	Reported temperature depression	Tested on an actual building	Classification
This work (calorimetric configuration)	Yes	Yes	Bench-scale BSDAR module on an insulated test rig	Real ground under clear sky	Up to 4.1 °C for sub-ambient reduce	Yes, rig mounted on a building window	Calorimetric
This work (field configuration)	None	None	Enlarged BSDAR array retrofitted onto an in-service south-facing window; unconditioned room, purely passive	Real ground and urban surroundings; ambient above 30 °C	0.907 °C for sub-ambient reduce, daytime mean (hot-weather campaign)	Yes, BSDAR directly mounted on a building window	Ambient

Study	Wind shield	Thermal insulation	Sample size and mounting	Terrestrial heat source	Reported temperature depression	Tested on an actual building	Classification
Xie et al., Science 386, 788 (2024)[1]	Yes, PE wind shield within a clear-acrylic enclosure; a nanoPE-only variant was also tested	Yes, copper block on a thermal insulator	10 × 10 cm (vacuum chamber) and 35 × 35 cm (outdoor); free-standing test rig	Outdoor ground at 44–53 °C	2.5 °C for sub-ambient reduce	No	Calorimetric
Cheng et al., Nexus 1, 100028 (2024)[2]	Yes, PE-film-covered variant reported separately	Yes, polystyrene foam enclosure with an aluminium frame	15 × 15 cm samples on a foil-clad cardboard box (102 × 41 × 36 cm)	Real ground at 23–56 °C (field)	approx. 1.2 °C for sub-ambient reduce	No	Predominantly calorimetric
Degeorges et al., Joule 9, 101956 (2025)[3]	No	Yes, radiatively shielded R-26 foam	Small coupons mounted vertically on foam board	Real ground for the summertime configuration	Not achieved for sub-ambient reduce	No	Predominantly calorimetric

Study	Wind shield	Thermal insulation	Sample size and mounting	Terrestrial heat source	Reported temperature depression	Tested on an actual building	Classification
Wu et al., Science 384, 1203 (2024)[4]	No; foil and silver mylar shielded the rig from sunlight only	Yes, polystyrene foam with a copper backing plate	Small aperture in a foam block; a 0.68 m wallpaper wall at 1.86 m emulated an urban canyon	Real ground above 60 °C	Not achieved for sub-ambient reduce	No	Predominantly calorimetric
Mandal et al., arXiv:2006.11931 (2020)[5]	No, stated explicitly as tested without convection or radiation shields	Yes, styrofoam backing	Small coupons in a car park (Los Angeles) and on a meadow slope (Nashville)	Real ground; urban	Not achieved or no reported for sub-ambient reduce	No	Ambient
Chen et al., Nano Lett. (2026)[6]	Yes, PE convection shield used	Yes	TADE-integrated glazing in an outdoor test cell	Real ground (field)	Not achieved or no reported for sub-ambient reduce	No	Calorimetric

Note: Temperature reductions are reported relative to the ambient-temperature reference, with positive values indicating that the surface temperature is below ambient temperature. Cases where the surface temperature does not fall below ambient temperature are marked as "Not achieved". For a conservative, like-for-like comparison, we focus on summer daytime, south-facing conditions, which represent the most challenging scenario for building facades. Studies that rely on hybrid radiative–evaporative cooling are excluded from this comparison.

Table S2. Comparison of BSDAR with representative commercial external venetian blind and low-emissivity glazing window.

	**External venetian blind**	**Low-emissivity glazing**	**BSDAR**
Representative product	Sonnenschutz	Saint-Gobain SGG PLANITHERM ONE	This work
Construction	Roll-formed powder-coated aluminum slats	Magnetron-sputtered low-emissivity coating on float glass	PVD Ag with TiO_2 overcoat on anodized aluminum
Slat width	80 mm	—	80 mm
Coating position	Slat surface, external to the window	Face 2 or 3 of the insulating unit	Slat surface, external to the window
Solar reflectance	~0.7	Light transmittance 72%, g = 0.52	~0.9 (AM1.5G)
Infrared emissivity	~0.9	~0.03	~0.08
Primary heat flow controlled	Solar gain	Conduction and radiation through the glazing, Ug = 1.0 W $m^{-2}K^{-1}$	Solar gain and net radiative to the sky
Sub-ambient cooling	No	No	Yes
Daylight Glare Probability at solar noon	0.401†	—	0.377†
Retrofit	Attached to the existing window	Replacement of the insulating glass unit	Attached to the existing window
Seasonal adjustability	Slat angle and deployment adjustable	No, a fixed coating	Slat angle and deployment adjustable

	External venetian blind	Low-emissivity glazing	BSDAR
Indicative cost	380–490 US$ manual, 600–820 US$ motorized per 1.2 × 1.4 m window, plus 80–150 € installation‡	65–105 US$ m^{-2} glazing plus 27–49 US$ m^{-2} installation; 545–1440 US$ per window if the frame is replaced‡	Same system cost as an external venetian blind, plus a reflector premium of about 12 US$ m^{-2} of slat area over powder-coated aluminium§

† From the controlled comparison at identical slat geometry reported in this work.

‡ German market survey, 2025–2026, converted at 1 € = 1.09 US$.

§ Estimated from 15–19 US$ m^{-2} at 0.3–0.4 mm thickness, against a few US$ m^{-2} for powder-coated aluminium strip.

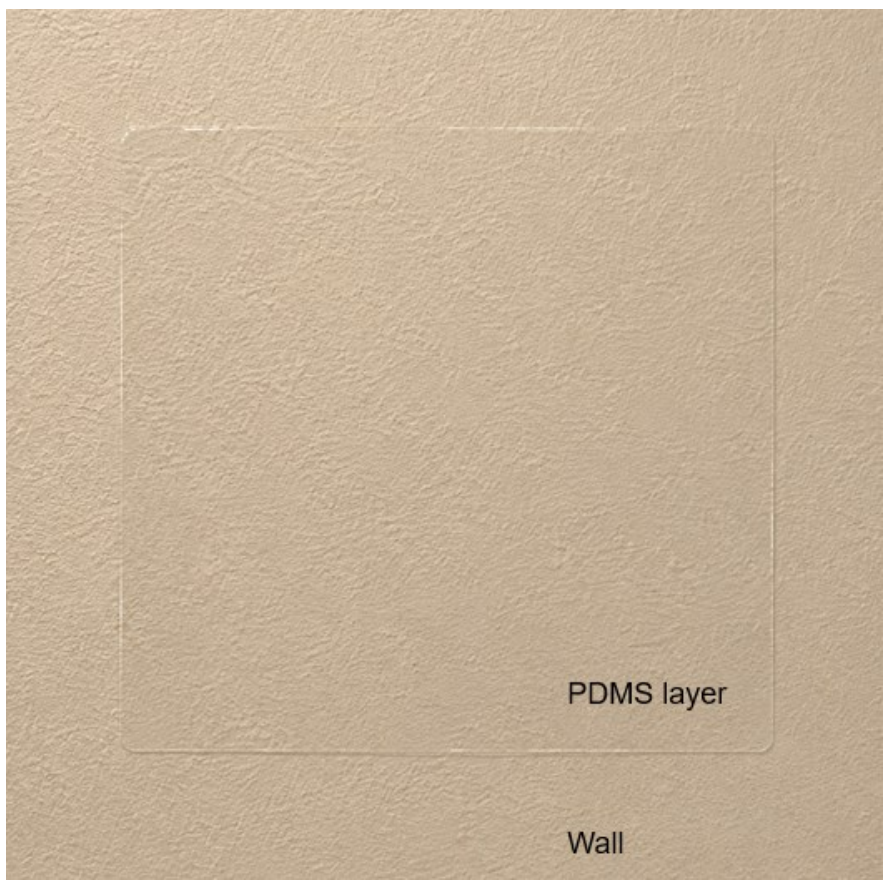

Figure S45. PDMS applied to a rough wall surface after ten days' exposure. PDMS prepolymer was mixed with its curing agent at a ratio of 10:1 by weight and left to stand for 10 min before application, so that the initial viscosity was high enough to limit downward flow on a vertical surface. The mixture was then cast directly onto a rendered masonry wall, allowing the liquid precursor to fill the surface porosity before crosslinking. Entrapped air was worked out toward the lower edge during application, and additional material was applied at the upper edge to compensate for gravitational flow and obtain a comparable thickness over the height of the sample. The film was cured in situ at ambient temperature for 8 h and then trimmed at the perimeter. For larger areas the flow of the liquid precursor on a vertical surface would be controlled by a thixotropic additive or by spray application in future.

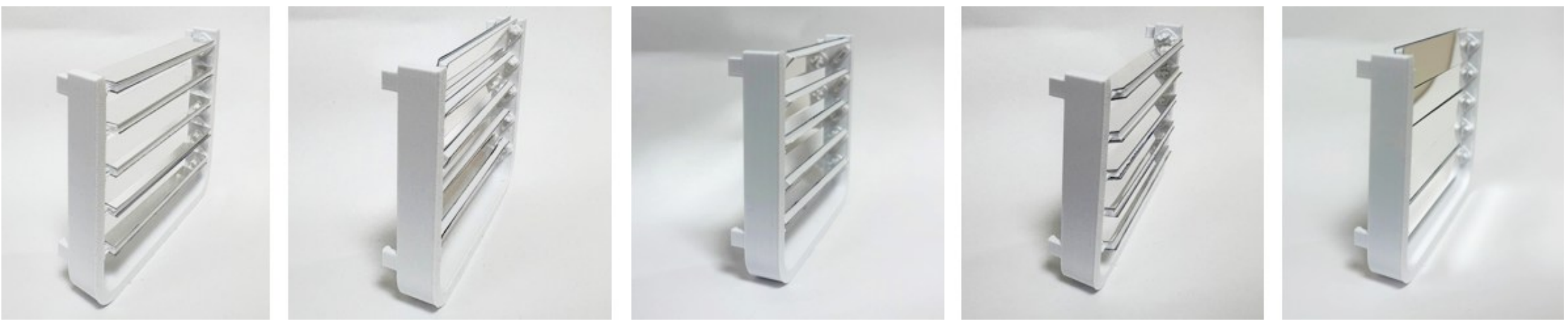

Figure S46. Optical images showing the double-sided BSDAR fixed in angle and spacing using a 3D-printed frame, which used in testing in Figure 2. From left to right, the array is presented at angles of 90°, 30°, 45°, 60°, and 0° relative to the vertical plane. These are the outer frames of the test setup. The clips on the left side of these frames are used for quick connection to the inner frame.

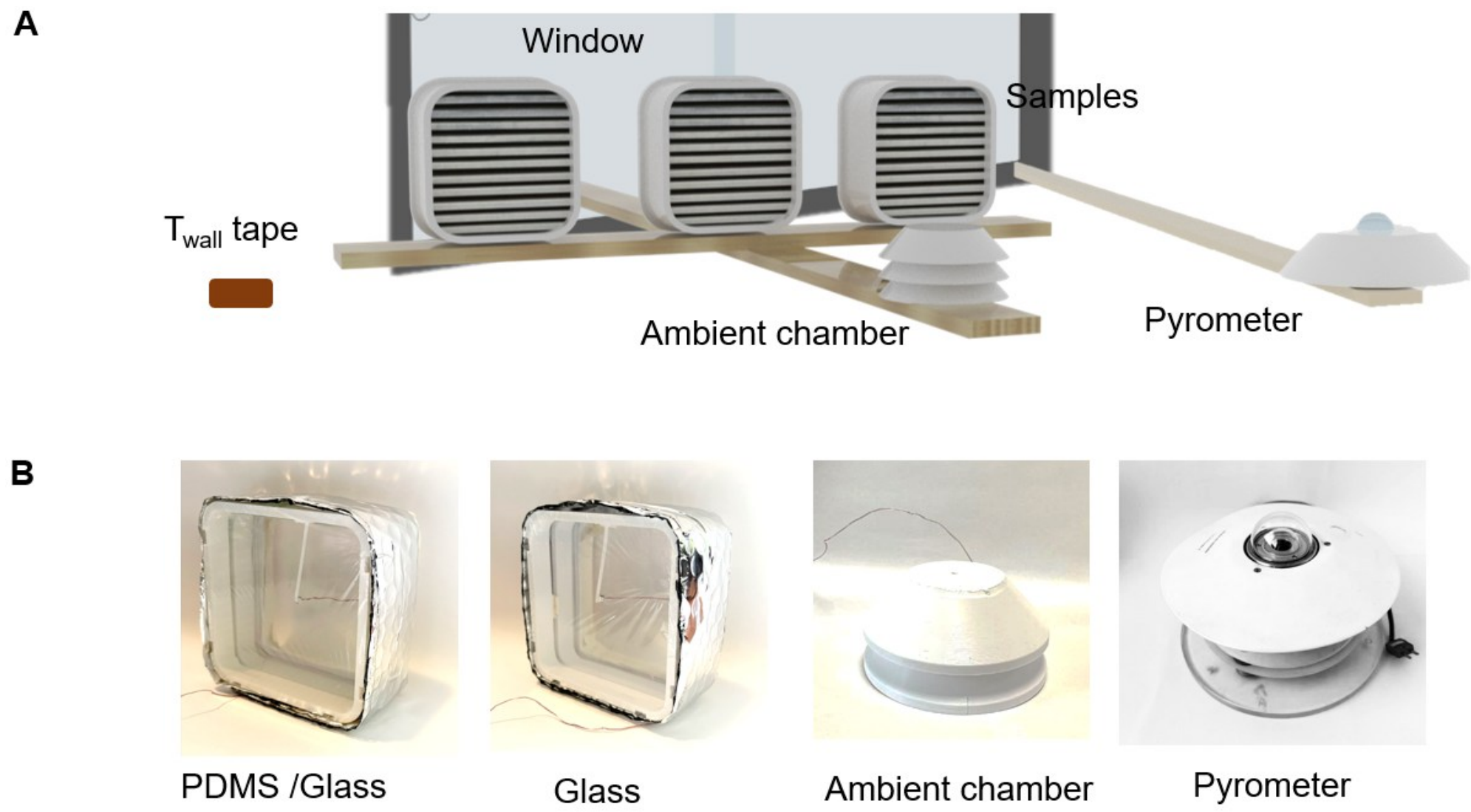

Figure S47. Details of the small-scale test setup used in Figure 2. (**A**) Schematic of the experimental configuration for small-scale window testing. (**B**) Photograph of the test samples and measurement fixtures.

Panel (**B**) shows the inner frame, which can be rapidly attached to the outer frame via the front-edge grooves. A radiation-shielded weather-station housing (which provides airflow to prevent heat buildup and associated overestimation of ambient temperature) was placed at the same height as the samples but laterally offset (not directly adjacent), to avoid radiative contamination from the insulating and reflective materials on the exterior of the sample devices. The internal component layout within the inner frame is described in the Materials and Methods.

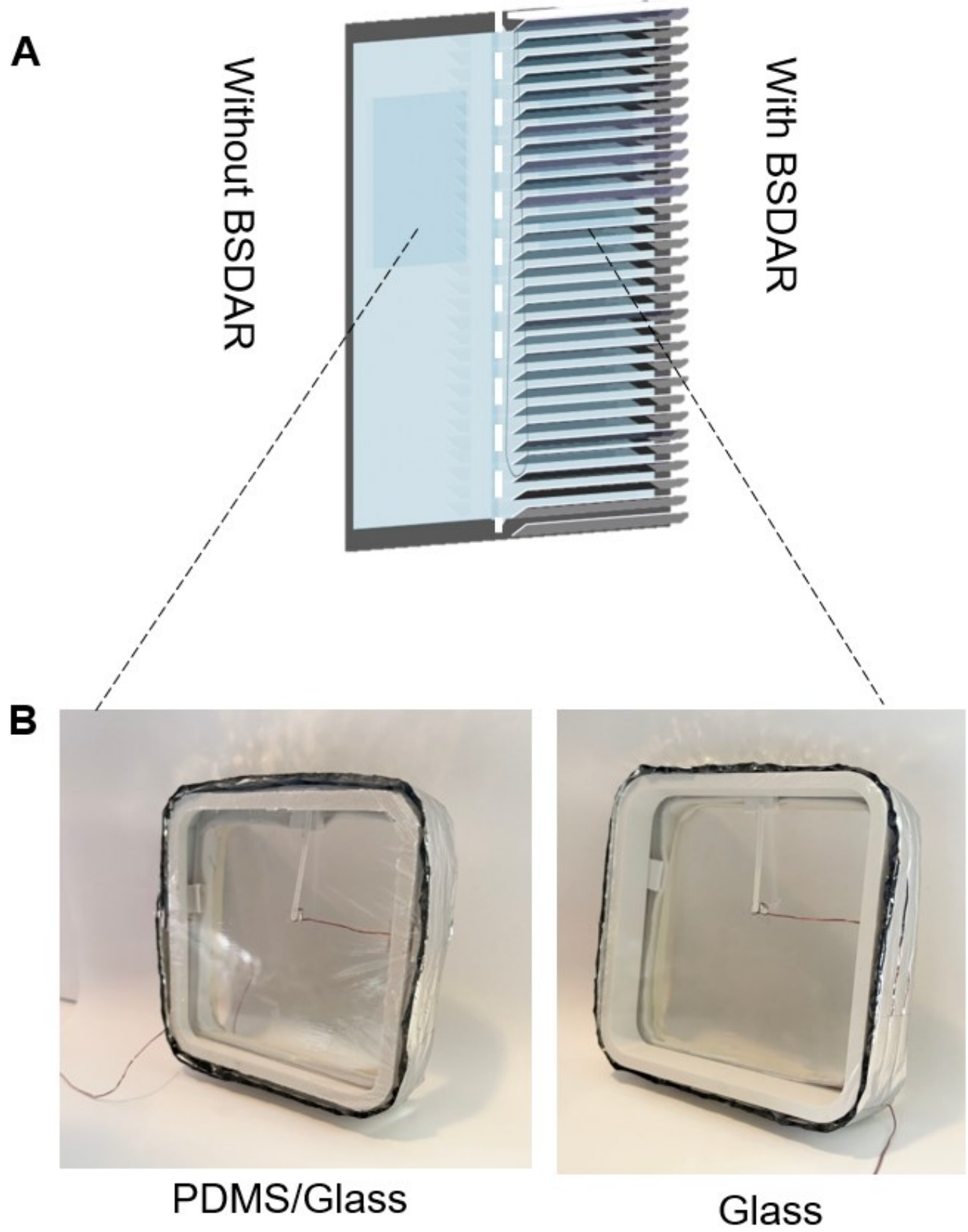

Figure S48. Details of the testing setup for the engineered, scaled-up BSDAR installed on a building window in Figure 3. (**A**) Schematic illustrating the measurement configuration. (**B**) Photograph of the sensor frame positioned behind the scaled BSDAR.

To minimize the influence of wind and convective heat transfer on the local temperature measurements, we fabricated a sensor frame that follows the same materials and shielding concept as the small-scale device in Figure 2, but with a substantially reduced frame width to limit flow disturbance. The sensor frame was directly attached to the window and placed behind the engineered BSDAR to quantify the cooling performance of the scaled-up system. This configuration enables paired temperature measurements at the same height on the same window, with and without the BSDAR, ensuring identical boundary conditions and minimizing mutual interference between the two measurement states.

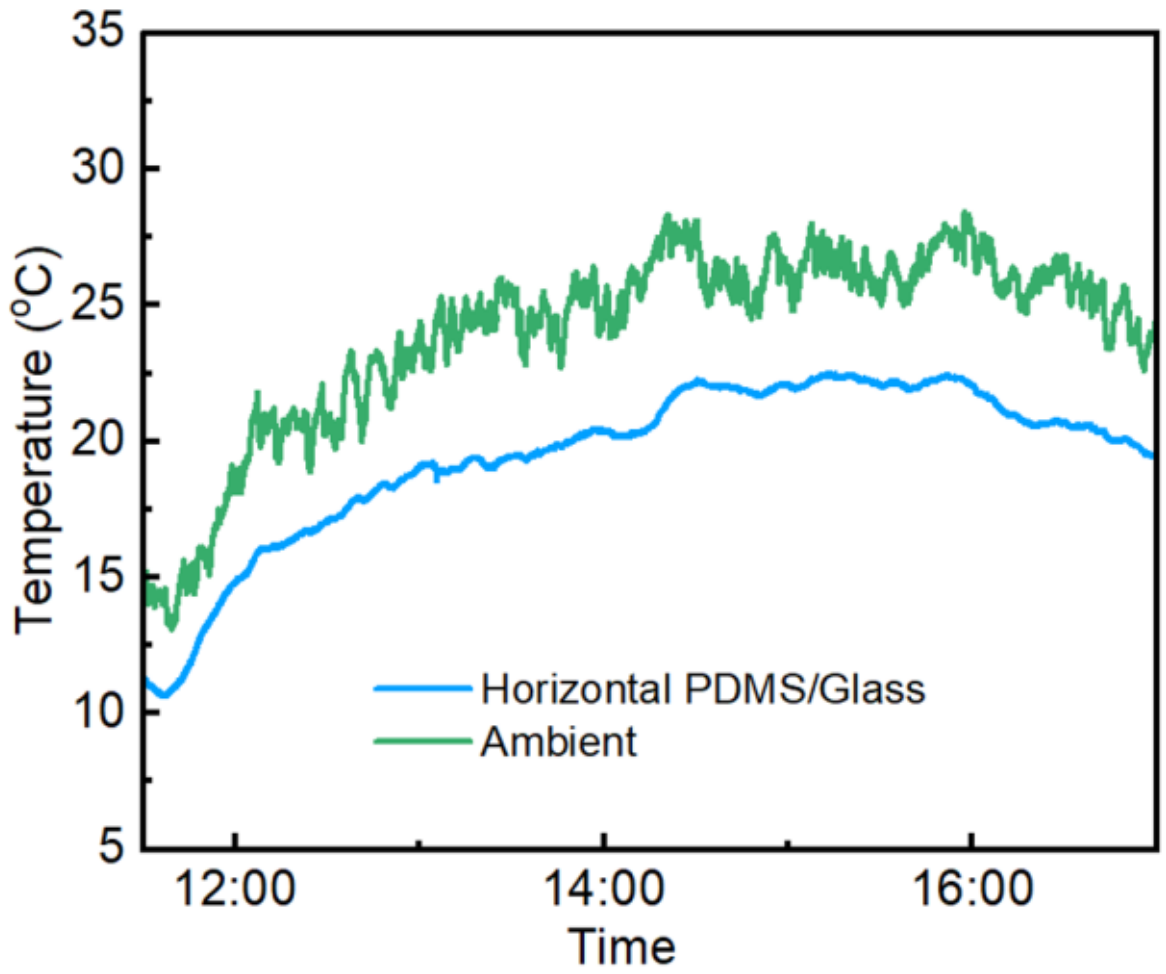

Figure S49. Temperature vs. time curve of a horizontally oriented PDMS/glass sample during daytime. To establish a baseline for the intrinsic cooling performance of the PDMS horizontal radiative cooling layer, we used the small device from Figure 2 without the BSDAR outer frame and placed the PDMS/glass sample horizontally, facing upward toward the sky, on a table (63 cm above the ground). The test was performed at the street-side location adjacent to the south-facing test window in Eggenstein-Leopoldshafen, Germany, on September 6, 2025. Under this real urban street-canyon environment, the upward-facing PDMS/glass sample achieved an average cooling of 6.0 °C below ambient, with a maximum cooling of 6.9 °C below ambient (over the measurement period). This baseline performance is representative of commonly reported horizontal radiative cooling materials, supporting its use as a reference horizontal radiative cooling layer. In addition, the PDMS/glass sample offers high optical transparency and mechanical conformability, making it suitable for deployment on vertical window facades and for evaluating whether the designed BSDAR bridging system can enable the common horizontal radiative cooling material achieve the below-ambient cooling on vertical surfaces.

Supplementary Text

S3. Directional infrared transport through the BSDAR array

The bridging function of BSDAR rests on the slats redirecting the glazing's thermal emission toward the sky rather than absorbing it. Because a surface-to-surface radiation solution does not resolve individual ray paths, this section quantifies the transport by direct tracing and verifies it experimentally.

Experimental verification of directionality. Imaging the same array simultaneously from two viewing directions gives opposite results (Figure S50). Viewed from the front, the slats appear hot, because the camera looks along the direction from which they reflect infrared radiation arriving from the opposite building and the ground. Viewed from above, the same slats appear cold, because the camera then looks along the direction into which they redirect the glazing's own emission and sees the low apparent temperature of the sky. A surface emitting or absorbing diffusely would appear close to its physical temperature from every direction, so a difference of this magnitude between two simultaneous views is possible only for a specular reflector.

The slats do not accumulate the emission. Measuring the rear surface of the slats alongside a commercial aluminum blind of identical geometry separates the two cases (Figure S51). The rear of the commercial blind is the warmest surface recorded and stays above ambient, and the glazing behind it is correspondingly warmer; the rear of BSDAR remains at ambient. Absorbing slats in commercial blinds therefore accumulate heat and pass it to the window, whereas the low-emissivity specular BSDAR slats do not.

Transport statistics. Tracing every emitted ray to termination gives four fractions that sum to unity: 43.5% reaches the sky, 6.2% reaches the ground, 45.4% returns to the glazing, and 5.0% is absorbed on the slats (Figure S52A). The mean number of specular reflections is 1.01, with 22.3% of rays undergoing none, 56.7% one, 19.9% two and 1.1% three or more; the largest value among 120000 rays is 26 (Figure S52B). Rays that return to the glazing and rays that escape carry indistinguishable reflection counts, 1.01 against 0.98, so the outcome of a ray is set by its emission direction rather than by an accumulation of reflections. Repeating the tracing over a range of geometries shows that the reflection count is governed by the ratio of vertical slat projection to pitch, and not by the slat-to-glazing gap, which leaves the mean between 0.94 and 1.01 when varied from 2 to 120 mm.

Net radiative consequence. Converting the four fractions to radiative flux separates one-way transport from net exchange (Figure S53). The channel returning to the glazing carries the largest one-way flux of the four and yet contributes exactly zero net flux, because the surface at the far end of that channel is the glazing itself and the exchange involves no temperature

difference; this holds for any temperature. Slat absorption of 5.0% is therefore the only radiative loss within the array, and the directional redirection delivers 43.7% of the glazing's emission to the sky against 6.0% to the ground.

Supplementary Text

S4. Winter heat loss with the array closed

The seasonal strategy closes the array at night during the cold season required heating. This section quantifies the resulting change in heat loss through the window and identifies which transport mechanism is responsible.

Closing the array places an 8 mm air layer in front of the glazing. Whether that layer circulates is governed by its Rayleigh number,

$Ra = g\beta \Delta T\, L^3 / (\nu\alpha)$,

with L the gap width, ΔT the temperature difference across it, β the thermal expansion coefficient of air taken as $1/\bar{T}$, and ν and α its kinematic viscosity and thermal diffusivity. For an 8 mm gap at a typical winter night condition of 20 °C indoors and 0 °C outdoors, Ra is 829 with BSDAR and 574 with a commercial blind, both below the threshold of about 1000 at which convection sets in; onset would require an indoor-to-outdoor difference of 24 K and 35 K respectively. Heat therefore crosses the layer by conduction and radiation rather than by an air circulation, so the insulating effect does not depend on the array blocking wind.

The heat loss was evaluated on this basis as a one-dimensional series of thermal resistances: the indoor surface resistance, the conduction resistance of the glazing, the resistance of the air layer, and the outdoor surface resistance. The glazing is the single 3 mm pane used in the energy model, of thermal conductivity 0.9 W m^{-1} K^{-1} and infrared emissivity 0.99. Across the air layer the conductive contribution is the thermal conductivity of air divided by the gap width, and the radiative contribution is the linearized exchange between two grey surfaces, $4\sigma\bar{T}^3/(1/\varepsilon_{glass} + 1/\varepsilon_{slat} - 1)$, with $\varepsilon = 1 - \rho$ for an opaque surface. The slats stand 8 mm clear of the glazing and are not in contact with it, so they do not form a thermal bridge, and their own resistance is negligible in any case, being 0.2 mm of aluminum. Values correspond to an indoor temperature of 20 °C and an outdoor temperature of 0 °C.

This gives a window U-value of 6.36 W m^{-2} K^{-1} for the bare window, 3.38 W m^{-2} K^{-1} with a commercial blind closed and 2.16 W m^{-2} K^{-1} with BSDAR closed, reductions in heat loss of 47% and 66% respectively. The difference between the two devices arises entirely from the radiative term: the atmospheric-window reflectance of a commercial slat is about 0.1 against 0.92 for BSDAR, so the specular surface suppresses that term by more than an order of magnitude. White and dark commercial slats give the same value, since they share the same infrared properties and color plays no part at night, which confirms that the winter benefit is set by the infrared reflectance rather than by the solar reflectance of the slat.

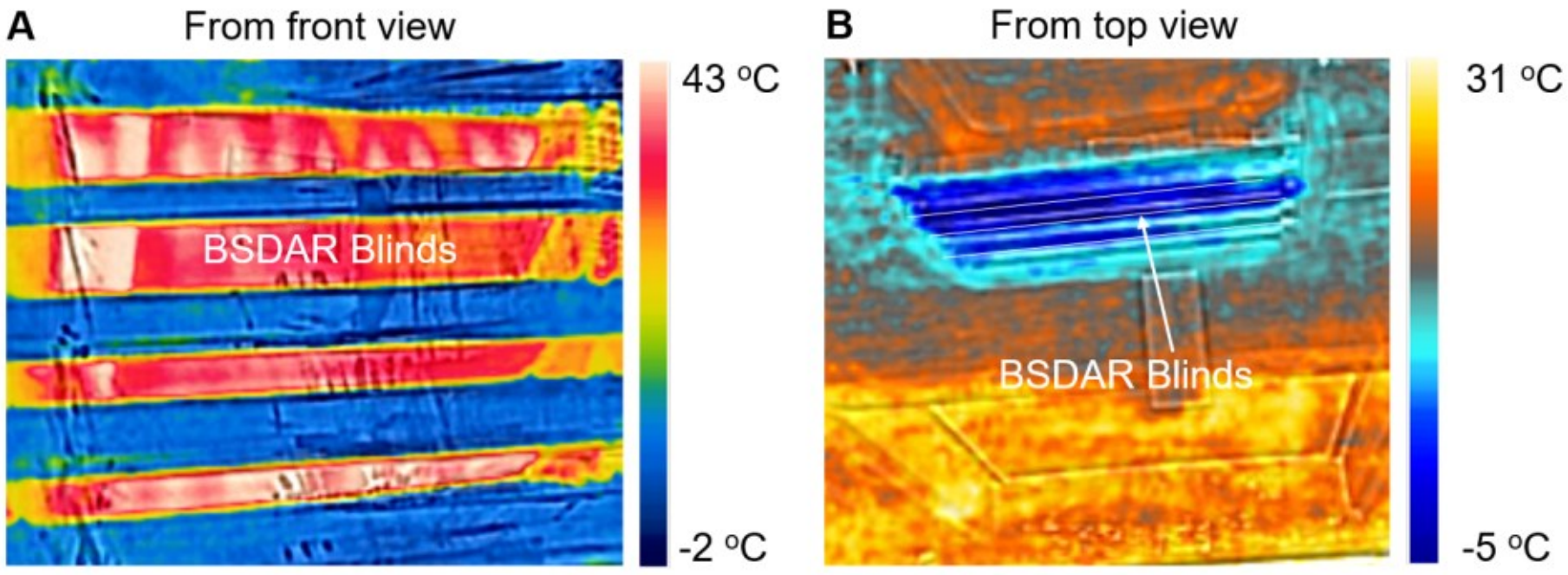

Figure S50. Directional thermography of the BSDAR array. Infrared images of the same array recorded simultaneously from two viewing directions under sunny condition. (**A**) Viewed from the front, the slats appear hot: the camera looks along the direction from which the slats reflect infrared radiation arriving from the opposite building and the ground, so the apparent temperature is that of those surroundings rather than of the slats. (**B**) Viewed from above, the same slats appear cold: the camera now looks along the direction into which the slats redirect the window's own emission, and therefore sees the low apparent temperature of the sky. Both images were taken at the same instant, so the difference cannot arise from a change in ambient conditions. A surface that emitted or absorbed diffusely would appear close to its own physical temperature from every direction; a difference of this magnitude between two viewing directions is possible only for a specular reflector. The values in both panels are apparent radiative temperatures along the line of sight and not the physical temperature of the slats, which was measured separately and remains close to ambient (Figure S51). The oblique top-down view of Figure S15 lies between these two directions, which is why the slats there show an intermediate apparent temperature.

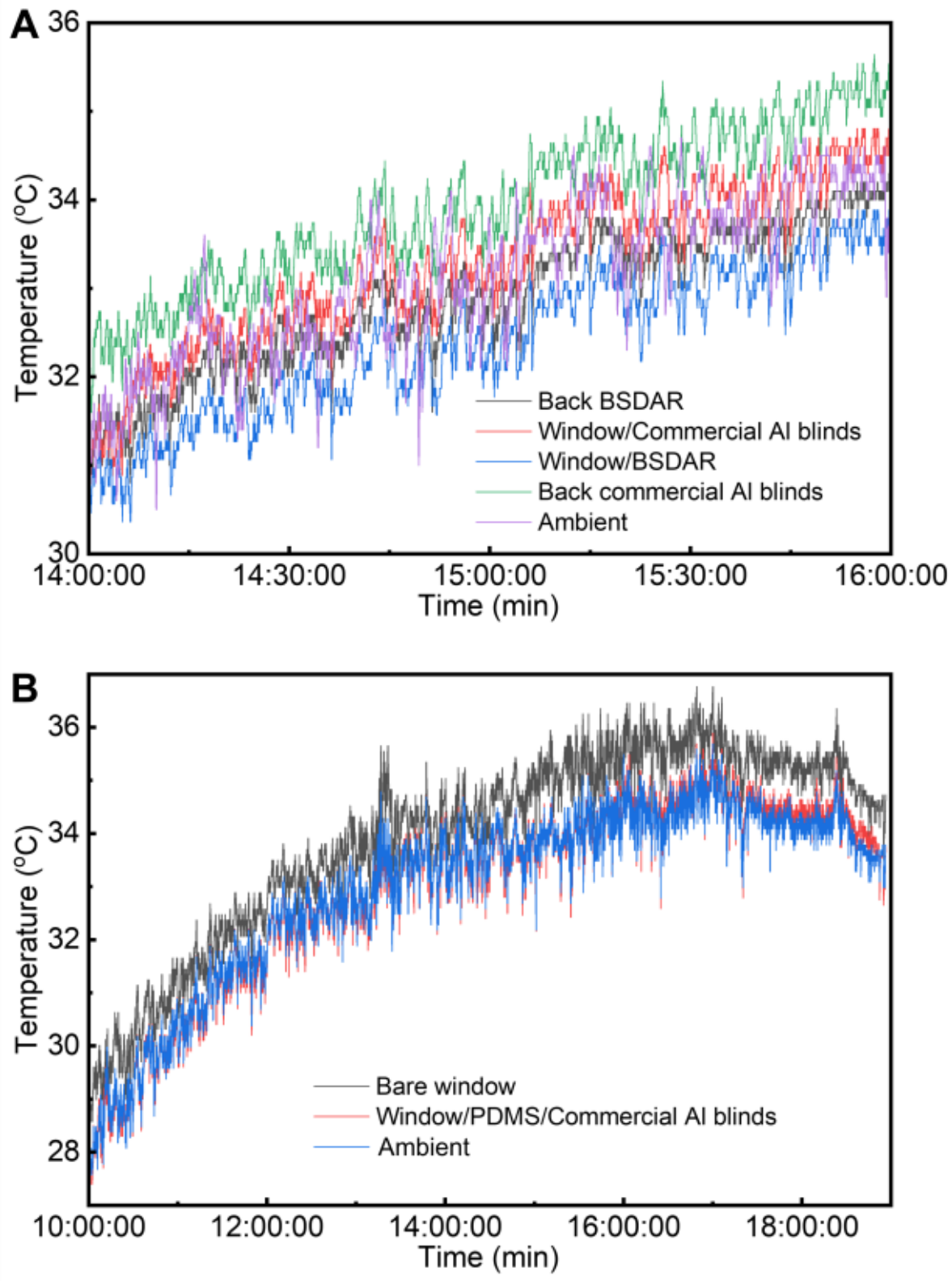

Figure S51. Slat and window temperatures for BSDAR and a commercial aluminum blind. (**A**) Temperatures measured side by side on a real building south-facing window on 12 July, 2026: the rear surface of the slats and the glazing surface behind them, for BSDAR and for a commercial aluminum blind of identical slat geometry, together with ambient air temperature. The rear of the commercial blind is the warmest surface recorded and stays above ambient throughout, whereas the rear of BSDAR remains at or below ambient; correspondingly, the glazing behind the commercial blind is warmer than the glazing behind BSDAR. Absorbing slats therefore accumulate heat and pass it to the window, while the low-emissivity specular slats of BSDAR do not. The glazing carries no PDMS coating in this measurement, so that the heat transferred from the slats to the window is isolated from the radiative cooling of the emitter itself; the comparison therefore reflects only the difference between the two slat surfaces. (**B**) Glazing surface temperature on further south-facing panes of the same building on the same day, for a bare window and for a window fitted with PDMS and a commercial aluminum blind, together with ambient air temperature, shown for reference. All panes are of the same construction and orientation, so the configurations are directly comparable.

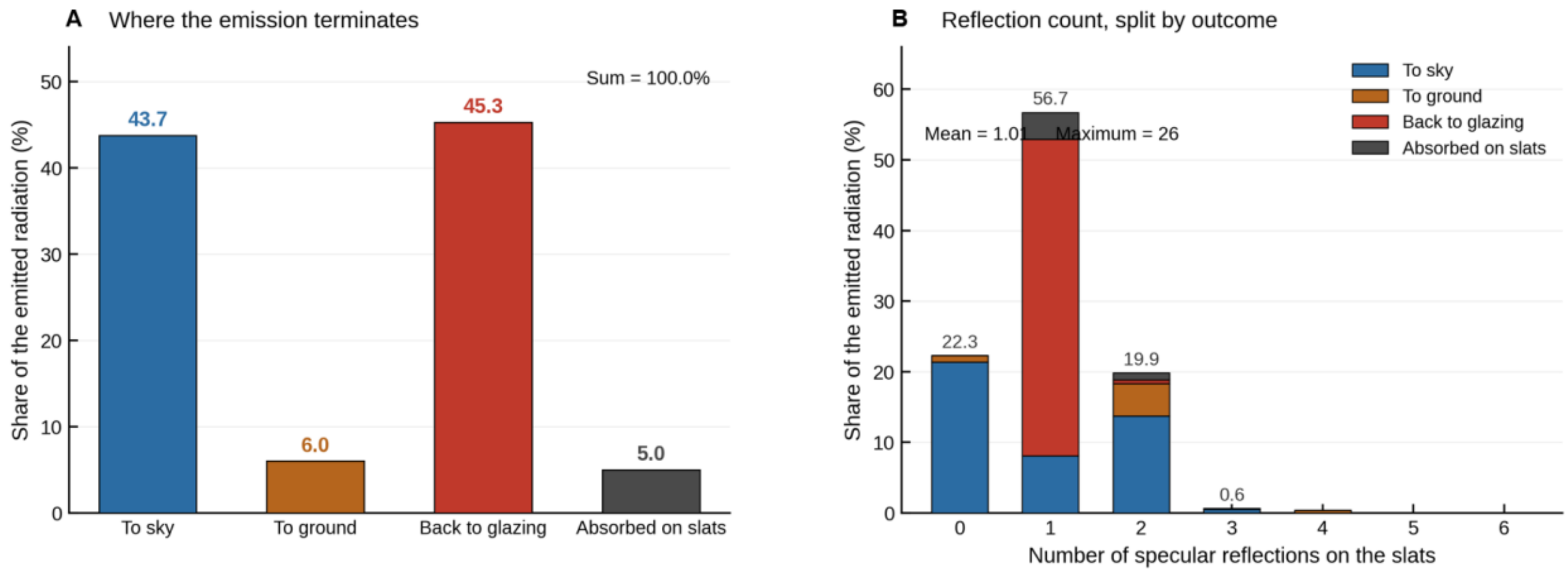

Figure S52. Statistical quantification of the infrared transport through the array. Each of 120000 rays emitted by the PDMS-coated glazing, with cosine-weighted directions, was traced through the as-built geometry until it terminated. A ray terminates in one of four ways: it escapes upward and reaches the sky, it escapes downward and reaches the ground, it returns to the glazing and is reabsorbed there, or it is absorbed on a slat. (**A**) The four terminal fractions, which sum to unity and therefore account for the whole of the emitted radiation. (**B**) The same rays grouped by the number of specular reflections undergone on the slats before terminating, each bar divided by outcome using the colors of panel (A). The mean is 1.01 reflections and the largest value observed among 120000 rays is 26. Rays that return to the glazing and rays that escape carry statistically indistinguishable reflection counts, 1.01 against 0.98, so the outcome of a ray is set by the direction in which it was emitted rather than by an accumulation of reflections inside the array.

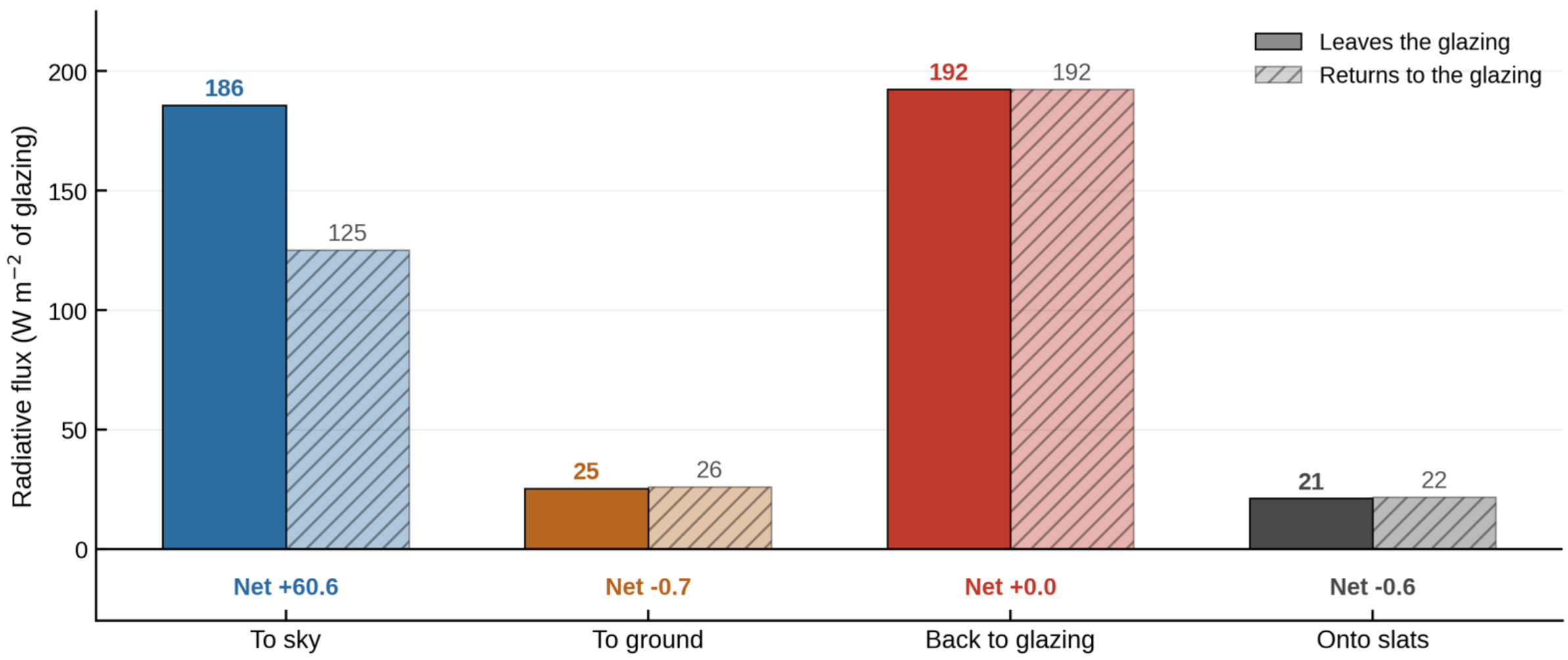

Figure S53. One-way and net radiative flux in each transport channel. The four transport fractions of Figure S52 converted into radiative flux per unit glazing area for a worked example at a glazing temperature of 25 °C, an ambient temperature of 27 °C and an effective sky temperature of −3 °C, taken as a clear-sky reference value. Solid bars give the flux leaving the glazing through each channel, obtained as the transport fraction multiplied by the glazing emissive power of 425 W m^{-2}. Hatched bars give the flux returning to the glazing along the same channel, obtained by reciprocity from the emissive power of the surface at the far end of that channel. The values below the axis are the difference between the two, that is the net flux carried by each channel. The channel that returns to the glazing carries the largest one-way flux of the four and yet contributes exactly zero net flux, because the surface at the far end of that channel is the glazing itself and the exchange therefore involves no temperature difference. Only the absolute values depend on the assumed temperatures; the cancellation in the self-return channel holds for any temperature.

Supplementary Text

S5. Comparison of BSDAR with a commercial venetian aluminum blind

BSDAR shares the geometry of a commercial exterior venetian blind, and this section sets out what distinguishes the two and what the distinction is worth.

Optical basis of the difference. The slats of commercial blinds are powder-coated or lacquered rather than bare aluminum, since an exterior product requires a coating for corrosion protection and color stability. A commercial blind is designed for shading and appearance, and its behavior in the atmospheric window is not among the properties it is optimized for. The device compared here is a venetian aluminum blind of this type supplied by Sonnenschutz company, whose slats we removed and reassembled so that the slat geometry and opening angle match our array exactly. Its reflectance in the atmospheric window, the band that governs the bridging function, is much lower than that of BSDAR (Figure S54), and this is where the two differ.

Measured comparison at identical geometry. With the two devices installed side by side on a real building, without wind shielding or insulation, on a hot summer day and on the south-facing orientation, only BSDAR held the window below ambient (Figure S30 and S51). The commercial blind provides no effective bridging: the window behind it remained slightly above ambient, particularly through the afternoon.

Basis of the difference. Tracing the glazing's emission through the same geometry quantifies this (Figure S55). With the specular high-reflectance slats of BSDAR, 43.5% of the emission reaches the sky and 5.0% is absorbed on the slats. With the low atmospheric-window reflectance of a commercial slat, the sky-coupled fraction falls to 22.7% and 71.6% is absorbed. A slat that cannot reflect in this band therefore absorbs the window's emission and re-radiates it at its own temperature, replacing the cold sky with a surface at ambient: the radiative cooling path is removed rather than redirected, and no tilt angle recovers it. This picture is confirmed by a separate measurement made on the same day on another south-facing window, with no PDMS coating so that the heat transferred from the slats is isolated from the radiative cooling of the emitter. The commercial blind stays above ambient throughout, its coating and aluminum substrate absorbing both the incident solar radiation and the window's own thermal emission, whereas BSDAR remains at ambient under natural convection outdoors (Figure S51).

Energy and cost consequence. The optical difference propagates to the building scale. Feeding the measured optical properties of the two devices through the same ray-tracing and EnergyPlus workflow, with identical geometry and identical weather, gives a summer cooling demand 9.3–16.1% lower with BSDAR across six cities on six continents (Figure S56): 10.3% in Guangzhou, 15.8% in Stuttgart, 9.3% in Phoenix, 10.8% in Giza, 16.1% in Buenos Aires

and 13.9% in Sydney. For the 5400 m^2 reference building, of which 560.7 m^2 is glazing, this corresponds to an annual electricity cost saving of 449–1273 USD at a coefficient of performance of 3.0 and local commercial tariffs, or 22–64 thousand USD per year for a community of fifty such buildings (Figure S57).

BSDAR is at present more expensive to produce than a commercial blind, and the premium lies entirely in the slat surface. The reflector uses a physical vapor deposited silver layer with a TiO_2 overcoat on anodized aluminum, the same construction as high-reflectance reflector sheet already manufactured at industrial scale, so the additional cost is that of a coating rather than of a new production route. The support design, tilt mechanism, installation practice and certified wind-resistance class are those of an established exterior blind and carry no premium at all, so a large difference in the unit price of the slat material corresponds to a much smaller difference in the installed cost of the device. Dividing the premium by the energy saving gives a payback of 5.5 years in the most favorable of the six cities and longer where electricity is subsidized (Figure S58), which is short against the service life of a building envelope component. Since BSDAR is externally mounted and does not require the facade or the glazing unit to be rebuilt, it also avoids the renovation cost that a material-based façade treatment would incur; the relevant comparison is therefore between a coating premium and the cost of the whole installed system rather than between the unit prices of two slat materials.

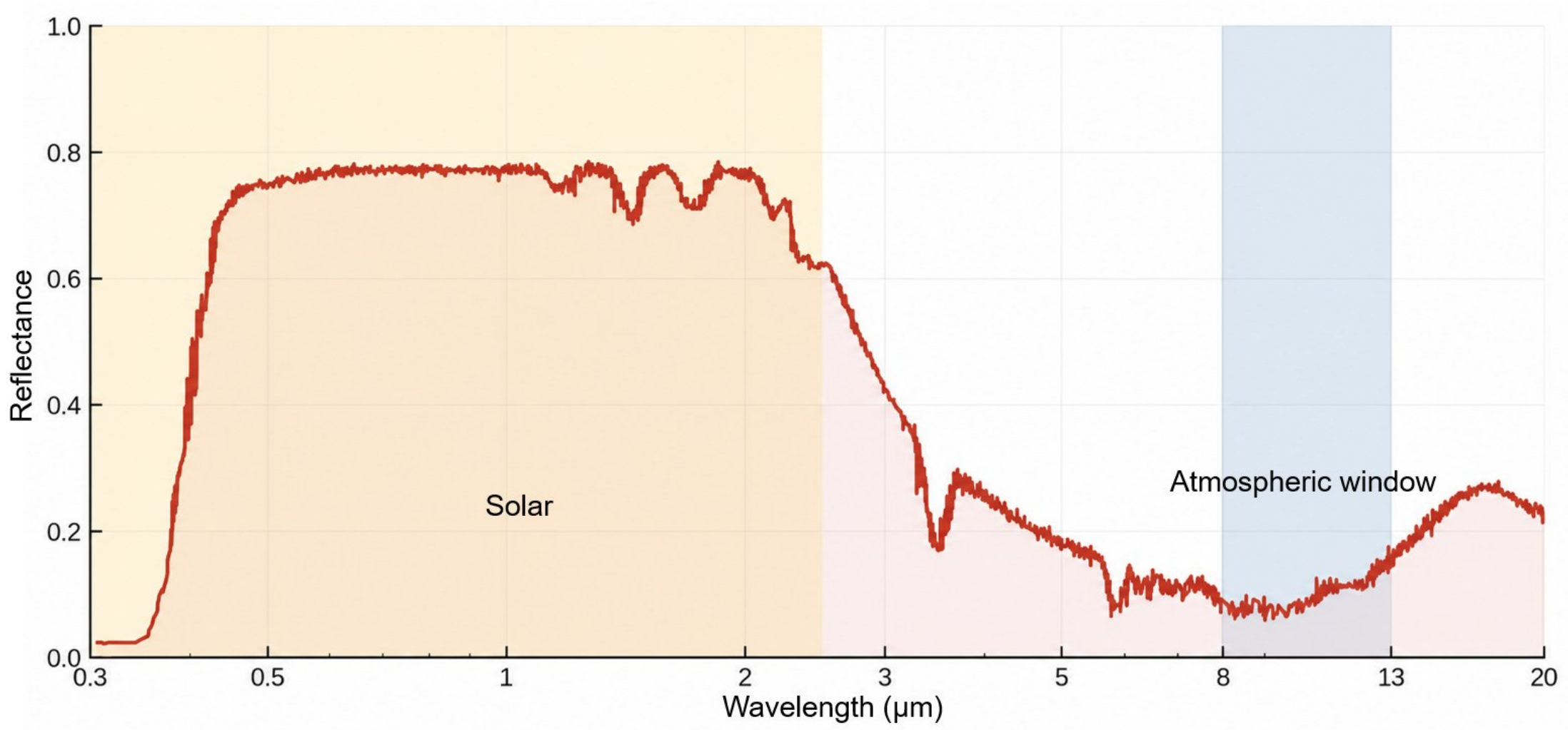

Figure S54. Hemispherical reflectance of slat surfaces of the commercial blind (*Sonnenschutz*).

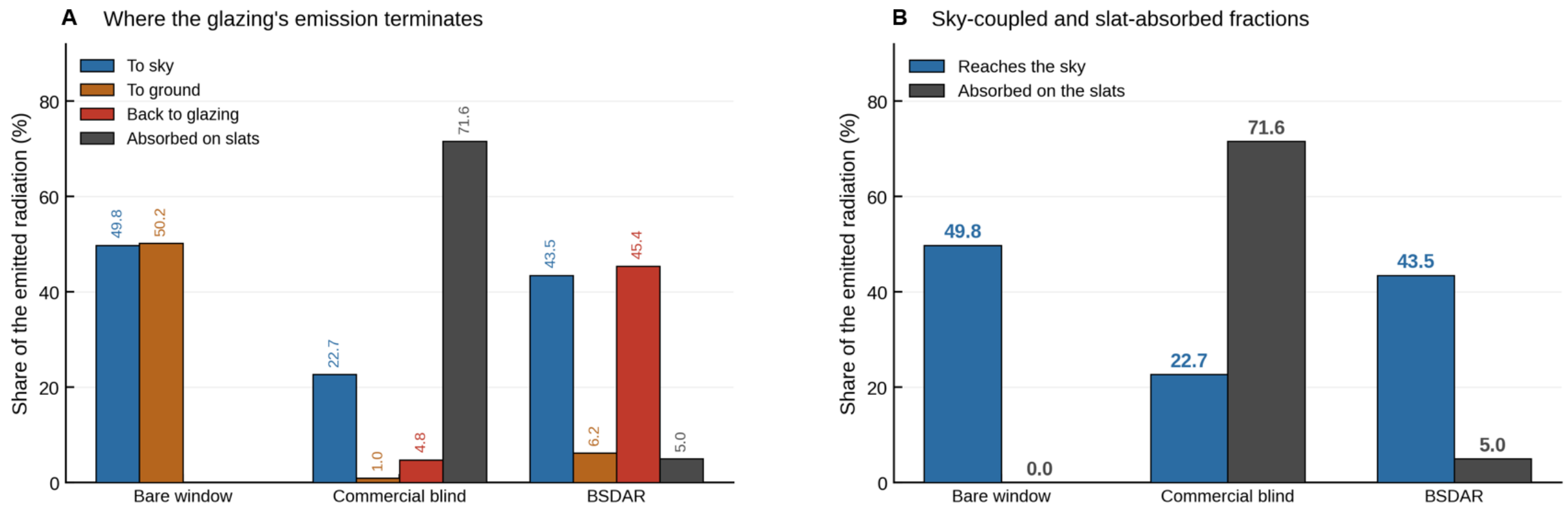

Figure S55. Infrared transport through a bare window, a commercial blind and BSDAR. Ray tracing of the thermal emission of the PDMS-coated glazing through each configuration, with 120000 rays launched from the glazing with cosine-weighted directions and the same random seed for all three, so that the three cases differ only in the slat optical properties. Slat geometry is identical for the two devices; the commercial blind and BSDAR's optic parameters are assigned from the measured values. Each ray is followed until it terminates by reaching the sky, reaching the ground, returning to the glazing, or being absorbed on a slat, and the four fractions therefore sum to unity. (**A**) All four terminal fractions for the three configurations. (**B**) The two fractions that determine the bridging function. With BSDAR, 43.5% of the emission reaches the sky and 5.0% is absorbed on the slats; with high emissivity of commercial blinds, the sky-coupled fraction falls to 22.7% while 71.6% is absorbed. The bare window, with no array, sends 49.8% upward and 50.2% downward by hemispherical symmetry.

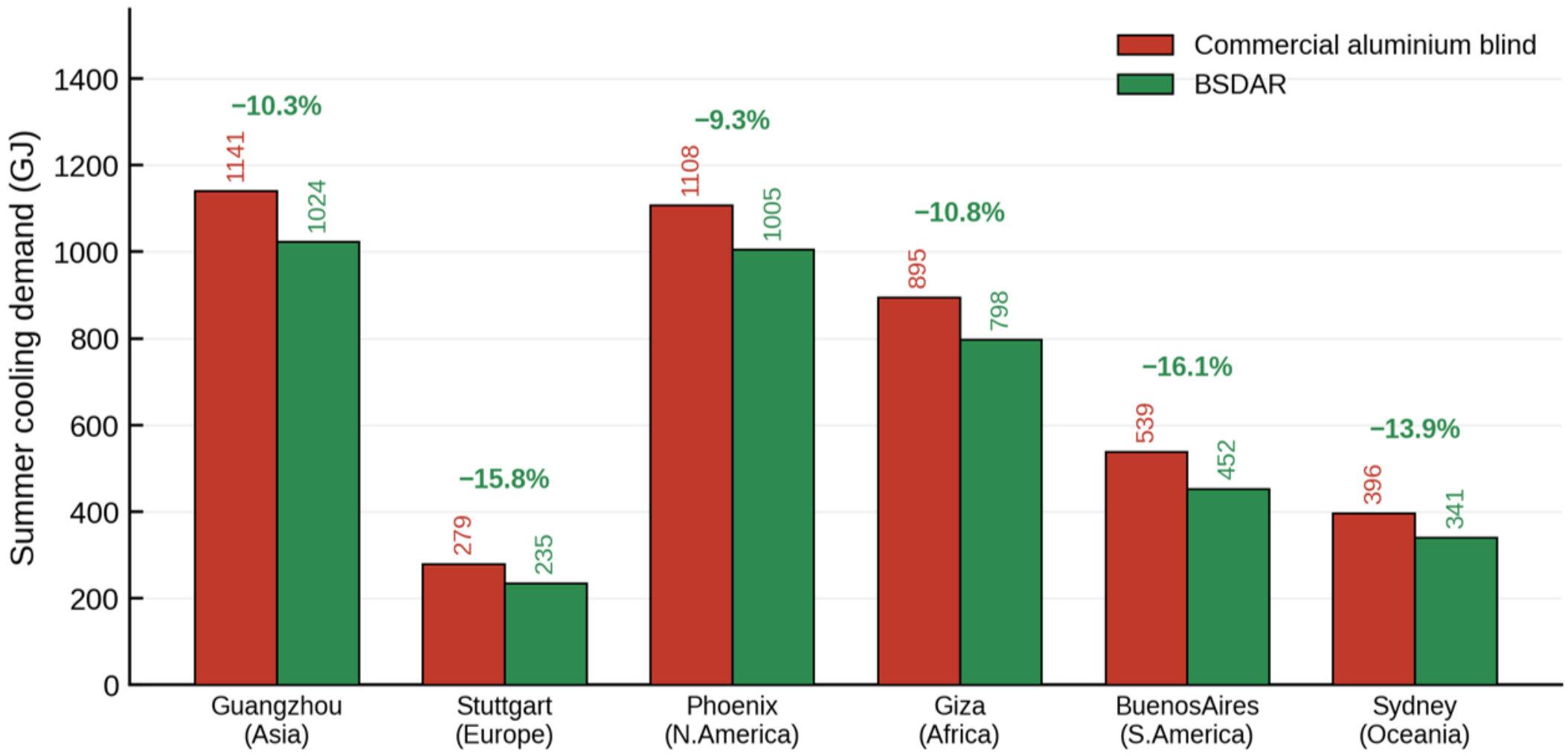

Figure S56. Summer cooling demand with BSDAR and with a commercial blind. EnergyPlus simulations of the 55-zone building for six cities on six continents. The two devices are modelled with identical slat geometry and differ only in slat optical properties: those of BSDAR and those of the commercial blind are taken from the measurements. The directional radiative exchange of each configuration was obtained by ray tracing and applied through the Energy Management System. Labels above each pair give the reduction achieved by BSDAR compared to commercial aluminum blinds.

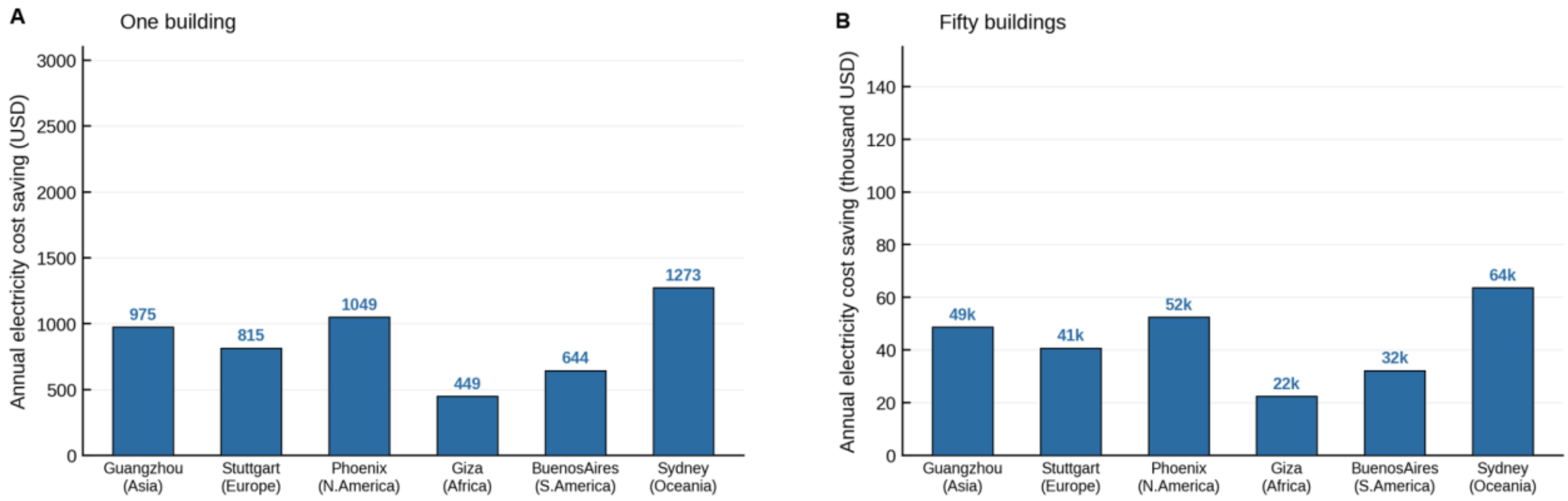

Figure S57. Annual electricity cost saving of BSDAR relative to a commercial blind. The cooling demand difference converted to electricity by dividing by a coefficient of performance of 3.0, and to cost using indicative commercial tariffs of 0.09, 0.20, 0.11, 0.05, 0.08 and 0.25 USD per kWh for the six cities respectively. (**A**) One reference building of 5400 m^2 floor area with 560.7 m^2 of glazing, both taken from the building model. (**B**) A community of fifty such buildings.

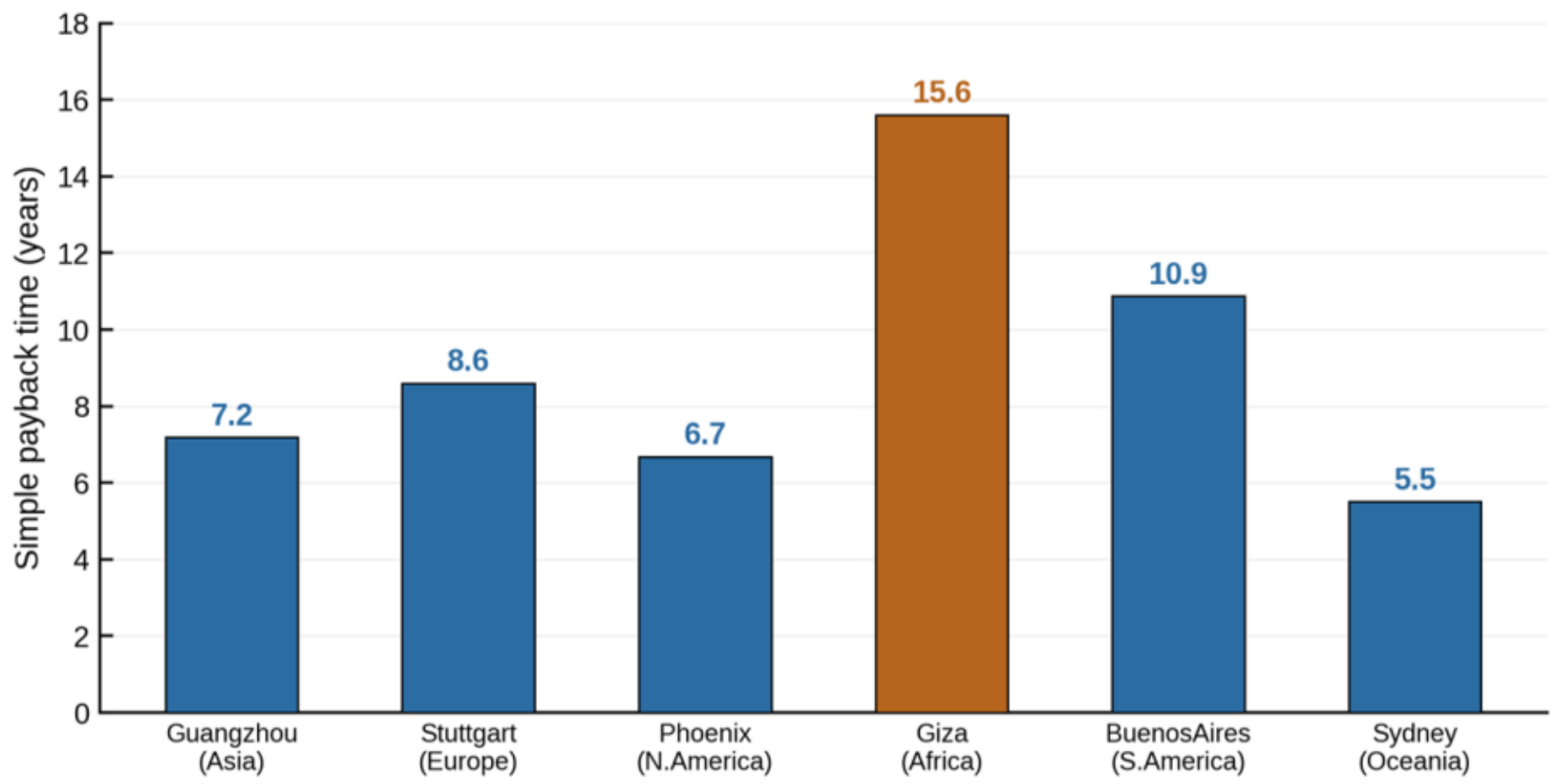

Figure S58. Payback of the BSDAR cost premium against a commercial blind. Simple payback time obtained by dividing the one-off cost premium of the BSDAR slat material over a powder-coated aluminum slat (commercial blinds) by the annual electricity cost saving. The premium is taken as 12.5 USD per m² of slat area over the 560.7 m² of glazing of the reference building, giving 7.0 thousand USD per building.

References and Notes

1. F. Xie, W. Jin, J. R. Nolen, et al., "Subambient Daytime Radiative Cooling of Vertical Surfaces," *Science* 386, no. 6723 (2024): 788–794. https://doi.org/10.1126/science.adn2524
2. Q. Cheng, S. Gomez, G. Hu, et al., "Realizing Optimal Radiative Cooling Walls in Building-Energy Nexus via Asymmetric Emissivity," *Nexus* 1 (2024): 100028. https://doi.org/10.1016/j.ynexs.2024.100028
3. M. Degeorges, J. Anand, Y. C. A. Tsang, Z. Li, N. J. Varghese, and J. Mandal, "Beyond Cooling: Radiative Thermoregulation in the Earth's Glow with Micropatterned Directional Emitters," *Joule* 9 (2025): 101956. https://doi.org/10.1016/j.joule.2025.101956
4. R. Wu, C. Sui, T. H. Chen, et al., "Spectrally Engineered Textile for Radiative Cooling against Urban Heat Islands," *Science* 384, no. 6701 (2024): 1203–1212. https://doi.org/10.1126/science.adl0653
5. J. Mandal, J. Anand, S. Mandal, J. Brewer, A. Ramachandran, and A. P. Raman, "Radiative Cooling and Thermoregulation in the Earth's Glow," *Cell Reports Physical Science* 5 (2024): 102065. https://doi.org/10.1016/j.xcrp.2024.102065
6. Q. Chen, Z. Wang, X. Zhao, F. Fan, Y. Ma, and D. Zhao, "A Transparent Directional Thermal Emitter," *Nano Letters* 26, no. 5 (2026): 1647–1654. https://doi.org/10.1021/acs.nanolett.5c04841
